# Novel anti-inflammatory diketopiperazine alkaloids from the marine-derived fungus *Penicillium brasilianum*

**DOI:** 10.1007/s00253-024-13026-4

**Published:** 2024-02-05

**Authors:** Ya-Hui Zhang, Hui-Fang Du, Yun-Feng Liu, Fei Cao, Du-Qiang Luo, Chang-Yun Wang

**Affiliations:** 1 College of Life Sciences, Key Laboratory of Medicinal Chemistry and Molecular Diagnostics of Education Ministry of China, Hebei University, Baoding, 071002 China; 2 College of Pharmaceutical Sciences, Key Laboratory of Pharmaceutical Quality Control of Hebei Province, Hebei University, Baoding, 071002 China; 3 Laboratory for Marine Drugs and Bioproducts, Qingdao National Laboratory for Marine Science and Technology, Key Laboratory of Marine Drugs, the Ministry of Education of China, School of Medicine and Pharmacy, Institute of Evolution & Marine Biodiversity, Ocean University of China, Qingdao, 266003 China

**Keywords:** Marine-derived fungus, *Penicillium brasilianum*, Diketopiperazine alkaloid, Marfey’s method, Quantum chemical calculation, Anti-inflammatory activity

## Abstract

**Abstract:**

Diketopiperazine alkaloids have proven the most abundant heterocyclic alkaloids up to now, which usually process diverse scaffolds and rich biological activities. In our search for bioactive diketopiperazine alkaloids from marine-derived fungi, two novel diketopiperazine alkaloids, penipiperazine A (**1**) and its biogenetically related new metabolite (**2**), together with a known analogue neofipiperzine C (**3**), were obtained from the strain *Penicillium brasilianum*. Their planar structures and absolute configurations were elucidated by extensive spectroscopic analyses, ^13^C NMR calculation, Marfey’s, ECD, and ORD methods. Compound **1** featured a unique 6/5/6/6/5 indole-pyrazino-pyrazino-pyrrolo system, and its plausible biogenetic pathway was also proposed. Additionally, compounds **1**–**3** have been tested for their inflammatory activities. **1** and **2** significantly inhibited the release of NO and the expression of related pro-inflammatory cytokines on LPS-stimulated RAW264.7 cells, suggesting they could be attracting candidate for further development as anti-inflammatory agent.

**Key points:**

*• A novel diketopiperazine alkaloid featuring a unique 6/5/6/6/5 indole-pyrazino-pyrazino-pyrrolo system was isolated from the marine fungus Penicillium brasilianum.*

*• The structure of* ***1*** *was elucidated by detailed analysis of 2D NMR data,* ^*13*^ *C NMR calculation, Marfey’s, ECD, and ORD methods.*

*• Compounds* ***1*** *and* ***2*** *significantly inhibited the release of NO and the expression of related pro-inflammatory cytokines on LPS-stimulated RAW264.7 cells.*

**Graphical Abstract:**

**Figure:**
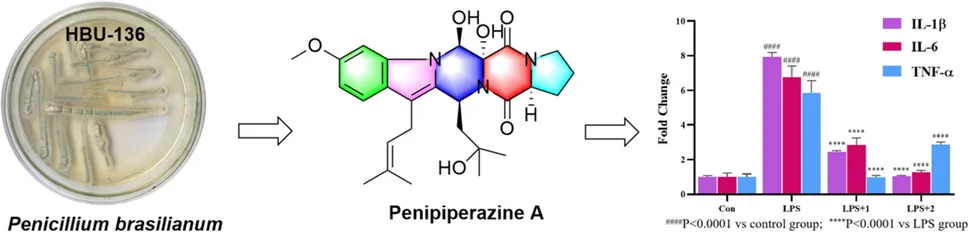

**Supplementary Information:**

The online version contains supplementary material available at 10.1007/s00253-024-13026-4.

## Introduction

Indole alkaloids, one of the largest families of secondary metabolite, are widely distributed in plant and fungi, which have proven to be an important resource of drugs (Liu and Qin 2019). As privileged structures, indole alkaloids with 2,5-diketopiperazine (DKP) scaffolds present significant biological activities and complex chemical structures involving fused heterocycles, prenylation, polythiobridging, dimerization, and oxidation (Borgman et al. 2019; Mishra et al. 2017; Borthwick 2012). Naturally occurring indole dikepiperazine alkaloids are characterized by condensation of certain amino acids, normally derived from tryptophan and a second amino acid, such as proline, tryptophan, phenylalanine, leucine, or histidine, which is modified through various biosynthetic processes to form structurally diverse compounds (Xu et al. 2014). For example, adenylation and thiolation domains in nonribosomal peptide synthetases (NRPSs) can incorporate L-tryptophan as a substrate to form a diketopiperazine intermediate, brevianamide F, which is then transformed into tryprostatins, spirotryprostatins, fumitremorgins, and notoamides. Indole diketopiperazine alkaloids are metabolites of microorganisms (Guo et al. 2020). They are commonly isolated from fungi, especially from the genera *Aspergillus* and *Penicillium*. Interest in indole diketopiperazines is due to their significant biological activities such as antimicrobial, antiviral, anticancer, immunomodulatory, antioxidant, and insecticidal activities. Therefore, they may have the potential to be used in drugs and/or serve as lead structures for drug development.

**Fig. 1:**
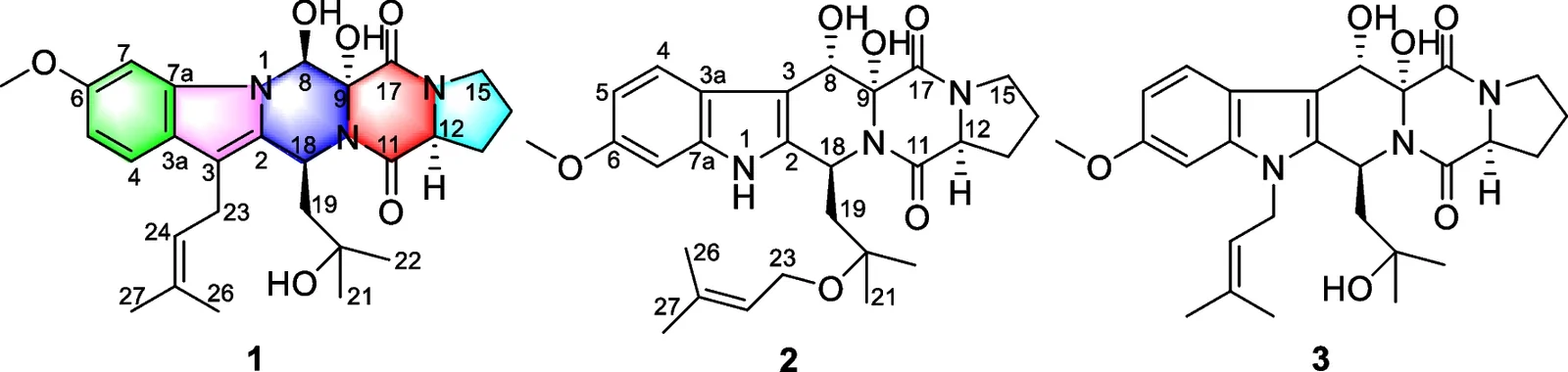
Chemical structures for **1** − **3**

During our previous research on marine fungi for new biological alkaloids, several new diketopiperazine alkaloids with antibacterial, antiviral, and cytotoxic activities were isolated (Han et al. 2023; Meng et al. 2021; Li et al. 2009; Liu et al. 2019). Among which, three indole diketopiperazines were obtained from the strain of *P. brasilianum* (HBU-136) (Zhang et al. 2019). In order to explore new bioactive diketopiperazines from the fungus HBU-136, we studied the secondary metabolites of this fungus using OSMAC strategy by changing the component of culture medium. Guided by HPLC–MS analysis and bioactivity test tracking, secondary metabolites in the EtOAc extract exhibited typical UV spectrum of indole diketopiperazines and anti-inflammatory activity. Eventually, one 6/5/6/6/5 indole-pyrazino-pyrazino-pyrrolo diketopiperazine (**1**), along with one normal indole diketopiperazine penipiperazine B (**2**), and a known analogue neofipiperzine C (**3**), were obtained (Fig. 1). These compounds were evaluated for their anti-inflammatory activities on inhibition the release of NO and the expression of related pro-inflammatory cytokines on LPS-stimulated RAW264.7 cells. Herein, we describe the isolation and structure elucidation, as well as anti-inflammatory activities of them.

## Materials and methods

### General experimental procedures

ORD data were recorded in CHCl_3_ using JASCO P-2000 spectrometer. IR spectra were acquired using KBr pellets on a Nicolet-Nexus-470 spectrometer. ECD and UV spectra were detected using MeOH as the solvent by a JASCO J-715 and a Perkin-Elmer model 241 spectrophotometer, respectively. HRESIMS spectra were recorded on a Bruker apex-ultra 7.0 T spectrometer. NMR data were obtained from a Bruker Avance 400 spectrometer using TMS as the internal standard. HPLC separation was using a C_18_ HPLC column (Waters, 10 × 250 mm, 5 μm) on the Shimadzu LC-20AT system coupled with a SPD-M20A photodiode array detector.

## Isolation of the fungal material

The fungal strain *Penicillium brasilianum* HBU-136 has been previously described (Zhang et al. 2019), which was identified by morphological characteristics, amplification, and sequencing of ITS gene sequences. The NCBI GenBank accession number was MH377073. The strain was cultured on rice solid medium (80 g rice, 2.6 g KCl, and 80 mL distilled H_2_O were added in a 1000-mL Erlenmeyer flask, 60 flasks) for 35 days at 28 °C. The fermented products were extracted with a mixture of CH_2_Cl_2_/MeOH (1:1, *v*/*v*) for five times, which was further concentrated *in vacuo* and extracted for three times using EtOAc/H_2_O (1:1, *v*/*v*). After evaporated to dryness, the extract (34.0 g) was divided into six fractions (Frs.1–6) by silica gel column chromatography (CC) using a gradual gradient elution of petroleum ether (PE)/EtOAc (100–0% (*v*/*v*) PE). Fr.3 was chromatographed by silica gel CC eluting with PE/EtOAc (1:1, *v*/*v*) to yield three subfractions (Frs.31–33). Fr.31 was further subjected to ODS silica gel (MeOH/H_2_O, 30–100% (*v*/*v*)), Sephadex LH-20 (CH_2_Cl_2_/MeOH, 1:1, *v*/*v*), and semi-preparative HPLC (70% MeOH/H_2_O, 2.0 mL/min) to yield **2** (4.0 mg) and **3** (2.2 mg). Fr.4 was separated by a Sephadex LH-20 CC (CH_2_Cl_2_/MeOH, 1:1, *v*/*v*) to give two subfractions (Frs.41–42). Fr.41 was then chromatographed on silica gel CC eluting with PE/EtOAc (1:1, *v*/*v*). Subsequently, through the final purification by semi-preparative HPLC (70% MeOH/H_2_O, 2.0 mL/min), to afford **1** (2.4 mg).

*Penipiperazine A (****1****)*: white amorphous powder; [*α*]_D_^20^ − 107.02 (*c* 0.1, CHCl_3_); IR (KBr) *v*_max_ 3109, 1710, and 1408 cm^−1^; UV (MeOH) *λ*_max_ (log *ε*) 199 (3.98), 242 (3.04), 264 (3.24), 277 (3.07), 287 (3.12), 310 (1.88) nm, see Fig. S19; ECD (1.00 mM, MeOH) *λ*_max_ (Δ*ε*) 199 (+ 4.89), 229 (− 7.02), 270 (+ 1.57), 292 (+ 0.08) nm; ^1^H and ^13^C NMR, see Table 1; HRESIMS *m*/*z* 520.2407 [M + Na]^+^ (calcd for C_27_H_35_N_3_O_6_Na, 520.2418 [M + Na]^+^).

**Table 1:** ^1^H (400 MHz) and ^13^C (100 MHz) NMR Data of **1** and **2** in CDCl_3_.

no	1		2	
	*δ*_C_, type	*δ*_H_ (*J* in Hz)	*δ*_C_, type	*δ*_H_ (*J* in Hz)
1-NH	-	-	-	9.22, s
2	129.1, C	-	132.4, C	-
3	112.2, C	-	104.6, C	-
3a	122.8, C	-	120.9, C	-
4	120.7, CH	7.42, d (8.8)	121.2, CH	7.79, d (8.4)
5	110.9, CH	6.81, dd (8.8, 2.0)	109.6, CH	6.77, dd (8.4, 1.8)
6	157.4, C	-	156.4, C	-
7	92.9, C	6.96, d (2.0)	94.9, CH	6.70, d (1.8)
7a	138.0, C	-	137.4, C	-
8	77.8, CH	6.24, s	68.7,CH	5.70, s
9	84.4, C	-	83.4, C	-
11	172.4, C	-	171.4, C	-
12	59.6, CH	4.46, m	58.9, CH	4.44, dd (8.8, 6.8)
13	29.0, CH_2_	2.07, m	29.3, CH_2_	1.96, m
		2.50, m	-	2.50, m
14	22.9, CH_2_	1.97, m	22.7, CH_2_	2.05, m
		2.13, m	-	2.09, m
15	45.8, CH_2_	3.63, m	45.4, CH_2_	3.63, m
17	163.6, C	-	166.3, C	-
18	46.4, CH	5.80 dd (8.0, 4.0)	49.4, CH	5.39, d (9.6)
19	51.6, CH_2_	2.02, m	49.3, CH_2_	1.79, m
		2.46, m		1.93, m
20	69.6, C	-	75.5, C	-
21	29.9, CH_3_	1.13, s	26.9, CH_3_	1.10, s
22	30.2, CH_3_	1.25, s	24.4, CH_3_	1.60, s
23	23.5, CH_2_	3.42, m	58.0, CH_2_	3.99, dd (9.6, 7.2)
		-		3.74, dd (9.6, 7.2)
24	122.5, CH	5.22, t (6.8)	121.0, CH	5.45, t (7.2)
25	132.6, C	-	138.8, C	-
26	25.8, CH_3_	1.71, s	26.2, CH_3_	1.89, s
27	18.2, CH_3_	1.81, s	18.1, CH_3_	1.68, s
6-OCH_3_	56.0, CH_3_	3.87, s	55.7, CH_3_	3.85, s
8-OH	-	4.44, brs	-	4.60, d (2.4)
9-OH	-	1.57	-	4.09, brs

*Penipiperazine B (****2****)*: white amorphous powder; [*α*]_D_^20^ + 14.04 (*c* 0.1, CHCl_3_); IR (KBr) *v*_max_ 3106, 1704, 1387, and 1185 cm^−1^; UV (MeOH) *λ*_max_ (log *ε*) 199 (3.95), 211 (4.02), 240 (3.13), 258 (3.26), 269 (3.09), 285 (3.26) nm, see Fig. S20; ECD (0.50 mM, MeOH) *λ*_max_ (Δ*ε*) 201 (+ 5.26), 212 (− 9.87), 238 (− 0.95) nm; ^1^H and ^13^C NMR, see Table 1; HRESIMS *m*/*z* 520.2402 [M + Na]^+^ (calcd for C_27_H_35_N_3_O_6_Na, 520.2418 [M + Na]^+^).

## Preparation of Marfey’s derivatives

Compound **1** (1.0 mg) was added to 1.0 mL of 6N HCl and the solution was stirred at 110 ℃ for 12 h. The mixture was then evaporated under vacuum to dryness, and distilled H_2_O was added to remove the trace HCl. The hydrolysate (50 μL) was treated with 200 μL acetone containing 1% (*w*/*v*) FDAA and 20 μL 1N NaHCO_3_. The mixture above was stirred at 45 ℃ for 40 min and terminated by adding 20 μL of 2N HCl. Finally, it was evaporated and dissolved in 20 μL MeCN for HPLC analysis at 254 nm. By the same procedure as for preparation of Marfey’s derivative of **1**, Marfey’s derivatives of **2**, L-Proline, and D-Proline were also obtained and used for HPLC analysis.

## Computational section

The molecules of (8*S*,9*R*,12*S*,18*S*)-**1** and (8*R*,9*S*,12*R*,18*R*)-**1** were selected for ^13^C NMR, ECD, and ORD calculations. The software of ComputeVOA was used for conformational searches, resulting in 161 conformers for (8*S*,9*R*,12*S*,18*S*)-**1**, and 145 conformers for (8*R*,9*S*,12*R*,18*R*)-**1** within relative energy of 10.0 kcal/mol energy window. Gaussian 09 package was used for conformers optimization (Frisch et al. 2009) at the gas-phase B3LYP/6–31 + G(d) level to filter out the conformers with relative energy within 2.5 kcal/mol for further reoptimization at the gas-phase B3LYP/6–311 + G(d) level (Table S1 and S2). Finally, three methods of density functional theory (DFT) were applied for ^13^C NMR calculations of (8*S*,9*R*,12*S*,18*S*)-**1**. Two methods for time-dependent DFT were used for ECD calculations of (8*S*,9*R*,12*S*,18*S*)-**1** and (8*R*,9*S*,12*R*,18*R*)-**1** with 60 excited states in total. The software of SpecDis 1.64 (Bruhn et al. 2013) was applied to produce the calculated ECD curves by Boltzmann statistics with a standard deviation of 0.30 eV. ORD calculation for (8*S*,9*R*,12*S*,18*S*)-**1** and (8*R*,9*S*,12*R*,18*R*)-**1** was carried out at the B3LYP/6–311 + G(d,p) PCM-phase level with CH_3_OH as solvent. ECD calculation for the molecule of (8*S*,9*R*,12*S*,18*S*)-**2** was also carried out, with the similar process of **1**. Forty-five conformers of (8*S*,9*R*,12*S*,18*S*)-**2** within relative energy of 10.0 kcal/mol energy window were searched for conformers optimization.

## Cytotoxicity assay

The cytotoxic activity of two new compounds **1** and **2** was evaluated under the concentration of 30 µM using MTT method in vitro (Mosmann 1983). Human carcinoma cell line (A549), human hepatocellular carcinoma cells (HepG2), human gastric adenocarcinoma cell line (AGS), and human gastric cancer cell line (HGC-27) were included, with cisplatinum (DDP) as a positive control.

## Cell culture and viability assay

Murine monocytic RAW264.7 macrophages were cultivated using Dulbecco’s modified Eagle’s medium (DMEM), which was added with 1% (*v*/*v*) penicillin/streptomycin and 10% (*v*/*v*) fetal bovine serum (FBS), at 37 °C containing 5% CO_2_. RAW264.7 cells were seeded in 96-well plates for 24 h. Subsequently, compounds **1** and **2** were added to the 96-well plates above for co-incubation. After 48 h, MTT (3-(4,5-dimethylthiazol-2-yl)-2,5-diphenyl tetrazolium bromide) solution at 5.0 mg/mL was added to the culture system above to incubated under 37 °C. Four hours later, the MTT mixture above was carefully removed, and the formazan crystals were dissolved in DMSO. A microplate reader was used to detect their absorbance at 540 nm (Mosmann 1983).

## NO production inhibition assay

The production of NO was detected by the Griess assay according to the level of nitrite (NO_2_) in the medium (Green et al. 1982). RAW264.7 cells were cultured in 96-well plates, and LPS was added to induce inflammation at 1.0 μg/mL, followed which the test compounds **1** and **2** were added for different doses. The production of NO in supernatant was quantitatively determined by the Griess reaction. The microplate reader was used for the detection of the absorbance at 540 nm. The experiments were carried out in triplicate.

## Quantitative real-time PCR (qPCR) analysis

The RAW 264.7 macrophages were seeded at 3 × 10^5^ cells/mL in 6-well plates. After incubated for 24 h, the test compounds **1** and **2** were added for co-incubation for 12 h. TRIzol reagent was used to extract total RNA, which was converted to cDNA using ReverTra Ace qPCR RT Master Mix subsequently. The specific genes of real-time PCR Master Mix was amplified by cDNA and SYBR Green. The PCR primer sequences used were as follows: IL-1*β* (forward; 5′-ACTCCTTAGTCCTCGGCCA-3′, reverse; 5′-CCATCAGAGGCAAGGAGGAA-3′), IL-6 (forward; 5′-GAGGATACCACTCCCAACAGACC-3′, reverse; 5′-AAGTGCATCATCGTTGTTCATACA-3′), TNF-*α* (forward; 5′-TGGAACTGGCAGAAGAGG-3′, reverse; 5′-AGACAGAAGAGCGTGGTG-3′), GAPDH (forward; 5′-CACTCACGGCAAATTCAACGGCA-3′, reverse; 5′-GACTCCACGACATACTCAGCAC-3′). The relative gene expression was calculated using the comparative Ct (ΔΔCt) method. Experiments were performed in triplicate. All analyses were conducted using the GraphPad Prism software.

## Results

**Fig. 2:**
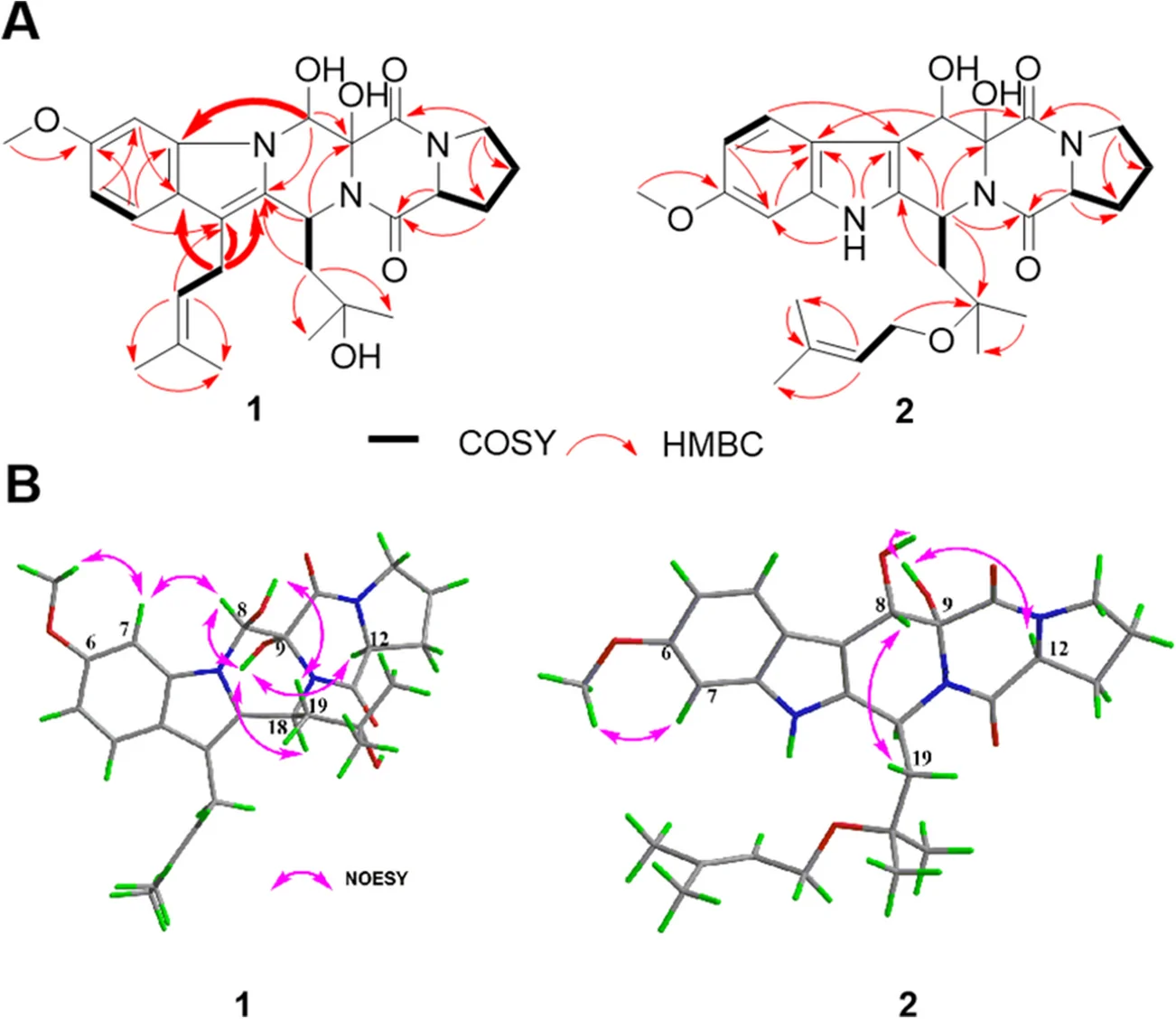
**A** COSY and Key HMBC correlations of **1** and **2**. **B** Key NOESY correlations of **1** and **2**

Penipiperazine A (**1**) was obtained as a white powder. The HRESIMS spectrum showed a positive ion at *m/z* 520.2407 (calcd for C_27_H_35_N_3_O_6_Na, 520.2418), suggesting twelve degrees of unsaturation, and its molecular formula C_27_H_35_N_3_O_6_ (Fig. S7). The ^1^H NMR data of **1** (Table 1, Fig. S1) displayed five methyls (*δ*_H_ 1.13, 1.25, 1.71, 1.81, and 3.87) including one methoxy, five aliphatic methylenes (*δ*_H_ 1.97 and 2.13, 2.02 and 2.46, 2.07 and 2.50, 3.42, and 3.63), seven methines including three aromatic proton signals (*δ*_H_ 6.81, 6.96, and 7.42), an olefinic proton signal (*δ*_H_ 5.22), an oxygen-linking methine signal (*δ*_H_ 6.24), and two nitrogen-linking methine signals (*δ*_H_ 4.46 and 5.80). From the HSQC correlations (Fig. S3), all of the proton resonances mentioned above could be attributed to 17 related carbon atoms, which were ascribed to five methyl groups, five methylenes, and seven methines. The other ten non-protonated carbons including two amide carbonyls could also be observed from its ^13^C NMR spectrum (Fig. S2), which are displayed in Table 1. These characteristic NMR findings suggested a diketopiperazine nucleus of **1**. The above NMR data of compound **1** shared high similarity with those of indole diketopiperazine neofipiperzine C (**3**), a known compound obtaining from the fungus strain of *Neosartorya fischeri* (Zheng et al. 2014). However, comprehensive comparison of the NMR spectra of **1** (Figs. S1-S6) with those of **3** (Figs. S16-S18) indicated that **1** possesses the different skeleton from that of **3**. For example, if the nucleus of **1** was the same indole diketopiperazine as that of **3**, both of the strong HMBC correlations in **1** (Figs. 2 and S5) from H-8 (*δ*_H_ 6.24) to C-7a (*δ*_C_ 138.0), and from H_2_-23 (*δ*_H_ 3.42) to C-3 (*δ*_C_ 112.2), were the unreasonable long-distance remote correlations, which were not taken into consideration. In contrast, the HMBC correlations were not observed in **3** from H-8 to C-7a, and from H-23 to C-3. The key HMBC correlations in **1** (Fig. 2A) from H-23 to C-2/C-3/C-3a, from H-8 to C-2/C-7a, from H-4 to C-3/C-7a, and from H-24 to C-3 revealed that the C-23 isoprene fragment was connected to position of C-3. The above deduction could be further verified by the difference in ^13^C NMR chemical shifts for compounds **1** and **3** at C-23 (*δ*_C_ 23.5 in **1** *vs. δ*_C_ 41.9 in **3**). Furthermore, the other key correlations from H-8 to C-2/C-7a/C-9 in the HMBC spectrum suggested that CH-8 was attached to *N*-1. According to the above analysis of NMR data, the planar structure of **1** was assigned as a novel 6/5/6/6/5 indole-pyrazino-pyrazino-pyrrolo system.

**Fig. 3:**
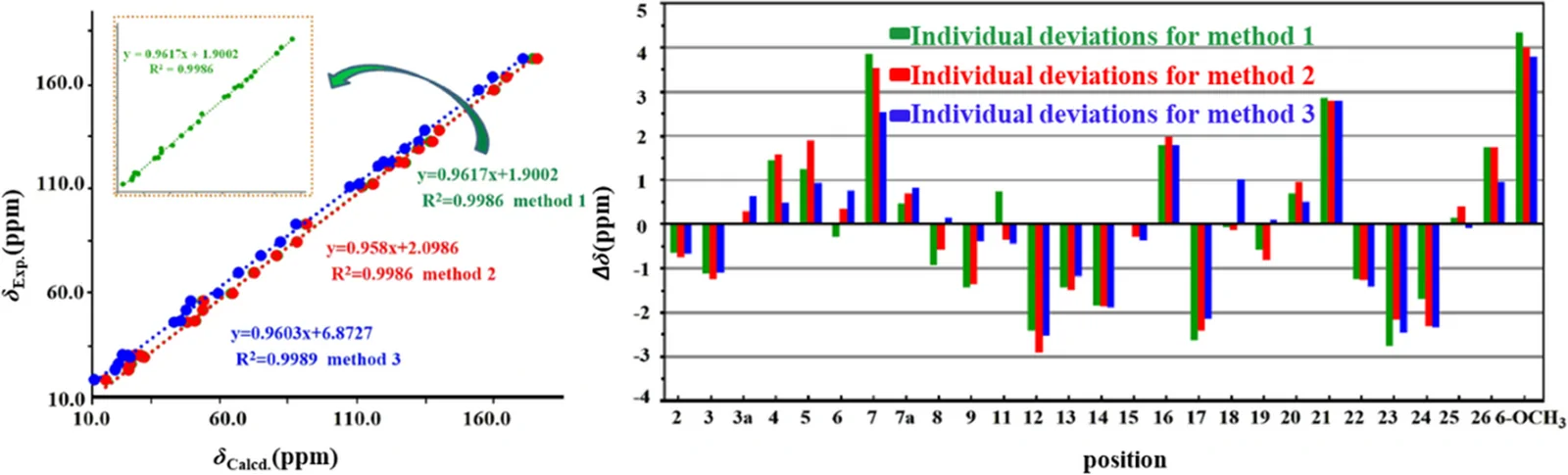
Regression analysis and individual deviations of experimental versus calculated.^13^C NMR chemical shifts of **1**

In recent years, the methods of calculating atomic chemical shift have been developed rapidly and have become a dependable approach to elucidate stereochemical configuration of complex natural compounds (Grimblat and Sarotti 2016; Marcarino et al. 2020). Therefore, to further verify the novel skeleton of **1**, according to the method of *gauge*-independent atomic orbital (GIAO), ^13^C NMR chemical shift calculation was carried out using three methods, including B3LYP/6–311 + G(d,p) (method 1), B3LYP/6–311 + G(d,p) (PCM, CHCl_3_) (method 2), and mPW1PW91/6–311 + G(d,p) (PCM, CHCl_3_) (method 3) (Hu et al. 2018). The calculated results of **1** are shown in Fig. 3, sharing high correlation coefficients (*R*^*2*^) of 0.9986, 0.9986, and 0.9989 to methods 1 − 3, respectively, indicating that the experimental *δ*_C_ of **1** was in good agreement with the calculated *δ*_C_. Furthermore, the individual deviation, |Δ*δ*|, for the experimental and calculated ^13^C NMR data of **1** was generated for three calculated methods. The maximum individual deviations were only 4.35 ppm, 3.99 ppm, and 3.78 ppm for methods 1 − 3, respectively. Thus, the carbon skeleton of **1** was definitely assigned and verified**.**

The NOESY experiment was performed to confirm the relative configuration of penipiperazine A (**1**). As with neofipiperzine C (**3**), in the NOESY spectrum of **1** (Fig. S6), the correlations from 9-OH to H-8, H-18, and H-12 of proline implied an *α*-orientation between them. Meanwhile, correlations from H-7 to 6-OCH_3_ and H-8, and from 8-OH to H_2_-19, revealed a *β*-orientation of 8-OH and 19-isopentyl group (Fig. 2B).

**Fig. 4:**
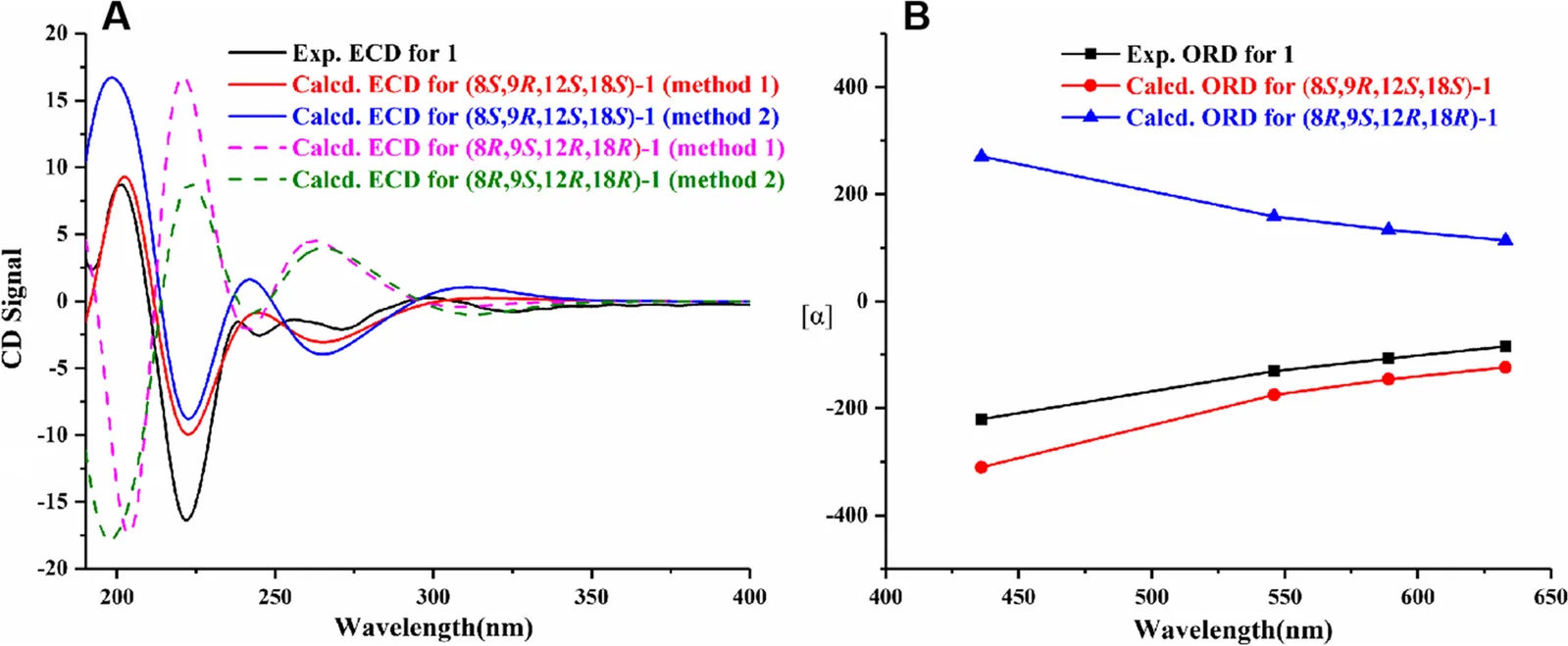
**A** Experimental and calculated ECD spectra of **1**. **B** Experimental and calculated ORD data of **1**

The absolute configuration of amino acid residue in **1** was determined according to Marfey’s method. Simultaneously, calculations of optical rotatory dispersion (ORD) and electronic circular dichroism (ECD) spectra were also applied for the configuration determination of other chiral centers. HPLC analysis (Fig. S21) showed that the proline residue was in L-configuration, meaning the *S* configuration of C-12 in **1**. On the basis of the above assignment, the molecules (8*S*,9*R*,12*S*,18*S*)-**1** and (8*R*,9*S*,12*R*,18*R*)-**1** were used for ECD and ORD calculations. Meanwhile, the ECD spectrum of **1** was measured using CH_3_OH, and the ORD data were acquired using CHCl_3_ at four wavelengths (436, 546, 589, and 633 nm). The ECD spectra of molecule (8*S*,9*R*,12*S*,18*S*)-**1** calculated with B3LYP/6–311 + G(d,p) and with B3LYP/6–311 + G(d,p) (PCM, CH_3_OH) matched well with the measured ECD curve (Fig. 4A). Also, the calculated ORD data for (8*S*,9*R*,12*S*,18*S*)-**1**, with the same wavelengths and solvent as those of the experimental ORD, were in good agreement with the experimental data (Fig. 4B). The ORD data showed negative signals, along with a positive correlation with the wavelengths (Fig. 4B). Taken together, based on the above Marfey’s, ECD and ORD data, the absolute configuration of **1** was assigned as 8*S*,9*R*,12*S*,18*S*.

**Fig. 5:**
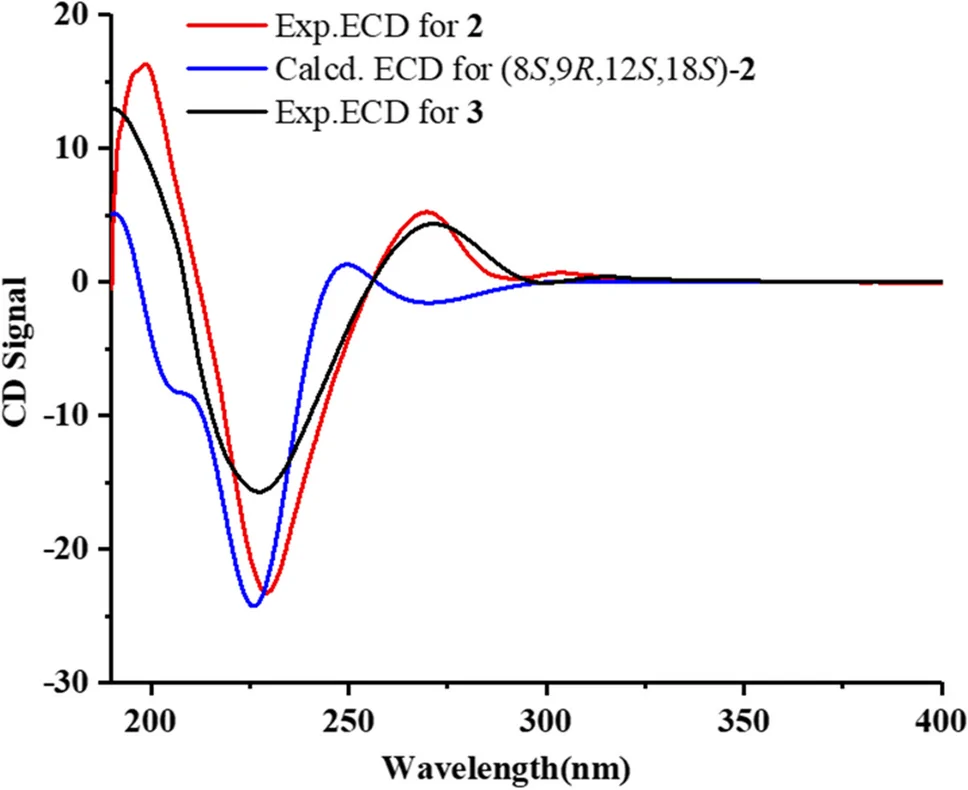
Experimental ECD spectra of **2** and **3**, and calculated ECD spectra of (8*S*,9*R*,12*S*,18*S*)-**2**

Penipiperazine B (**2**) was also obtained as white powder. The HRESIMS spectrum showed its molecular formula and degrees of unsaturation were in consistent with **3** deduced from the positive ion at *m/z* 520.2402 [M + Na]^+^ (Fig. S15). Detailed analysis of the NMR data for **2** (Figs. S8-S14) revealed that it was nearly identical with **3** (Figs. S16-S18). However, key HMBC correlations (Fig. 2A) from H_2_-23 (*δ*_H_ 3.74 and 3.99) to C-20 (*δ*_C_ 75.5) suggested that the C-23 isopentene unit was attached to C-20 via an oxygen atom rather than directly attached to *N*-1 in the indole moiety. The other key correlations observed in the HMBC spectrum of **2** made great contribution to the confirmation of this structure. The NOESY correlations and ECD spectrum of **2** were similar to those of **3** (Figs. 2B, 5, and S14), indicated that **2** showed the same configurations of C-8/9/12/18 as in **3**, which were further confirmed by ECD calculation and Marfey’s method (Figs. 5 and S21).

The third compound was assigned as neofipiperzine C (**3**) (Zheng et al. 2014) interpretation of its HRESIMS data and 1D/2D NMR spectroscopic data.

**Fig. 6:**
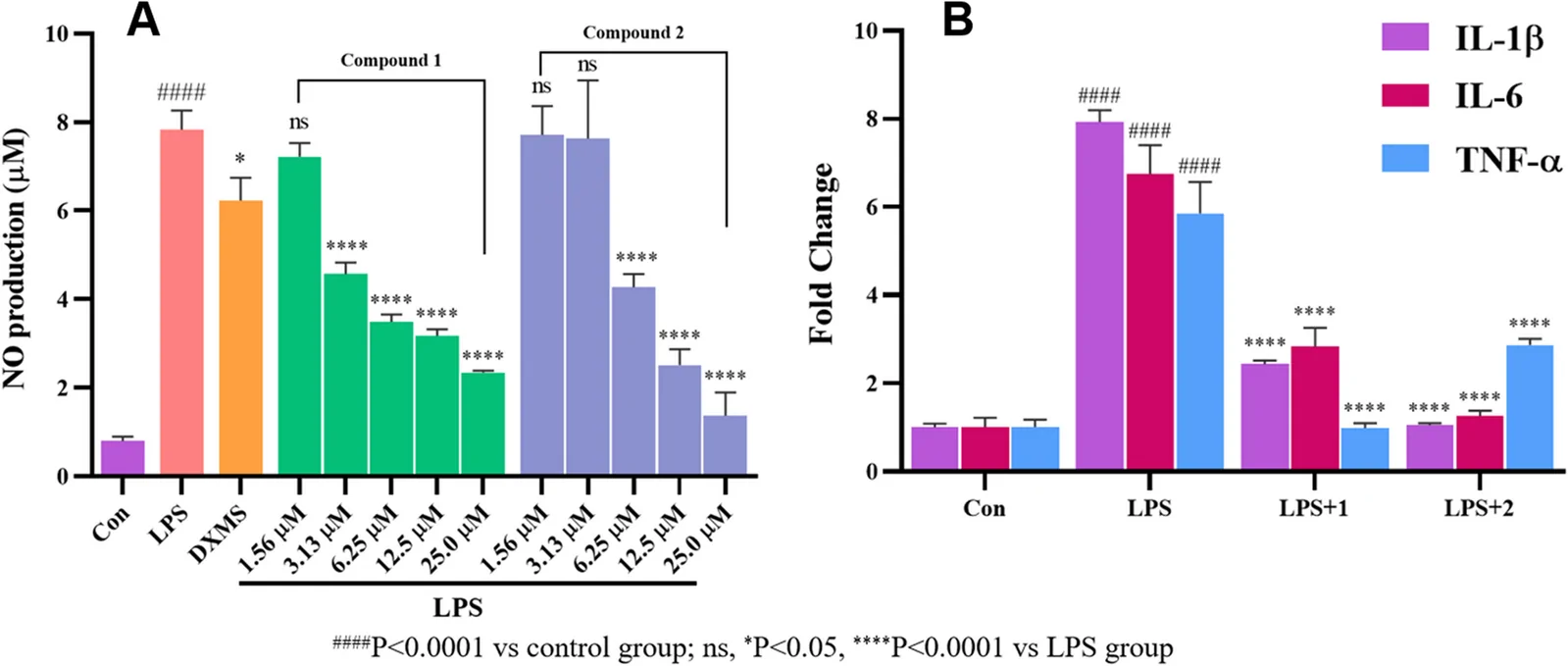
**A** Effects of **1** and **2** on NO release in LPS-induced RAW 264.7 cell. **B** Inhibitory effects of **1** and **2** on pro-inflammatory cytokines expression

Rarely reports showed diketopiperazine alkaloid with inflammatory activities. In the present study, two new compounds **1** and **2** were tested for their cytotoxic activity and for their anti-inflammatory activities. They were nontoxic towards the tested cancer cell lines (HepG2, A549, AGS, and HGC-27) at the concentration of 25.0 µM (IC_50_ > 30.0 µM) (Table S3). The anti-inflammatory activities were evaluated using RAW264.7 cells, measuring the production of nitric oxide (NO) and expression of pro-inflammatory cytokines affected by lipopolysaccharide (LPS). Before the bioassays, the cytotoxic effects of **1** and **2** were firstly determined, and both of them showed no toxicity at concentrations under 30.0 μM. Then, NO production was tested for **1** and **2**. It was showed that **1** and **2** were actively suppressed NO production (Fig. 6A). At 3.13, 6.25, 12.5, 25.0 µM, with 41.6%, 55.5%, 59.5%, 70.1% inhibition of NO production, respectively, were recorded for **1**, and at 6.25, 12.5, 25.0 µM, with 45.5%, 67.9%, 82.5% inhibition of NO production, respectively, were recorded for **2**. In order to explore their anti-inflammatory effects, pro-inflammatory cytokines expression was determined upon treatment with **1** and **2**. As shown in Fig. 6B, 1 and 2 at the concentration of 25.0 µM could markedly decrease the mRNA levels of pro-inflammatory cytokines including IL-1*β*, IL-6, and TNF-*α* in RAW264.7 cells stimulated by LPS. Treating RAW264.7 cells with **1** decreased IL-1*β*, IL-6, and TNF-*α* levels by 70.2%, 47.2%, and 83.7%, respectively, while **2** could decrease IL-1*β*, IL-6, and TNF-*α* levels by 87.4%, 82.3%, and 59.5%, respectively.

## Discussion

**Fig. 7:**
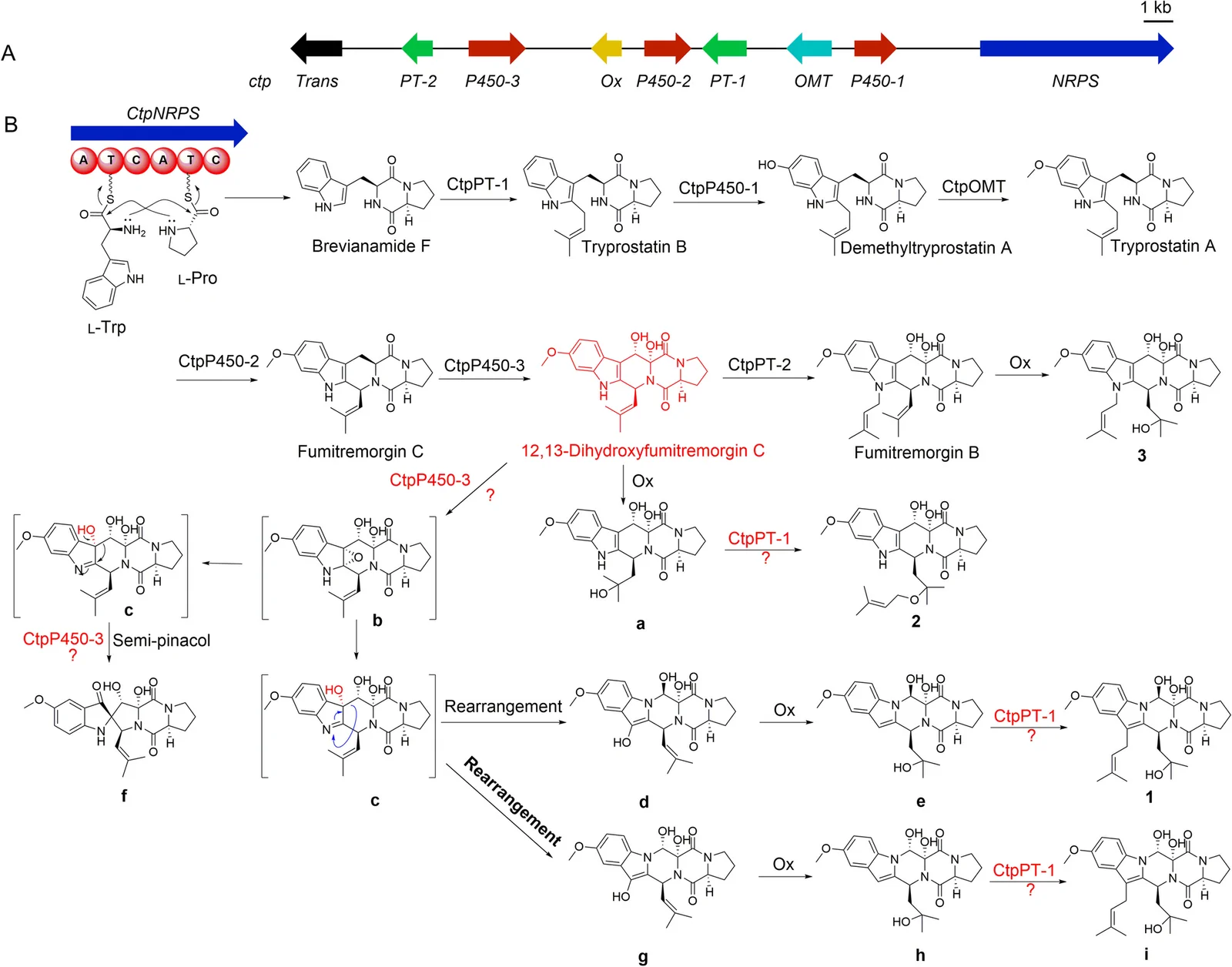
Plausible biogenetic pathway of **1** − **3**

The indole dikepiperazine skeleton usually contains a tryptophan unit (Ma et al. 2016), in which the position of the *N* atom is fixed. In the course of our recent research, a novel diketopiperazine with unprecedented carbon skeleton of 6/5/6/6/5 indole-pyrazino-pyrazino-pyrrolo system (**1**) was isolated, in which the position of the *N* atom has changed. As far as we know, penipiperazine A (**1**) is the first example of the novel diketopiperazine skeleton mentioned above. It is somewhat interesting to explore the change of *N* atom position in the biosynthetic pathway of **1**. The whole genome sequence of this fungus strain *P. brasilianum* has been sequenced, as well as the genes cluster responsible for the formation of diketopiperazine (DKP) alkaloids has been predicted, which was reported in detail in our previous research (Zhang et al. 2019). The gene cluster contains an NRPS gene (*ctp*NRPS), three cytochrome P450 genes (*ctp*P450-1/2/3), two prenyltransferase genes (*ctp*PT-1/2), an oxmethyltransferase gene (*ctp*OMT), and a putative oxidase gene (*ctp*Ox)(Zhang et al. 2019). A possible biosynthetic pathway of **1** − **3** was proposed (Fig. 7), which was started from L-Pro and L-Trp, two important precursors to the synthesis of many 2,5-diketopiperazines in fungi, catalyzed by the *ctp*NRPS gene to produce brevianamide F (Steffan et al. 2009). Then, more steps of successive reactions including hydroxylation, normal prenylation, methylation, and cyclization led to the formation of 12,13-dihydroxyfumitremorgin C (Steffan et al. 2009), which probably acts as an important biosynthetic intermediate for compounds **1** − **3**. The oxidation of the C19 = C20 double bond in 12,13-dihydroxyfumitremorgin C, probably followed by a normal prenylation reaction to produce **2** (Steffan et al. 2009; Grundmann et al. 2008). The epoxidation of the indole double bond on the C2 = C3 can be catalyzed by *ctp*P450-3 giving rise to intermediate **b**, and subsequent epoxide ring opening to form transient intermediates **c** (Tsunematsu et al. 2013). It was noteworthy that the catalytic reaction from **c** to **d** is possibly belonging to a radical-mediated type rearrangement (Tsunematsu et al. 2013). Homolytic cleavage of the C3-C9 bond of **c** leads to the formation of two free radicals, then the free radical on C3 transferred to N1 spontaneously, after which ring closure of C9 and N1 results in the formation of **d**. In the end, oxidative on the C19 = C20 double band of **d**, with a following prenylation reaction at C-3 to afford product **1**.

In summary, we report the isolation and structural identification of penipiperazine A (**1**) from a marine fungus *Penicillium brasilianum*, along with a new biogenetically related analogue and a known one. The novel diketopiperazine alkaloid **1** featured an unprecedented 6/5/6/6/5 indole-pyrazino-pyrazino-pyrrolo system. Their structures were determined through NMR spectroscopic analyses, ^13^C NMR calculation, Marfey’s, ECD, and ORD methods. In the previous studies on diketopiperazine alkaloid, the inflammatory activities were rarely reported (Liu et al. 2022; Yang et al. 2021; Wen et al. 2018). These above findings suggested that penipiperazine A had intriguing novel structure and showed potent anti-inflammatory effect. These results suggested that it is a promising lead compound for further development as an anti-inflammatory agent. Further studies of the synthetic, biosynthetic, and biological function of **1** are expected.

## Supplementary Information

Below is the link to the electronic supplementary material.

Supplementary file1 (PDF 1424 KB)

## Data Availability

This strain HBU-136 has been deposited in the China General Microbiological Culture Collection Center with its CGMCC number 40072. Full spectroscopic data of compounds **1** and **2** are included in the Supplementary data.
