# Supplementary material for: Novel anti-inflammatory diketopiperazine alkaloids from the marine-derived fungus Penicillium brasilianum
Source: Appl Microbiol Biotechnol. 2024 Feb 5;108:194. doi: 10.1007/s00253-024-13026-4 (PMC10844341; doi:10.1007/s00253-024-13026-4)
Supplement: Supplementary file 1 [file 253_2024_13026_MOESM1_ESM.pdf]

Supporting Information

Novel Anti-inflammatory Diketopiperazine Alkaloids from the Marine-Derived Fungus *Penicillium brasilianum*

Ya-Hui Zhang,[1] Hui-Fang Du,[2] Yun-Feng Liu,[1] Fei Cao,[2]* Du-Qiang Luo,[1]* and Chang-Yun Wang,[3]*

[1]College of Life Sciences, Key Laboratory of Medicinal Chemistry and Molecular Diagnostics of Education Ministry of China, Hebei University, Baoding 071002, China.

[2]College of Pharmaceutical Sciences, Key Laboratory of Pharmaceutical Quality Control of Hebei Province, Hebei University, Baoding 071002, China

[3]Laboratory for Marine Drugs and Bioproducts, Qingdao National Laboratory for Marine Science and Technology; Key Laboratory of Marine Drugs, the Ministry of Education of China, School of Medicine and Pharmacy, Institute of Evolution & Marine Biodiversity, Ocean University of China; Qingdao 266003, China

*Correspondence: F.C. (caofei542927001@163.com), D.Q.L. (duqiangluo@hbu.edu.cn) and C.Y.W. (changyun@ouc.edu.cn)

List of Supporting Information

Fig. S1 ^{1}H NMR (400 MHz, $CDCl_3$) spectrum of compound **1**.

Fig. S2 ^{13}C NMR (100 MHz, $CDCl_3$) spectrum of compound **1**.

Fig. S3 HSQC ($CDCl_3$) spectrum of compound **1**.

Fig. S4 ^{1}H–^{1}H COSY ($CDCl_3$) spectrum of compound **1**.

Fig. S5 HMBC ($CDCl_3$) spectrum of compound **1**.

Fig. S6 NOESY ($CDCl_3$) spectrum of compound **1**.

Fig. S7 HRESIMS spectrum of compound **1**.

Fig. S8 ^{1}H NMR (400 MHz, $CDCl_3$) spectrum of compound **2**.

Fig. S9 ^{13}C NMR (100 MHz, $CDCl_3$) spectrum of compound **2**.

Fig. S10 HSQC ($CDCl_3$) spectrum of compound **2**.

Fig. S11 ^{1}H–^{1}H COSY ($CDCl_3$) spectrum of compound **2**.

Fig. S12 HMBC ($CDCl_3$) spectrum of compound **2**.

Fig. S13 Partial HMBC ($CDCl_3$) spectrum of compound **2**.

Fig. S14 NOESY ($CDCl_3$) spectrum of compound **2**.

Fig. S15 HRESIMS spectrum of compound **2**.

Fig. S16 ^{1}H NMR (600 MHz, $CDCl_3$) spectrum of compound **3**.

Fig. S17 ^{13}C NMR (150 MHz, $CDCl_3$) spectrum of compound **3**.

Fig. S18 HMBC ($CDCl_3$) spectrum of compound **3**.

Fig. S19 Experimental UV spectrum of **1**.

Fig. S20 Experimental UV spectrum of **2**.

Fig. S21 HPLC at 254 nm of the Marfey's analysis.

Table S1 The coordinate for the lowest-energy conformer of compound **1** in ^{13}C NMR, ECD, and ORD calculations.

Table S2 The coordinate for the lowest-energy conformer of compound **2** for ECD calculation.

Table S3 Cytotoxic activity data of compounds **1** and **2**.

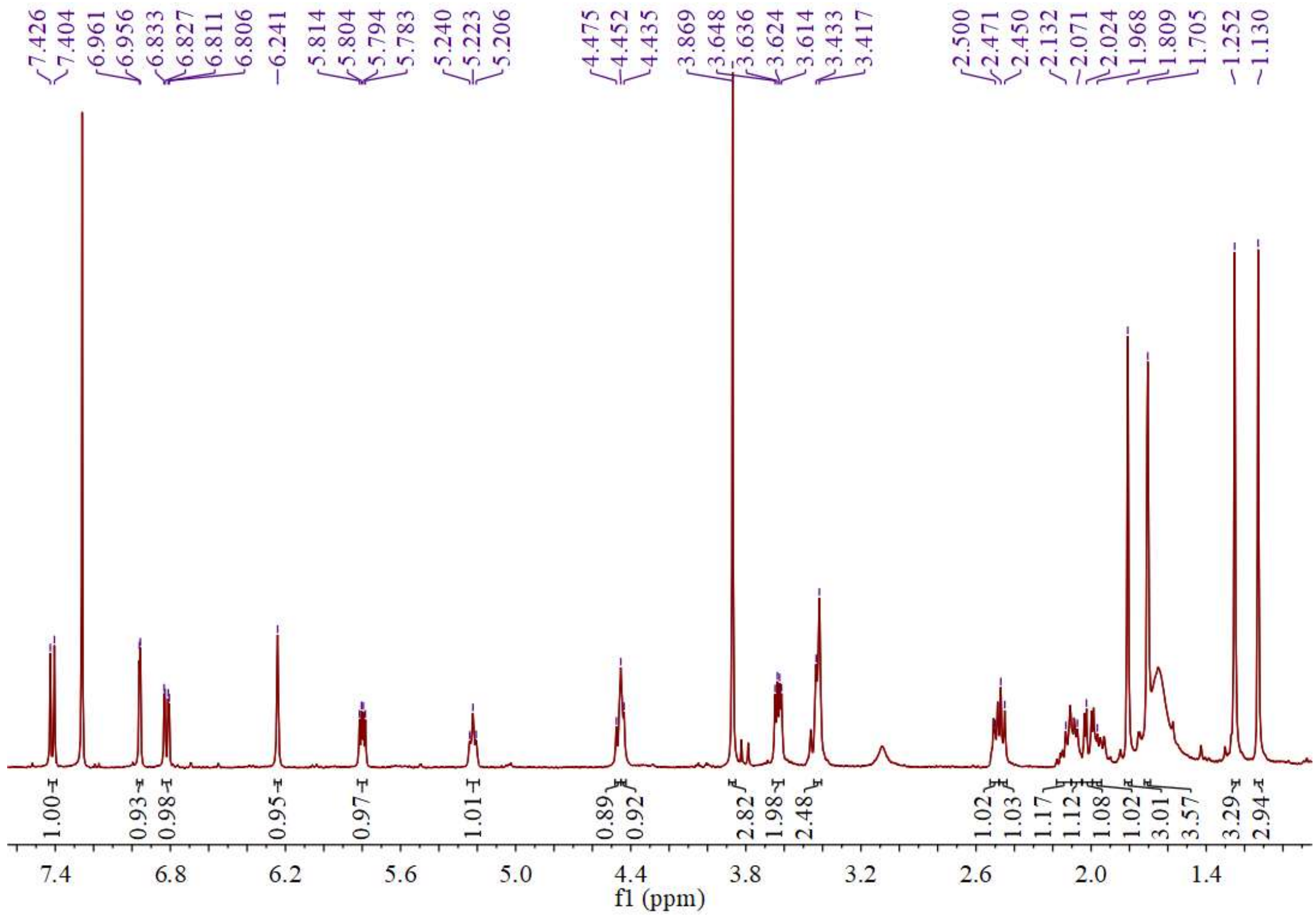

Fig. S1 ^{1}H NMR (400 MHz, $CDCl_3$) spectrum of compound **1**.

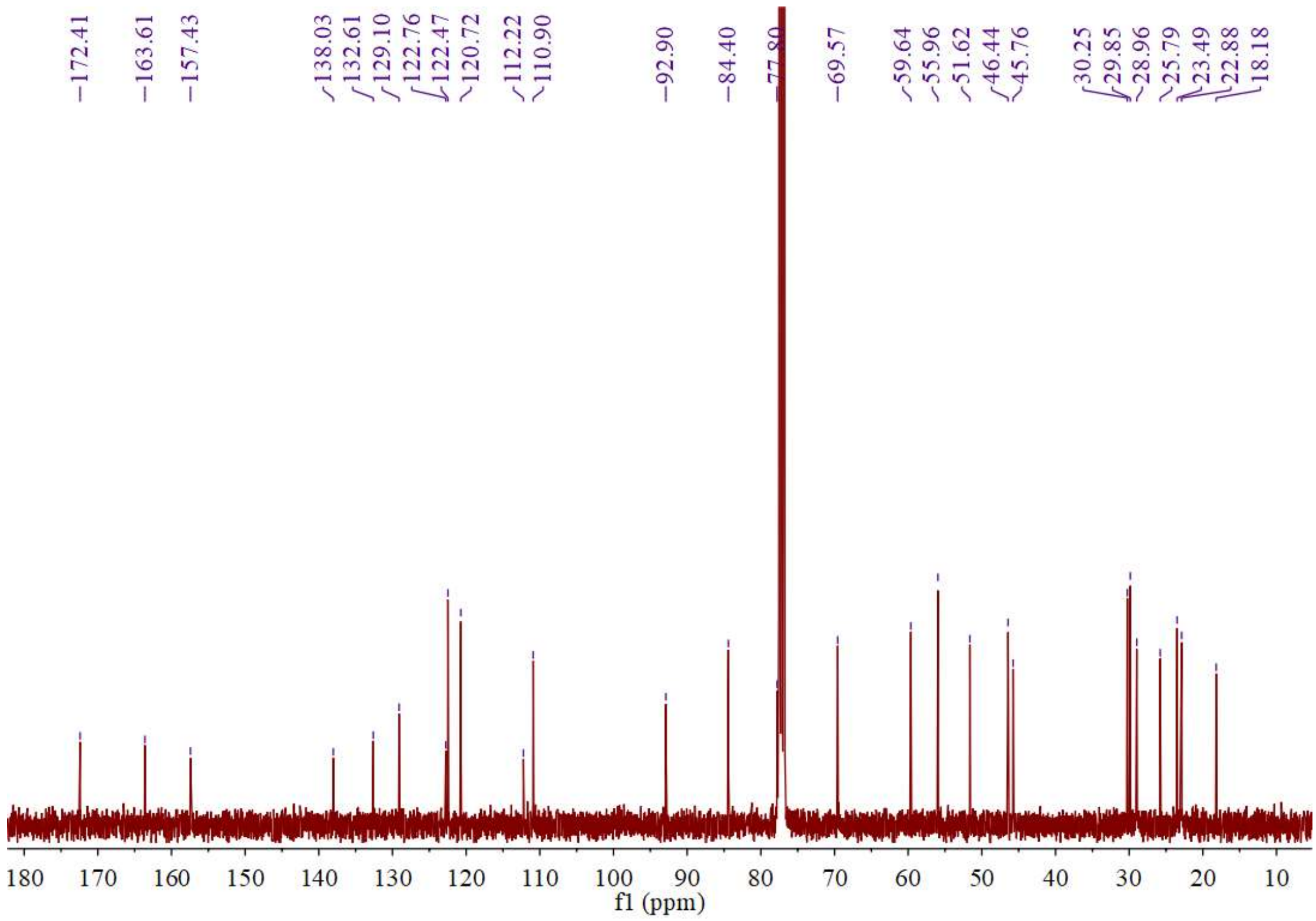

Fig. S2 ^{13}C NMR (100 MHz, $CDCl_3$) spectrum of compound **1**.

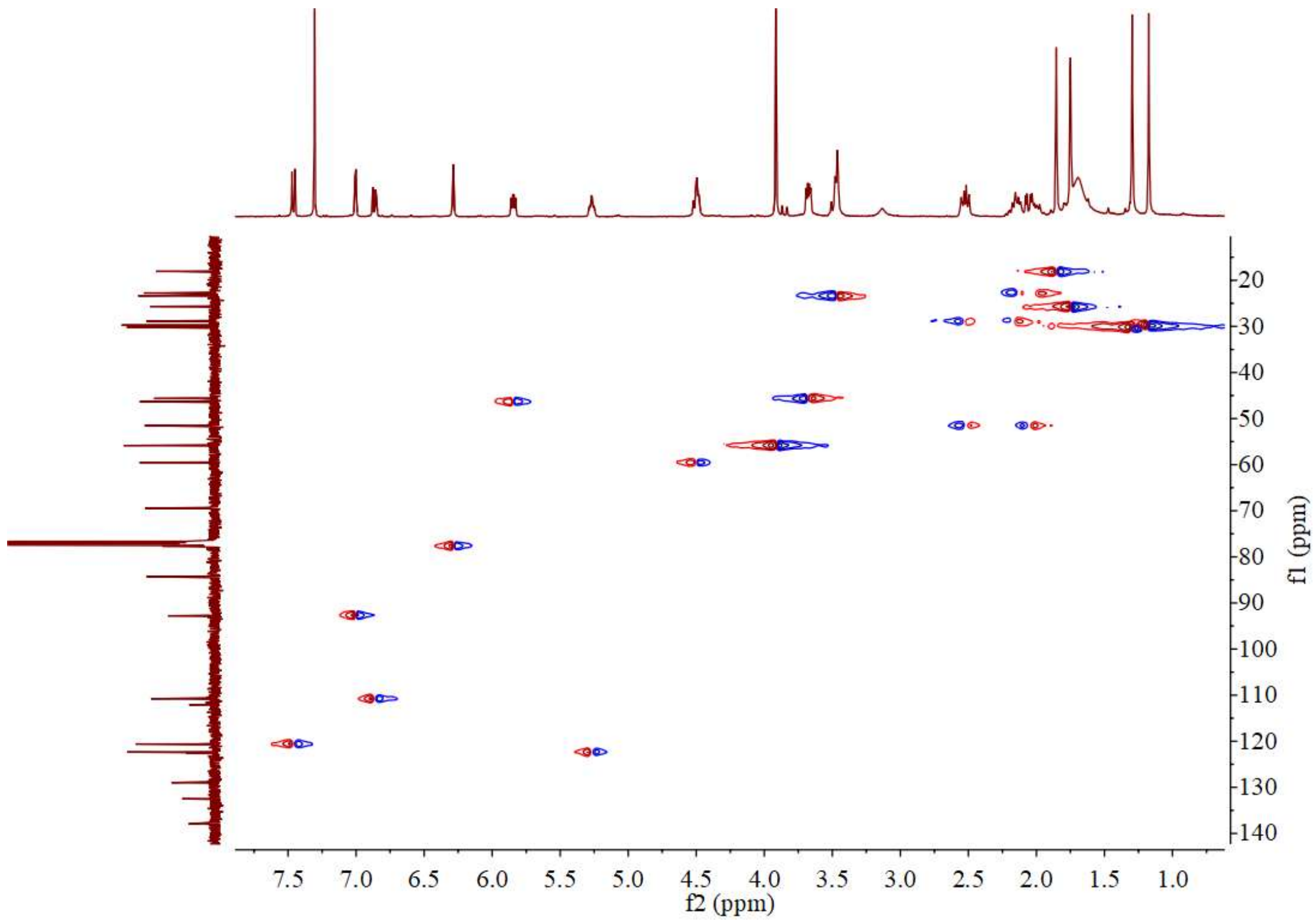

Fig. S3 HSQC ($CDCl_3$) spectrum of compound **1**.

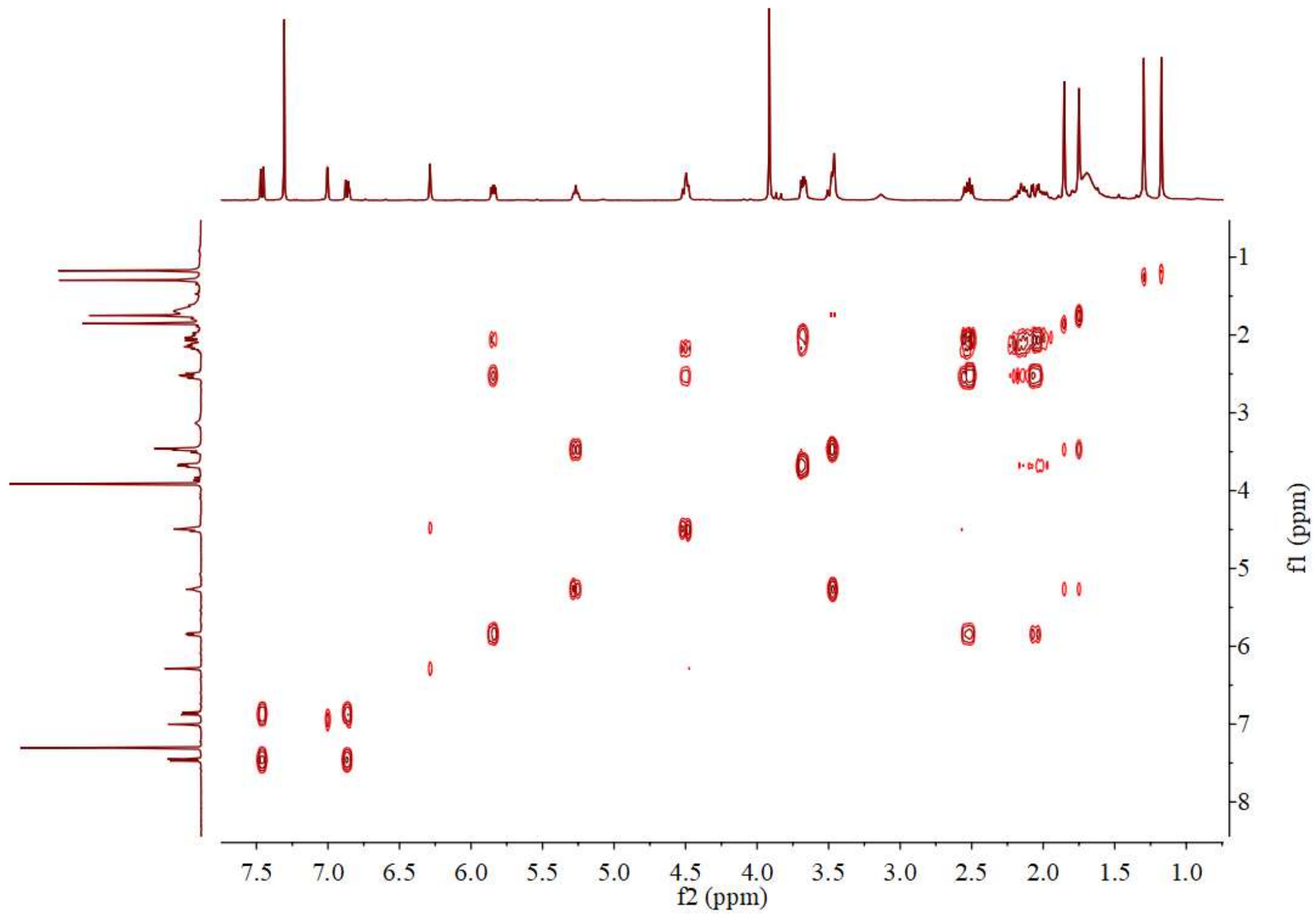

Fig. S4 ^1H–^1H COSY ($CDCl_3$) spectrum of compound **1**.

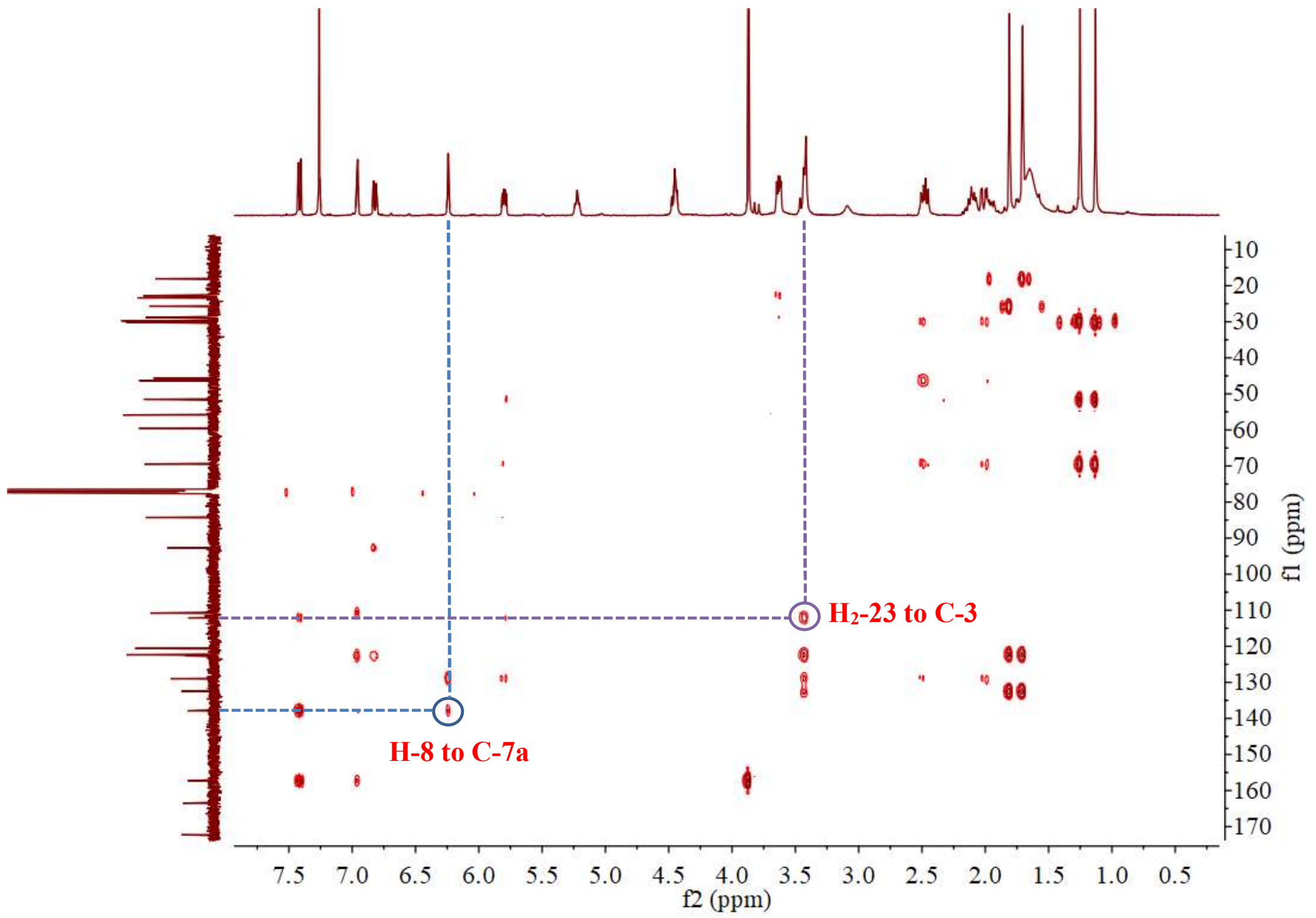

Fig. S5 HMBC ($CDCl_3$) spectrum of compound **1**.

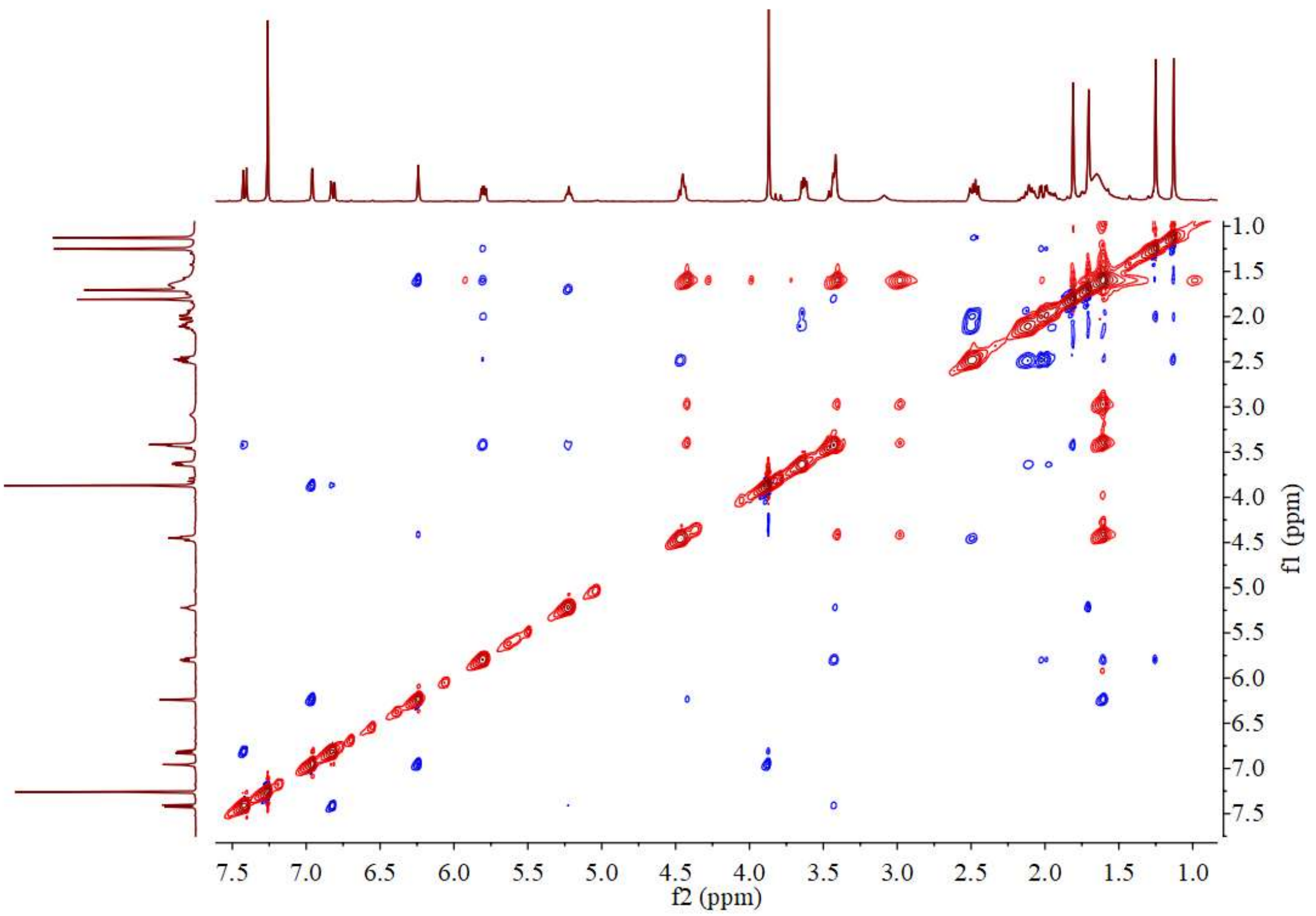

Fig. S6 NOESY ($CDCl_3$) spectrum of compound **1**.

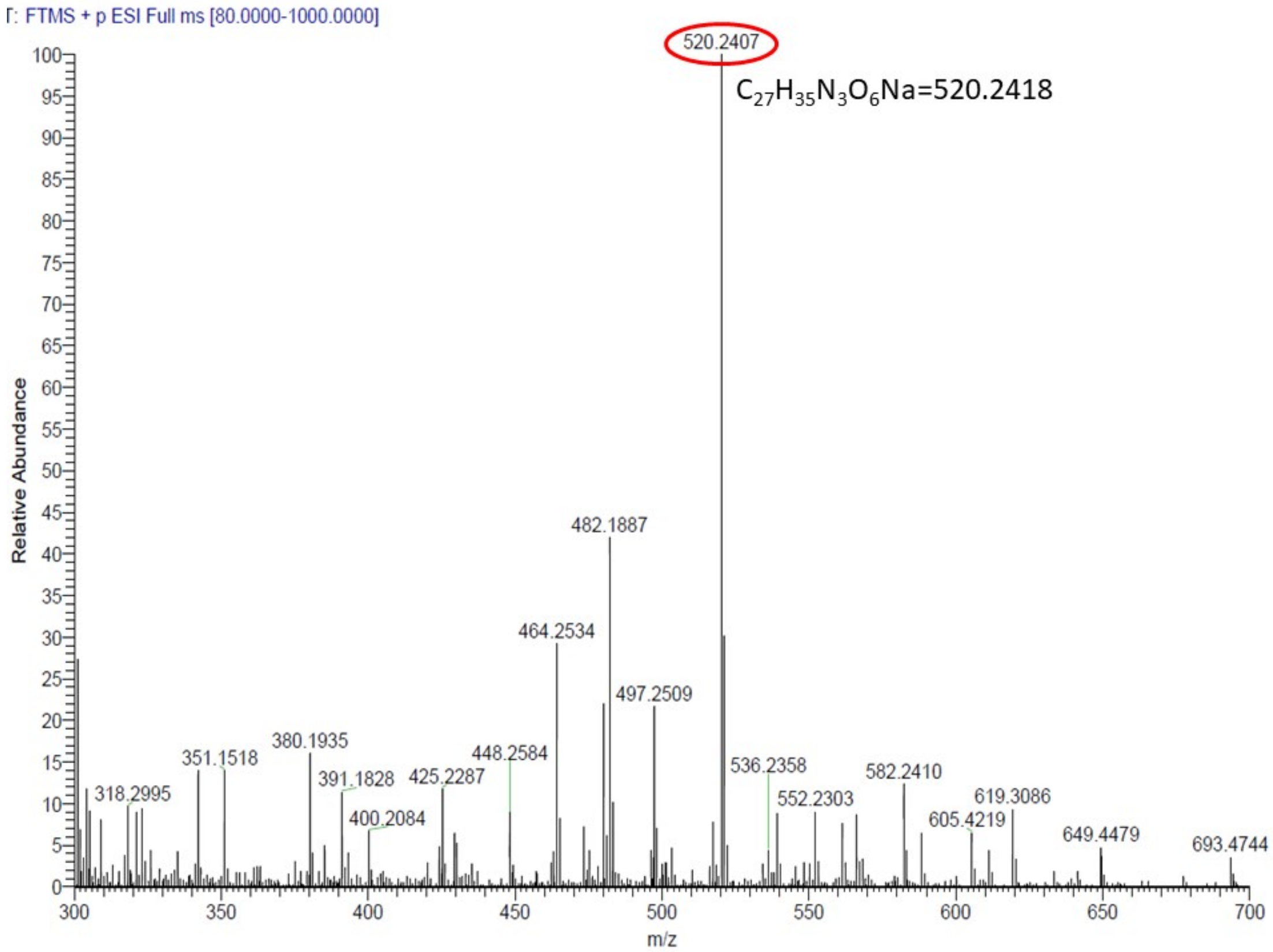

Fig. S7 HRESIMS spectrum of compound **1**.

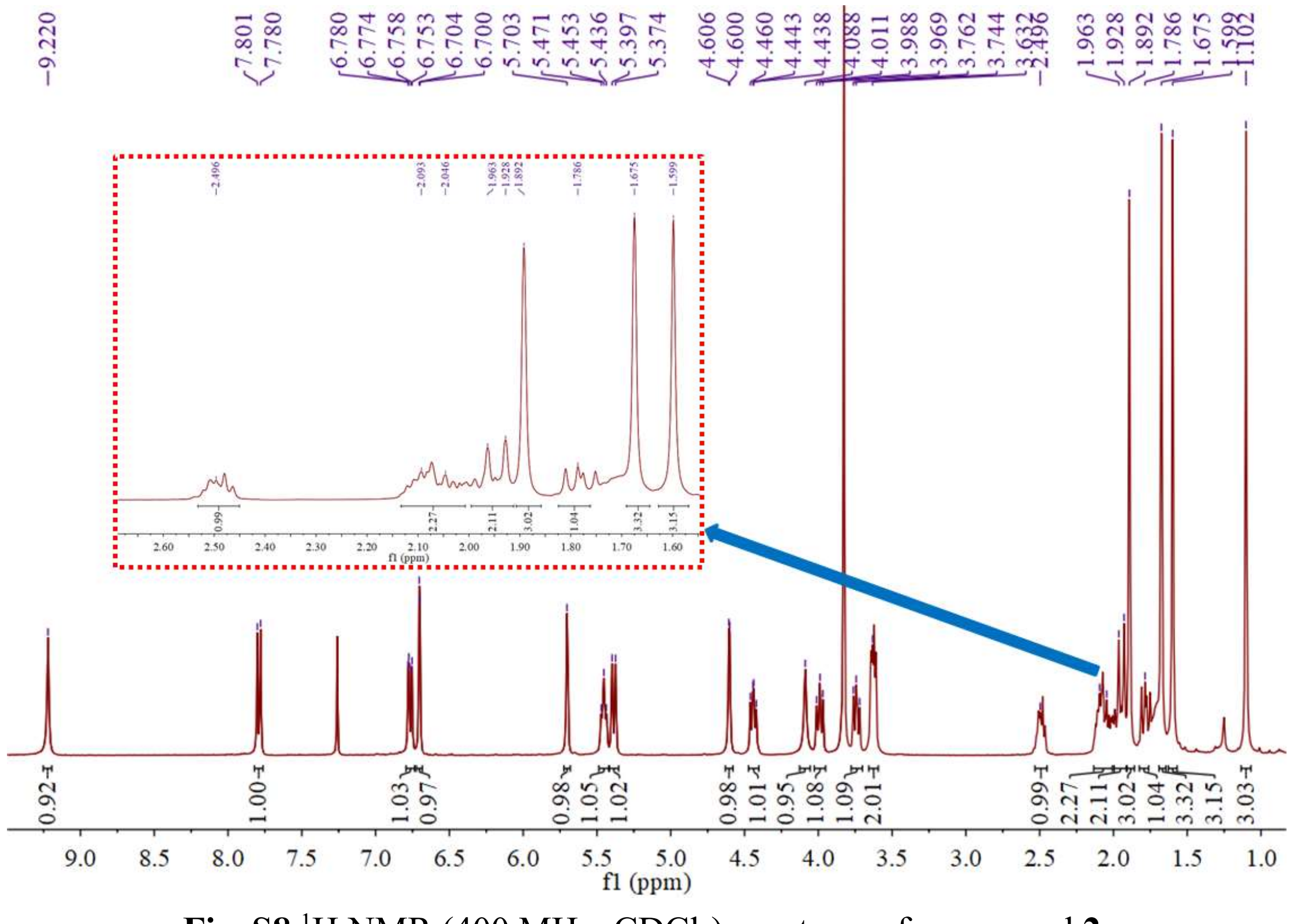

Fig. S8 ^{1}H NMR (400 MHz, $CDCl_3$) spectrum of compound **2**.

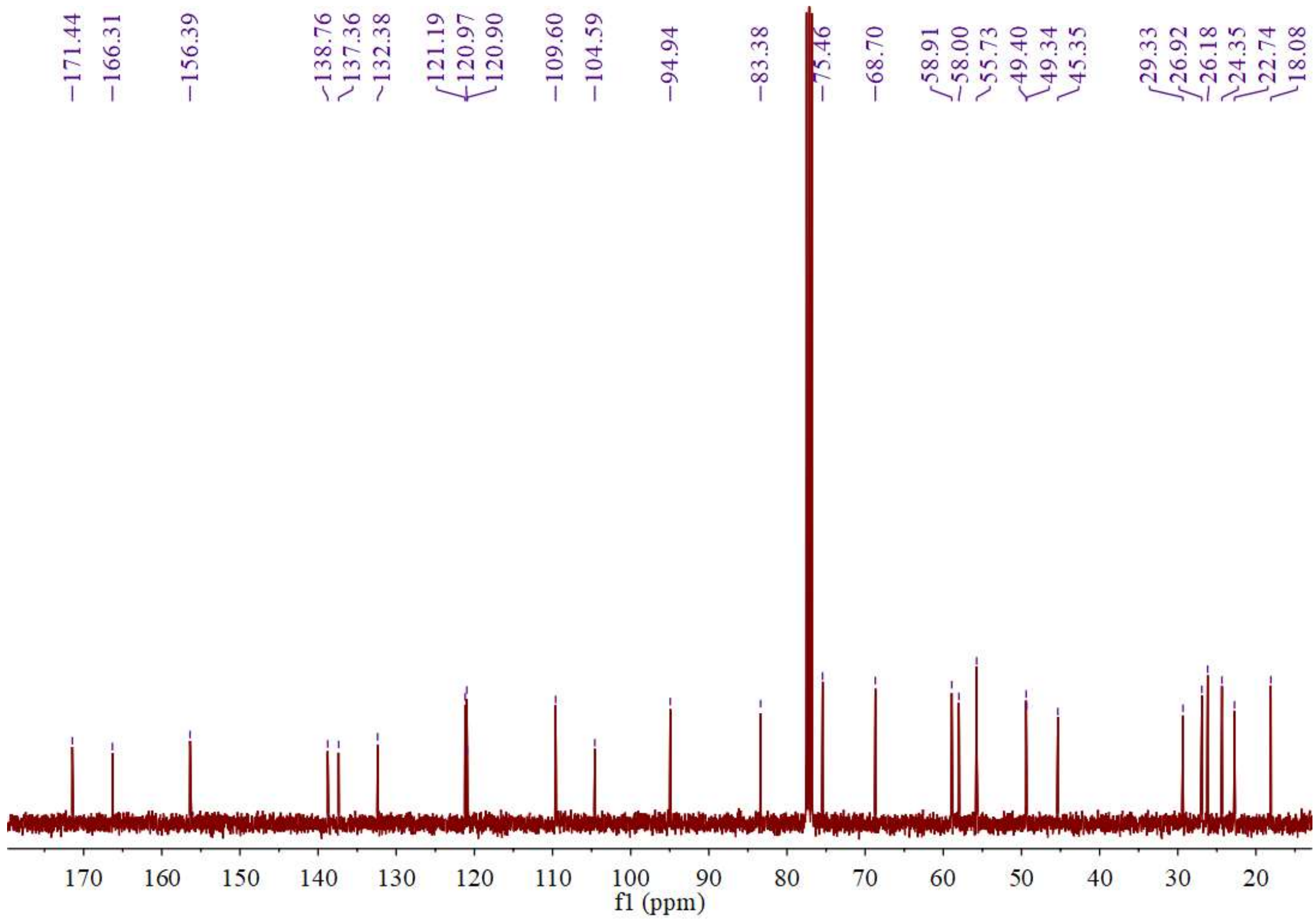

Fig. S9 ^{13}C NMR (100 MHz, $CDCl_3$) spectrum of compound **2**.

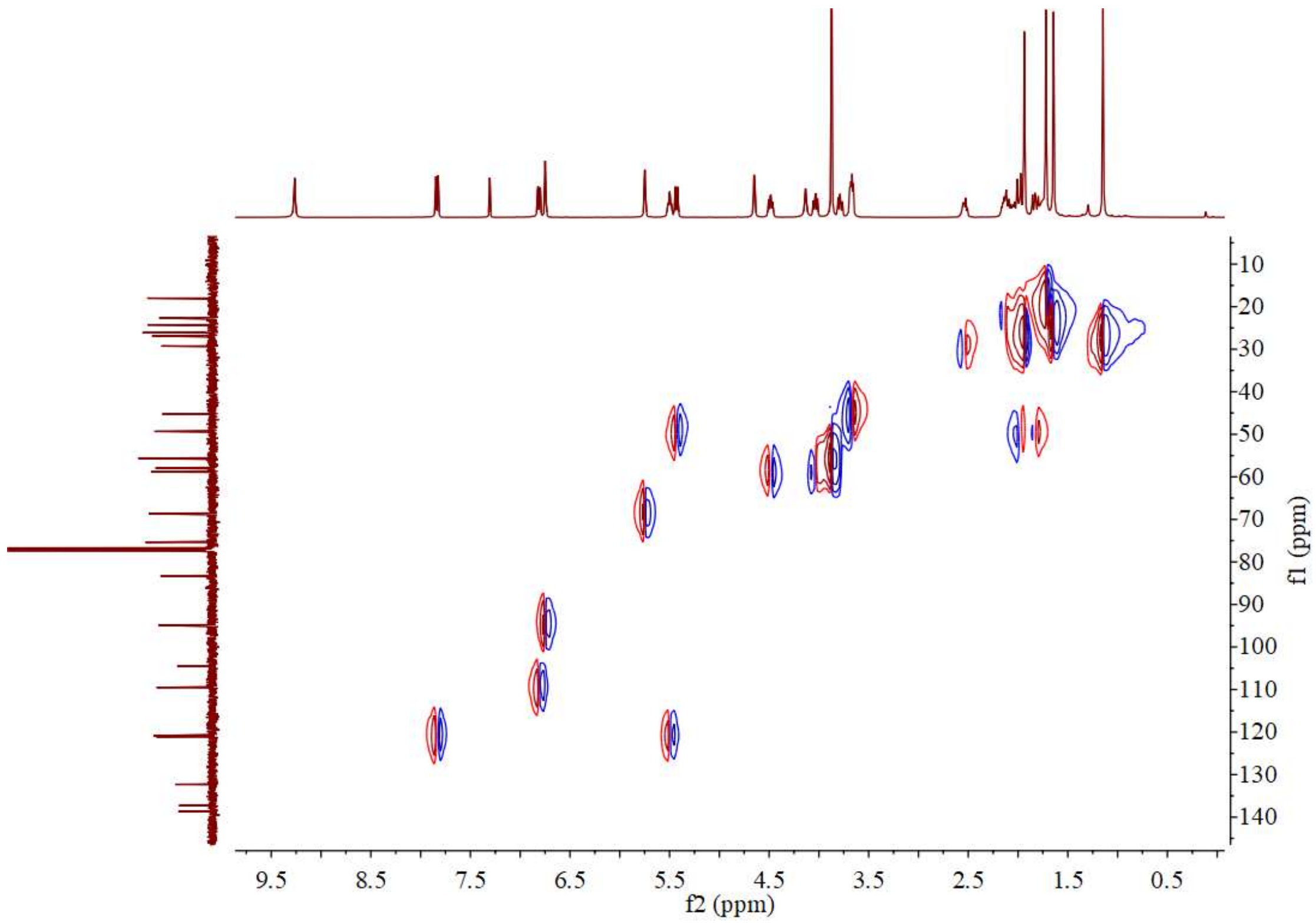

Fig. S10 HSQC ($CDCl_3$) spectrum of compound **2**.

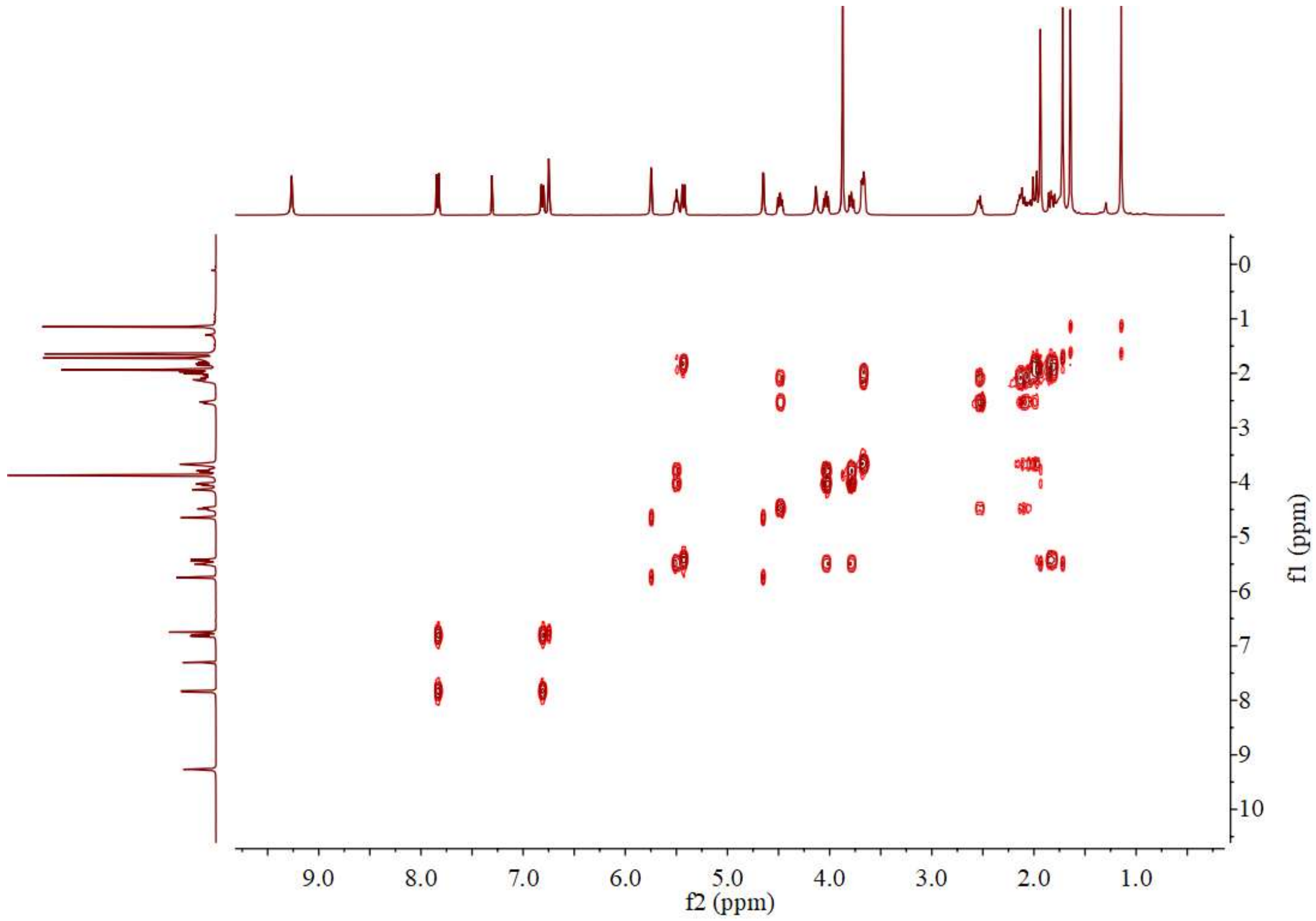

Fig. S11 ^{1}H–^{1}H COSY ($CDCl_3$) spectrum of compound **2**.

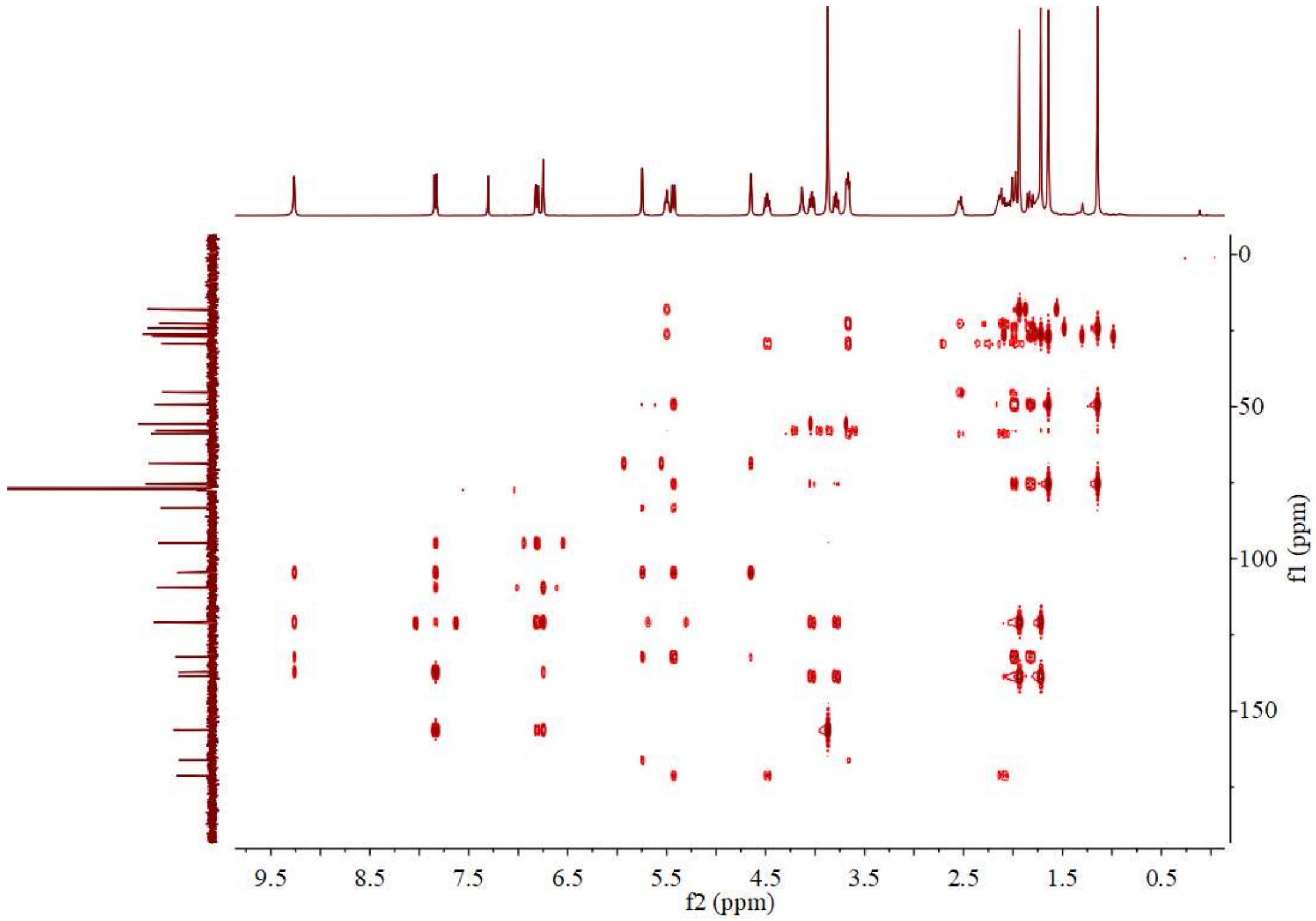

Fig. S12 HMBC ($CDCl_3$) spectrum of compound **2**.

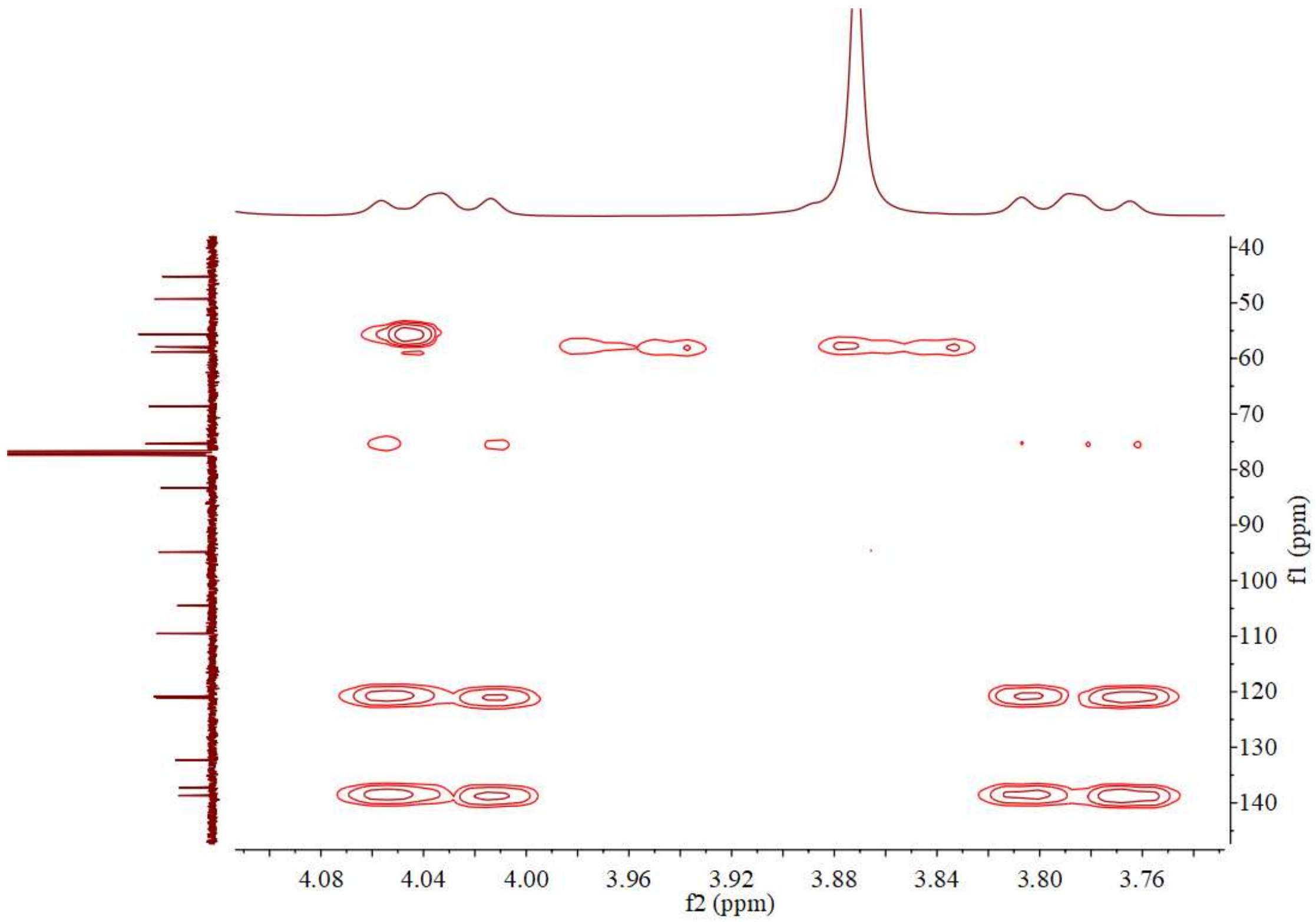

Fig. S13 Partial HMBC ($CDCl_3$) spectrum of compound **2**.

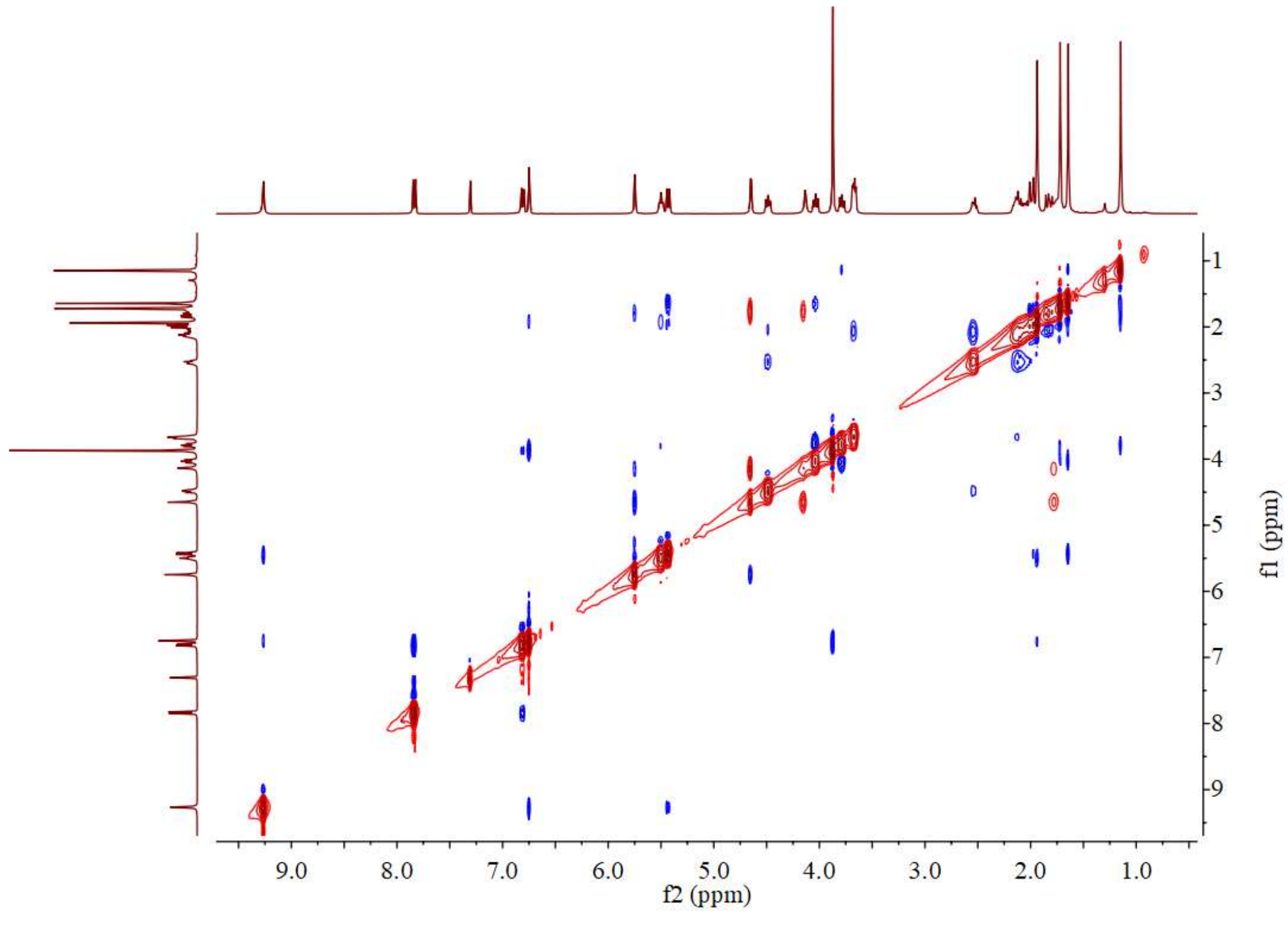

Fig. S14 NOESY ($CDCl_3$) spectrum of compound **2**.

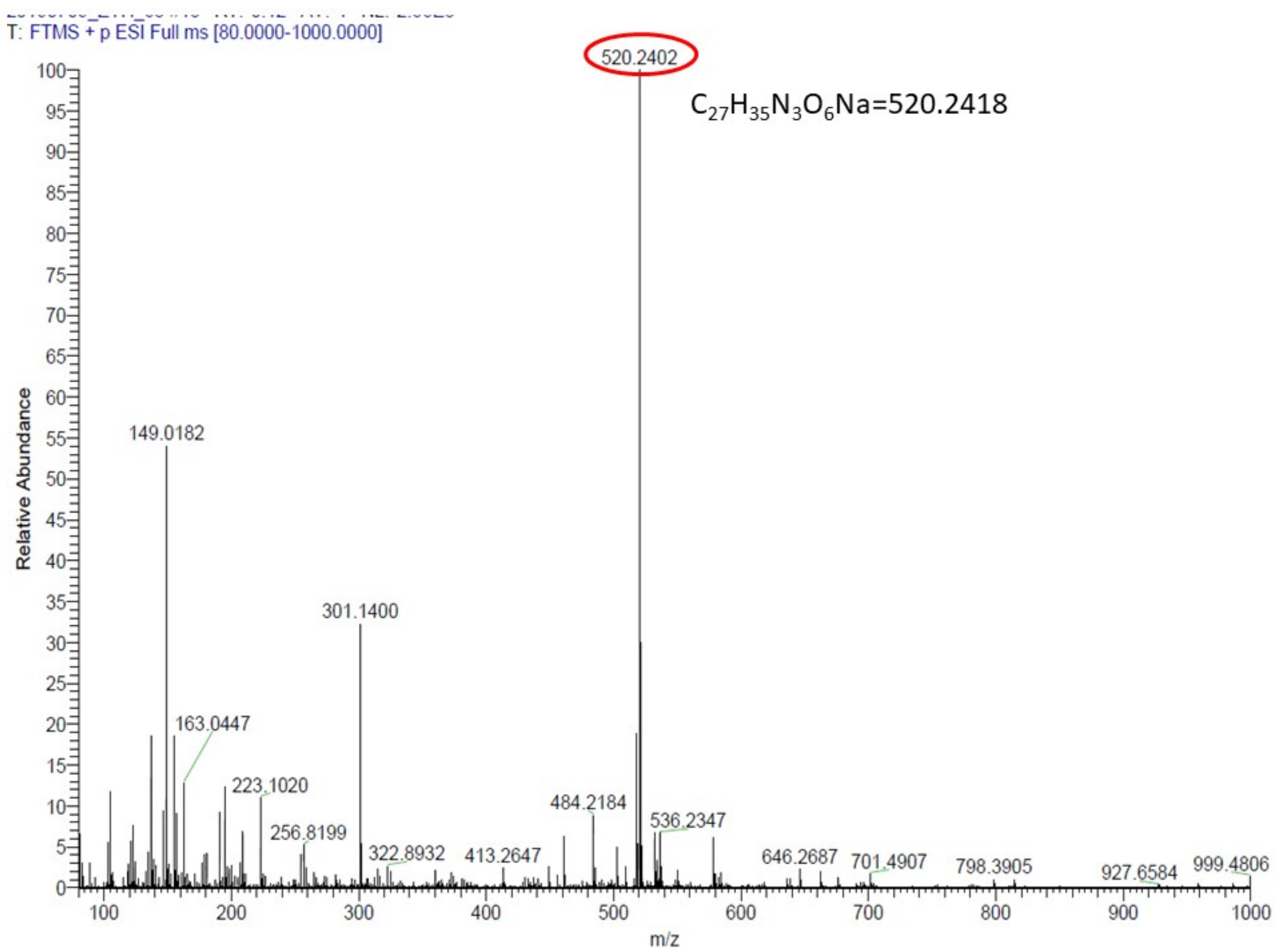

Fig. S15 HRESIMS spectrum of compound **2**.

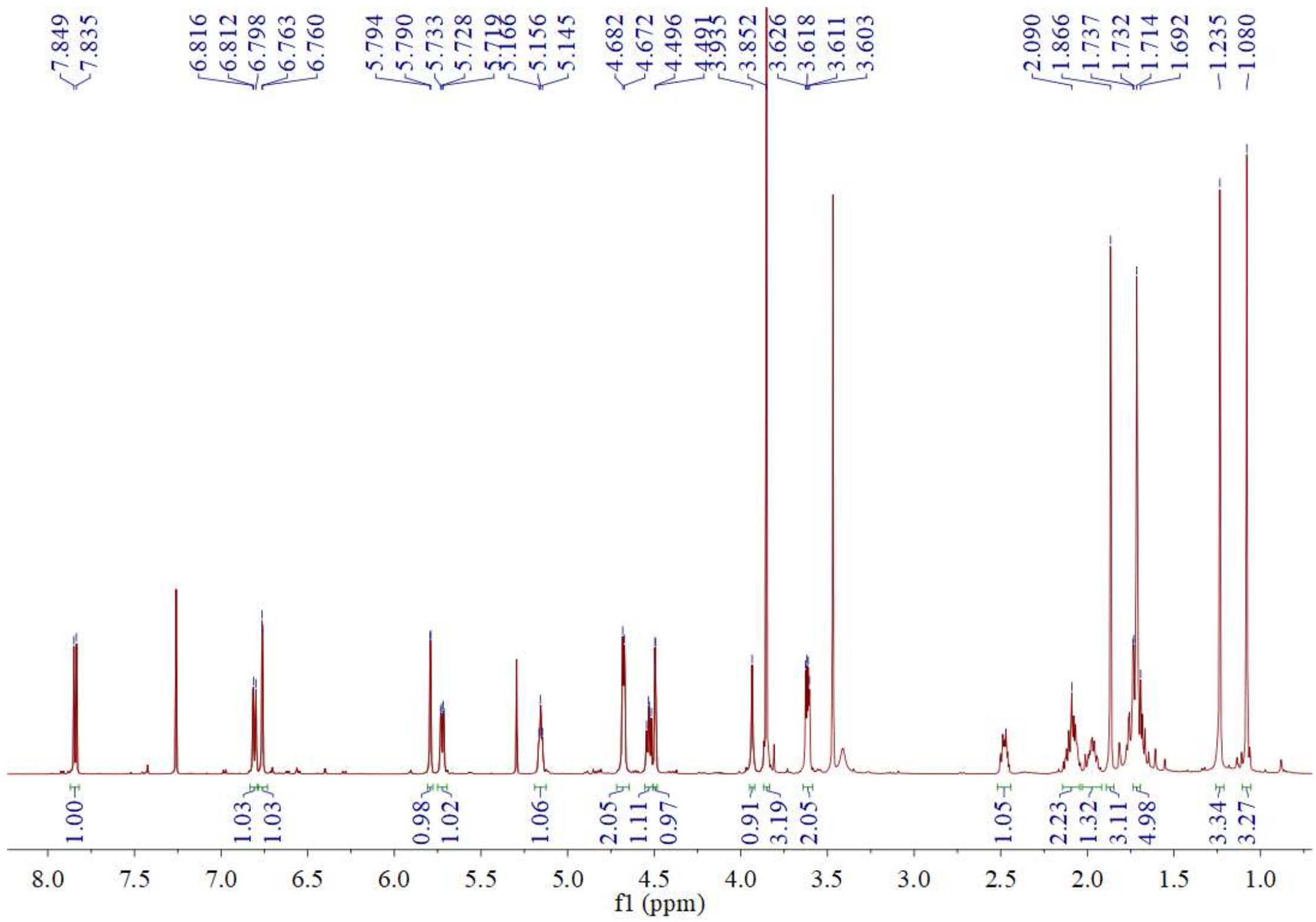

Fig. S16 ^{1}H NMR (600 MHz, $CDCl_3$) spectrum of compound **3**.

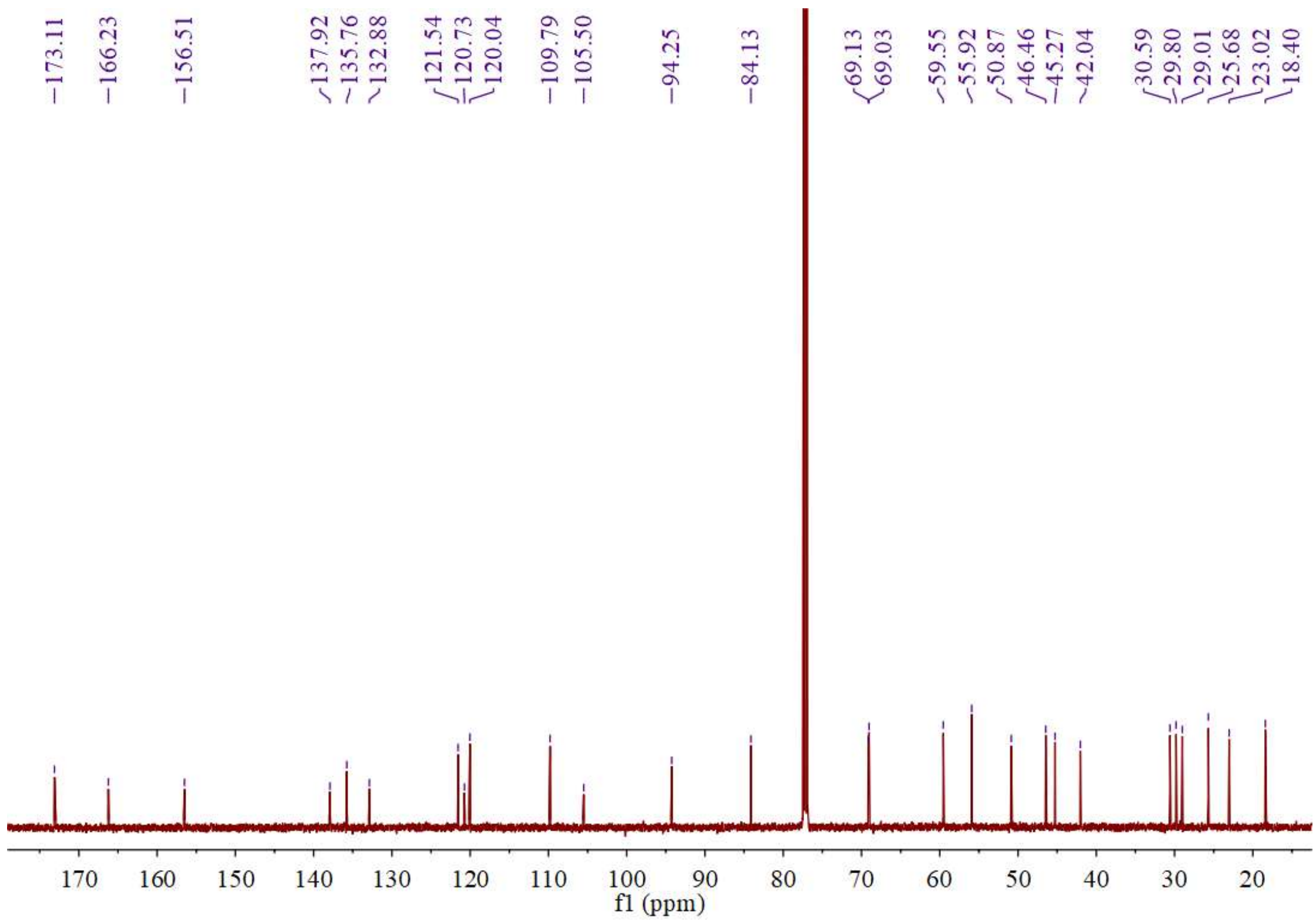

Fig. S17 ^{13}C NMR (150 MHz, $CDCl_3$) spectrum of compound **3**.

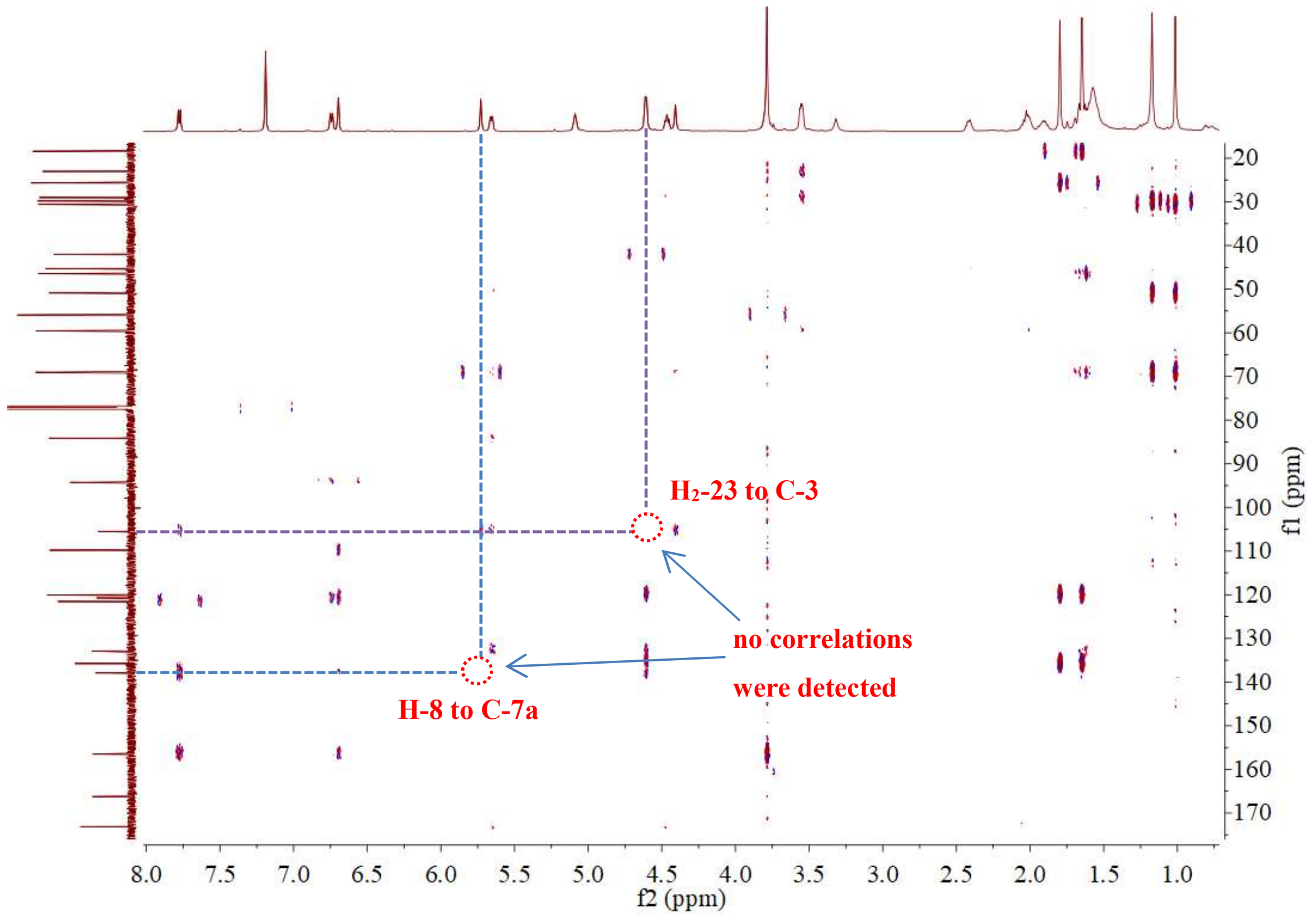

Fig. S18 HMBC ($CDCl_3$) spectrum of compound **3**.

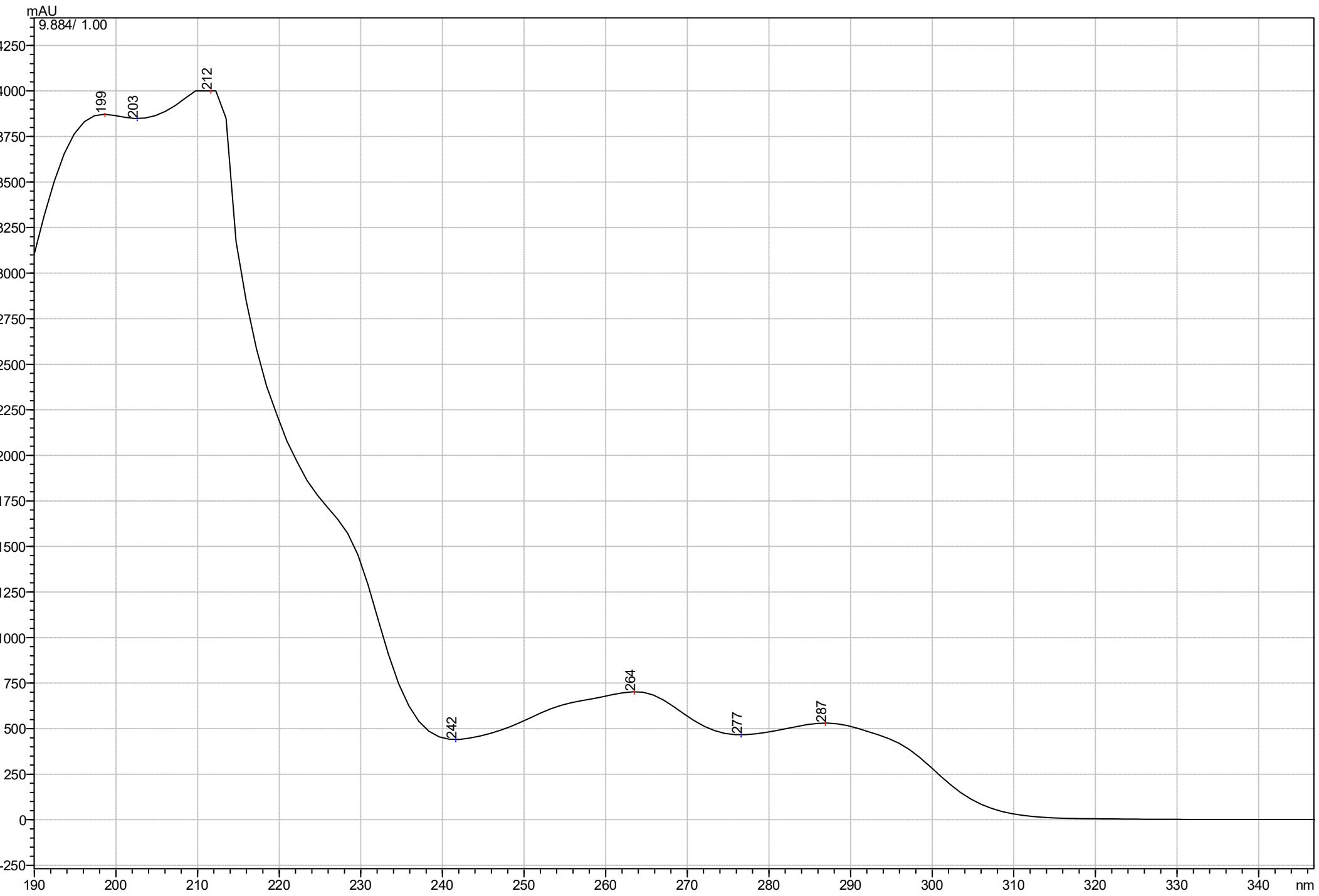

Fig. S19 Experimental UV spectrum of **1**.

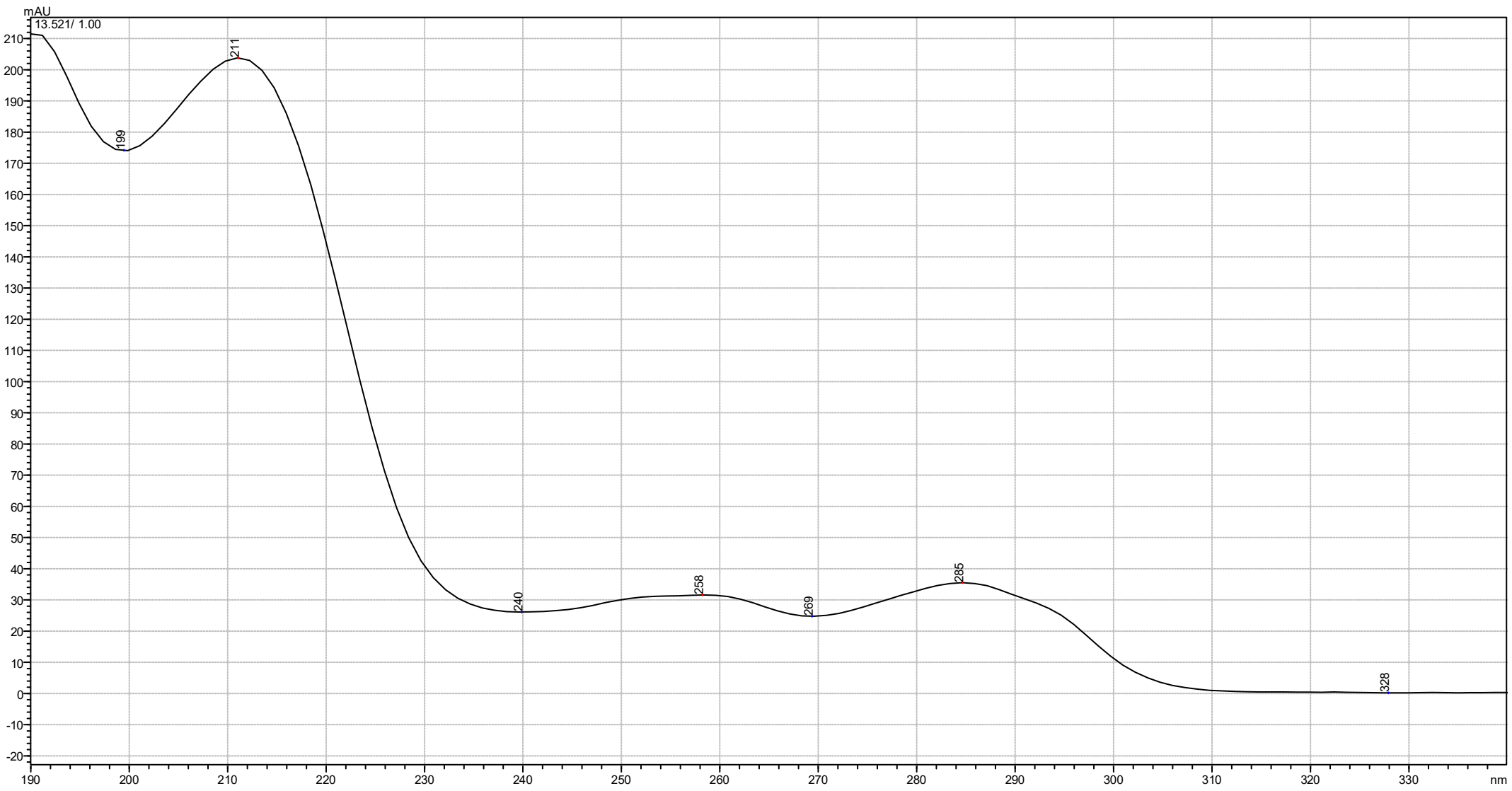

Fig. S20 Experimental UV spectrum of **2**.

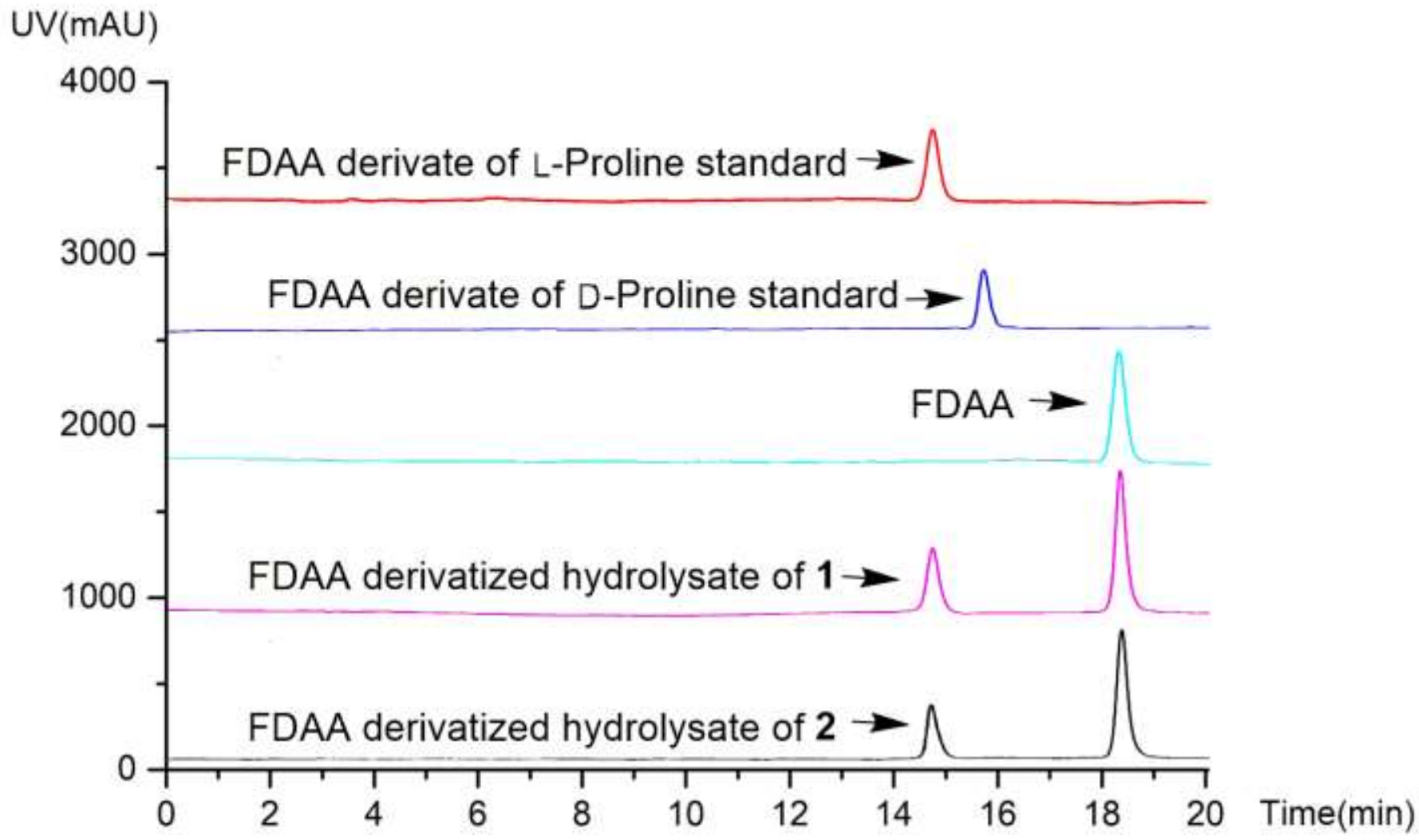

Fig. S21 HPLC at 254 nm of the Marfey's analysis (FDAA derivate of L-Proline standard t_R 14.5 min; FDAA derivate of D-Proline standard t_R 15.5 min; MeCN-H_2O (35:65, *v*:*v*), *v* = 2.0 mL/min).

Table S1 The coordinate for the lowest-energy conformer of compound **1** in ^{13}C NMR, ECD, and ORD calculations

Standard orientation:

Center Number	Atomic Number	Atomic Type	Coordinates (Angstroms) X	Y	Z
1	6	0	-4.085536	2.717589	0.373419
2	6	0	-4.868682	1.556209	0.188659
3	6	0	-4.285008	0.305788	0.116656
4	6	0	-2.889102	0.185321	0.229235
5	6	0	-2.130252	1.362427	0.416339
6	6	0	-2.700490	2.638568	0.489242
7	6	0	-1.972016	-0.933124	0.196671
8	6	0	-0.716639	-0.409388	0.362602
9	7	0	-0.799498	0.982794	0.496991
10	6	0	0.364072	1.816893	0.485719
11	6	0	1.368590	1.192157	-0.518765
12	6	0	2.690827	1.994814	-0.508653
13	7	0	1.644355	-0.218986	-0.208686
14	7	0	3.672179	1.394514	-1.190337
15	6	0	3.532667	0.046253	-1.760820
16	6	0	2.621624	-0.851718	-0.926658
17	8	0	2.785834	-2.065990	-0.929624
18	6	0	4.986164	-0.437231	-1.865766
19	6	0	5.761866	0.862231	-2.138392
20	6	0	5.046585	1.913981	-1.276584
21	8	0	2.796953	3.066759	0.082057
22	1	0	3.079888	0.113426	-2.757962
23	6	0	0.631133	-1.051871	0.517898
24	6	0	-2.343767	-2.385319	0.044204
25	6	0	-2.936083	-2.708241	-1.308736
26	6	0	-4.070716	-3.361074	-1.589250
27	6	0	-4.486289	-3.603552	-3.019785
28	6	0	-5.027198	-3.915328	-0.564058
29	8	0	0.887363	1.955528	1.788992
30	8	0	0.852281	1.344325	-1.837836
31	6	0	0.935694	-1.299276	2.018864
32	6	0	2.124571	-2.223705	2.403972
33	8	0	2.169777	-3.380049	1.560327
34	6	0	3.475561	-1.489439	2.397394
35	6	0	1.862392	-2.769851	3.810736
36	8	0	-4.792993	3.886091	0.433868
37	6	0	-4.084225	5.099371	0.620703

38	1	0	-5.942768	1.678402	0.106799
39	1	0	-4.903899	-0.571997	-0.033062
40	1	0	-2.088796	3.515073	0.657501
41	1	0	0.098414	2.793103	0.072770
42	1	0	5.109171	-1.191483	-2.642017
43	1	0	5.297330	-0.886623	-0.919154
44	1	0	6.821424	0.780836	-1.891067
45	1	0	5.690479	1.131731	-3.196404
46	1	0	5.046898	2.914761	-1.711382
47	1	0	5.470687	1.988391	-0.270440
48	1	0	0.618926	-2.014332	0.007243
49	1	0	-1.449088	-3.001979	0.193141
50	1	0	-3.029191	-2.668745	0.848288
51	1	0	-2.334293	-2.361981	-2.148881
52	1	0	-5.463576	-3.152183	-3.229415
53	1	0	-3.766414	-3.193296	-3.731075
54	1	0	-4.590335	-4.675698	-3.225328
55	1	0	-4.743297	-3.694667	0.464798
56	1	0	-6.038344	-3.522690	-0.724819
57	1	0	-5.104003	-5.005090	-0.657514
58	1	0	1.599128	2.613516	1.726715
59	1	0	-0.033491	0.952783	-1.872355
60	1	0	1.026631	-0.337986	2.527723
61	1	0	0.026480	-1.772151	2.402663
62	1	0	2.455247	-3.117825	0.669726
63	1	0	3.731003	-1.092766	1.414205
64	1	0	4.269141	-2.180342	2.692761
65	1	0	3.468934	-0.652179	3.101154
66	1	0	0.961350	-3.387584	3.813671
67	1	0	2.699115	-3.392202	4.136447
68	1	0	1.730034	-1.959373	4.532890
69	1	0	-3.539656	5.103536	1.571178
70	1	0	-3.383848	5.285248	-0.201098
71	1	0	-4.836712	5.885881	0.635156

Table S2 The coordinate for the lowest-energy conformer of compound **2** for ECD calculation

Standard orientation:

Center Number	Atomic Number	Atomic Type	Coordinates (Angstroms) X	Y	Z
1	6	0	-5.155766	-0.155648	-1.965038
2	6	0	-5.855548	0.007458	-0.748145
3	6	0	-5.186694	-0.003350	0.471868
4	6	0	-3.797591	-0.179202	0.434018
5	6	0	-3.074765	-0.349135	-0.772925
6	6	0	-3.786051	-0.335082	-1.985814
7	7	0	-2.888130	-0.222643	1.476028
8	6	0	-1.621304	-0.435431	0.957725
9	6	0	-1.694350	-0.511936	-0.399287
10	6	0	-0.449607	-0.676276	-1.195923
11	6	0	0.562332	-1.548970	-0.393104
12	6	0	1.928889	-1.510912	-1.127800
13	7	0	0.716979	-1.127209	0.997806
14	7	0	2.959185	-1.960461	-0.406826
15	6	0	2.821563	-2.409288	0.980151
16	6	0	1.727933	-1.676959	1.745422
17	8	0	1.771264	-1.617617	2.965234
18	6	0	4.237314	-2.221353	1.540326
19	6	0	5.124455	-2.545808	0.326075
20	6	0	4.352972	-1.971776	-0.875749
21	8	0	2.021845	-1.130599	-2.298152
22	1	0	2.549068	-3.473708	0.996059
23	6	0	-0.380522	-0.492017	1.780778
24	8	0	-7.208909	0.170957	-0.879745
25	6	0	-7.988085	0.338436	0.290294
26	8	0	-0.733048	-1.346219	-2.423317
27	8	0	0.114677	-2.906580	-0.402309
28	6	0	0.046736	0.854305	2.435984
29	6	0	0.260807	2.153114	1.622853
30	8	0	1.192590	1.810041	0.573130
31	6	0	-1.046117	2.728909	1.051408
32	6	0	0.885888	3.175289	2.593104
33	6	0	1.643638	2.869668	-0.280423
34	6	0	2.628633	2.302630	-1.256245
35	6	0	3.854910	2.759268	-1.542423
36	6	0	4.688897	2.087940	-2.606651
37	6	0	4.516117	3.950197	-0.895425

38	1	0	-5.730976	-0.141218	-2.883931
39	1	0	-5.705362	0.118973	1.414671
40	1	0	-3.262264	-0.473856	-2.923768
41	1	0	-3.125181	-0.222589	2.454381
42	1	0	0.023047	0.287522	-1.406345
43	1	0	4.371766	-1.183902	1.858376
44	1	0	4.425106	-2.859680	2.402801
45	1	0	5.235072	-3.629216	0.219523
46	1	0	6.126763	-2.122139	0.409420
47	1	0	4.657947	-0.950646	-1.120956
48	1	0	4.448147	-2.572900	-1.782428
49	1	0	-0.549485	-1.159869	2.634167
50	1	0	-9.017301	0.447897	-0.047689
51	1	0	-7.916703	-0.534102	0.949682
52	1	0	-7.693732	1.237167	0.844339
53	1	0	0.082014	-1.278450	-2.948436
54	1	0	-0.415667	-3.018288	-1.209651
55	1	0	-0.710999	1.075916	3.196964
56	1	0	0.962283	0.642306	2.989395
57	1	0	-0.890868	3.726898	0.634397
58	1	0	-1.794579	2.828714	1.841953
59	1	0	-1.464683	2.094755	0.270691
60	1	0	0.261633	3.286792	3.483828
61	1	0	1.877101	2.845701	2.914198
62	1	0	0.978137	4.166527	2.143638
63	1	0	0.788718	3.290401	-0.828705
64	1	0	2.082327	3.678396	0.309003
65	1	0	2.269273	1.433660	-1.802390
66	1	0	4.181166	1.224230	-3.039177
67	1	0	4.925365	2.785300	-3.419558
68	1	0	5.651926	1.754301	-2.200569
69	1	0	3.954181	4.361520	-0.056937
70	1	0	5.514952	3.684516	-0.530867
71	1	0	4.660125	4.756952	-1.624194

Table S3 Cytotoxic activity data of compounds **1** and **2**

Compd.	IC_{50} (μM)			
	A549	HepG2	AGS	HGC-27
**1**	>30	>30	>30	>30
**2**	>30	>30	>30	>30
**DDP**	1.26	3.25	2.43	2.50
