# Supplementary material for: Novel anti-inflammatory diketopiperazine alkaloids from the marine-derived fungus Penicillium brasilianum
Source: Appl Microbiol Biotechnol. 2024 Feb 5;108(1):194. doi: 10.1007/s00253-024-13026-4 (PMC10844341; doi:10.1007/s00253-024-13026-4)
Supplement: Supplementary file 1 — Supplementary file1 (PDF 1424 KB) [file 253_2024_13026_MOESM1_ESM.pdf]

# Supporting Information

## Novel Anti-inflammatory Diketopiperazine Alkaloids from the Marine-Derived Fungus *Penicillium brasilianum*

Ya-Hui Zhang,[1] Hui-Fang Du,[2] Yun-Feng Liu,[1] Fei Cao,[2]* Du-Qiang Luo,[1]* and Chang-Yun Wang,[3]*

[1]College of Life Sciences, Key Laboratory of Medicinal Chemistry and Molecular Diagnostics of Education Ministry of China, Hebei University, Baoding 071002, China.

[2]College of Pharmaceutical Sciences, Key Laboratory of Pharmaceutical Quality Control of Hebei Province, Hebei University, Baoding 071002, China

[3]Laboratory for Marine Drugs and Bioproducts, Qingdao National Laboratory for Marine Science and Technology; Key Laboratory of Marine Drugs, the Ministry of Education of China, School of Medicine and Pharmacy, Institute of Evolution & Marine Biodiversity, Ocean University of China; Qingdao 266003, China

*Correspondence: F.C. (caofei542927001@163.com), D.Q.L. (duqiangluo@hbu.edu.cn) and C.Y.W. (changyun@ouc.edu.cn)

# List of Supporting Information

**Fig. S1** $^{1}$H NMR (400 MHz, $CDCl_3$) spectrum of compound **1**.

**Fig. S2** $^{13}$C NMR (100 MHz, $CDCl_3$) spectrum of compound **1**.

**Fig. S3** HSQC ($CDCl_3$) spectrum of compound **1**.

**Fig. S4** $^{1}$H–$^{1}$H COSY ($CDCl_3$) spectrum of compound **1**.

**Fig. S5** HMBC ($CDCl_3$) spectrum of compound **1**.

**Fig. S6** NOESY ($CDCl_3$) spectrum of compound **1**.

**Fig. S7** HRESIMS spectrum of compound **1**.

**Fig. S8** $^{1}$H NMR (400 MHz, $CDCl_3$) spectrum of compound **2**.

**Fig. S9** $^{13}$C NMR (100 MHz, $CDCl_3$) spectrum of compound **2**.

**Fig. S10** HSQC ($CDCl_3$) spectrum of compound **2**.

**Fig. S11** $^{1}$H–$^{1}$H COSY ($CDCl_3$) spectrum of compound **2**.

**Fig. S12** HMBC ($CDCl_3$) spectrum of compound **2**.

**Fig. S13** Partial HMBC ($CDCl_3$) spectrum of compound **2**.

**Fig. S14** NOESY ($CDCl_3$) spectrum of compound **2**.

**Fig. S15** HRESIMS spectrum of compound **2**.

**Fig. S16** $^{1}$H NMR (600 MHz, $CDCl_3$) spectrum of compound **3**.

**Fig. S17** $^{13}$C NMR (150 MHz, $CDCl_3$) spectrum of compound **3**.

**Fig. S18** HMBC ($CDCl_3$) spectrum of compound **3**.

**Fig. S19** Experimental UV spectrum of **1**.

**Fig. S20** Experimental UV spectrum of **2**.

**Fig. S21** HPLC at 254 nm of the Marfey's analysis.

**Table S1** The coordinate for the lowest-energy conformer of compound **1** in $^{13}$C NMR, ECD, and ORD calculations.

**Table S2** The coordinate for the lowest-energy conformer of compound **2** for ECD calculation.

**Table S3** Cytotoxic activity data of compounds **1** and **2**.

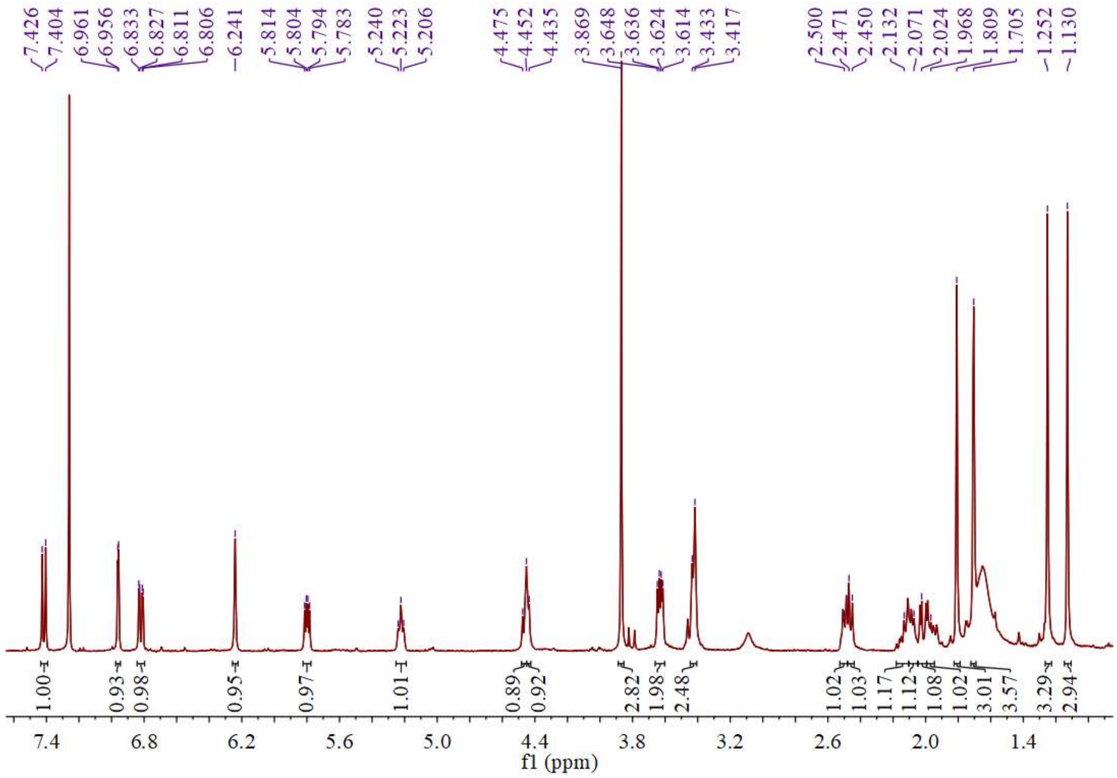


**Fig. S1** $^{1}$H NMR (400 MHz, $CDCl_3$) spectrum of compound **1**.

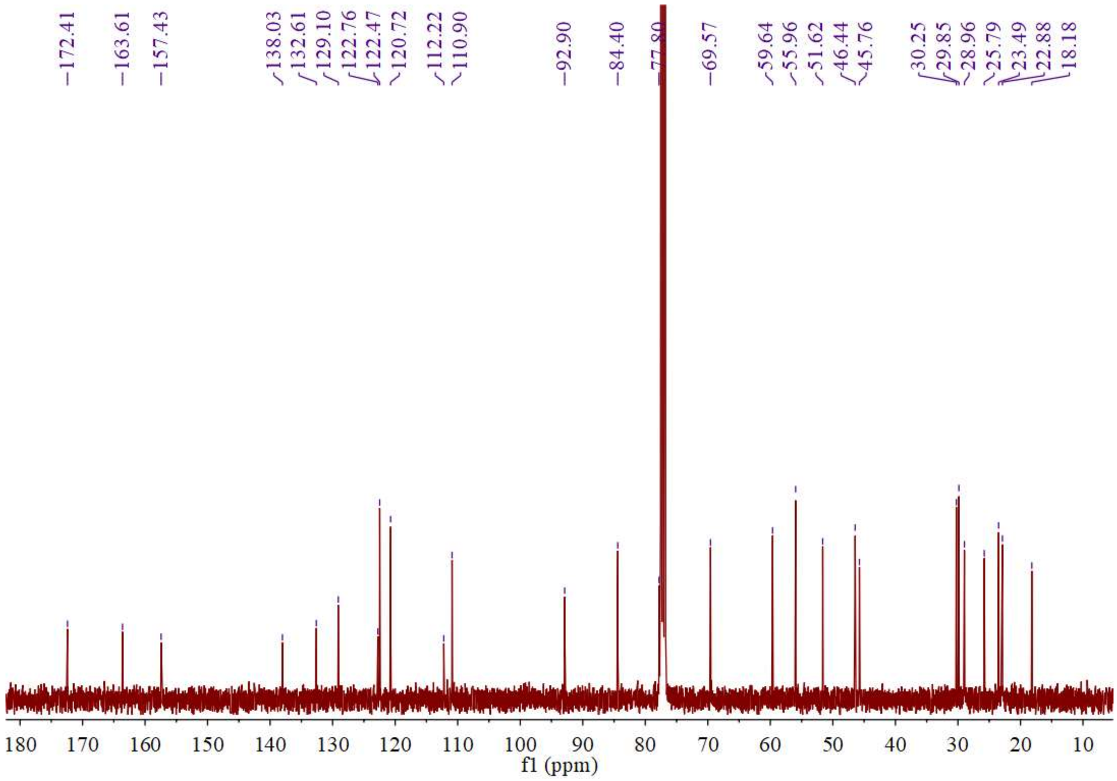


**Fig. S2** $^{13}$C NMR (100 MHz, $CDCl_3$) spectrum of compound **1**.

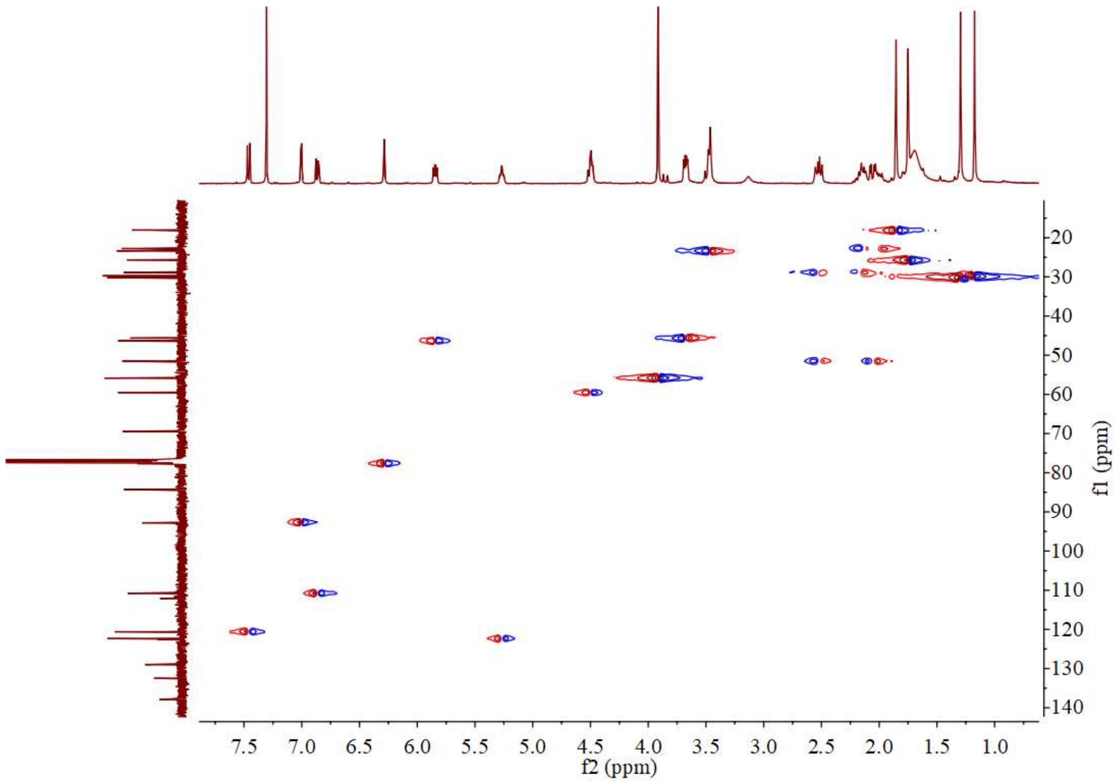


**Fig. S3** HSQC ($CDCl_3$) spectrum of compound **1**.

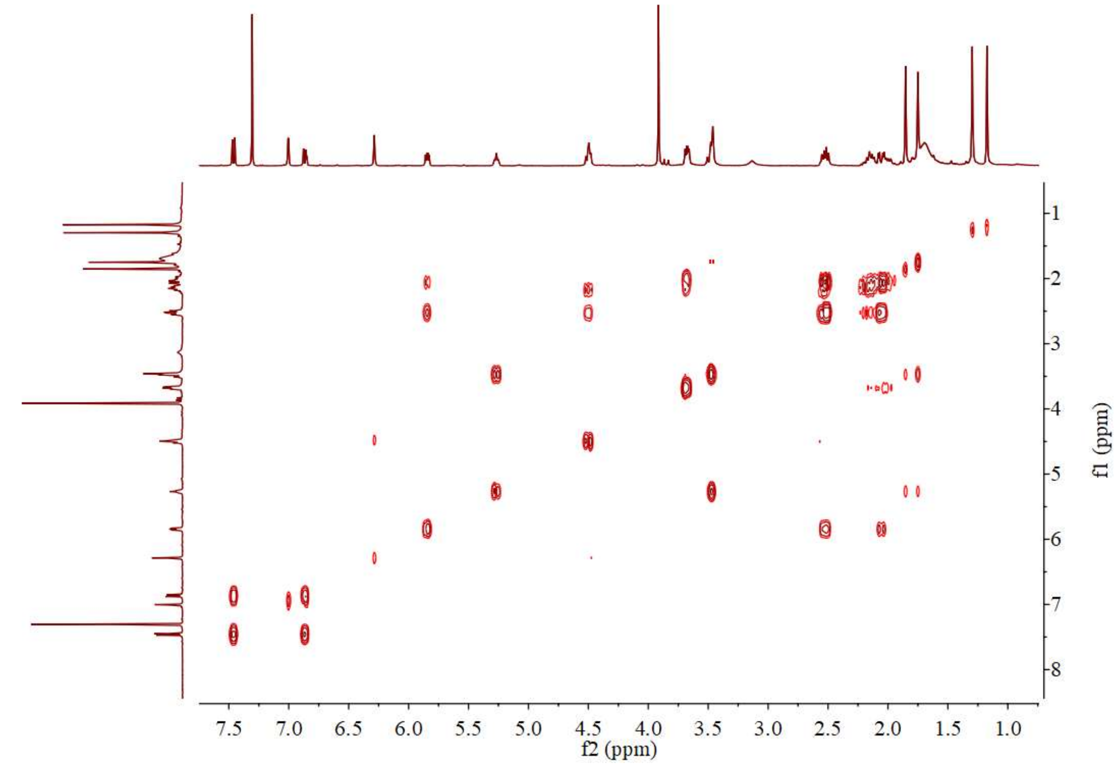


**Fig. S4** $^{1}$H–$^{1}$H COSY ($CDCl_3$) spectrum of compound **1**.

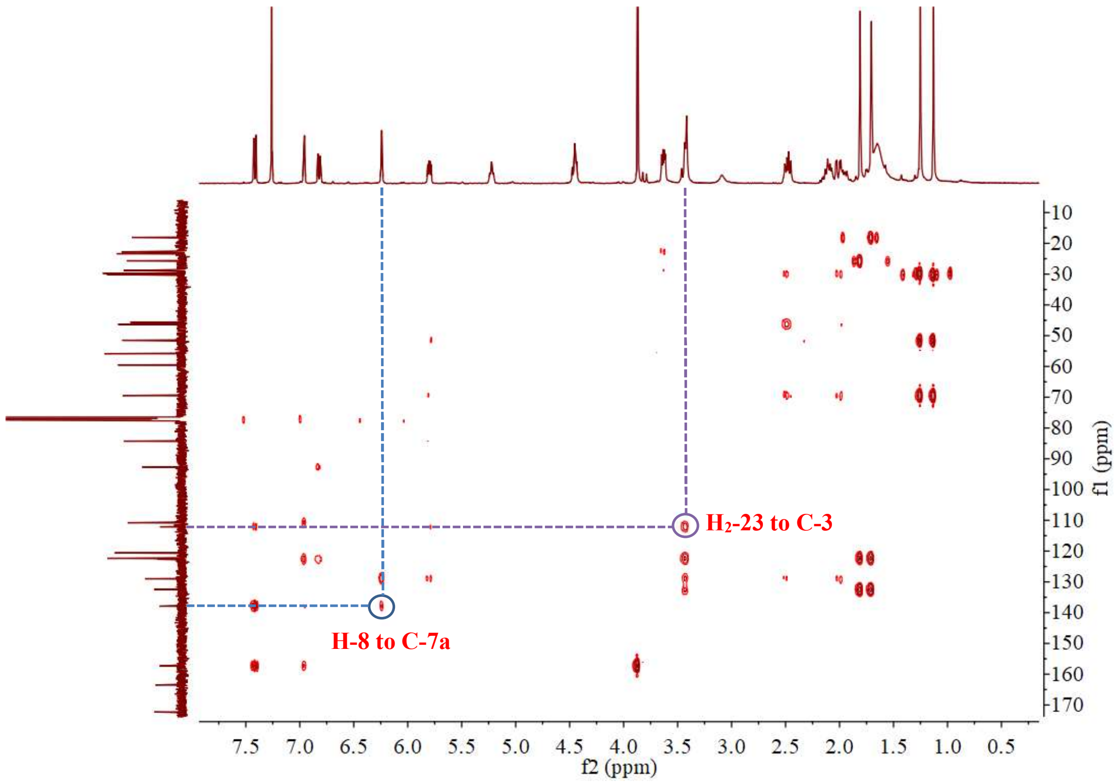


**Fig. S5** HMBC ($CDCl_3$) spectrum of compound **1**.

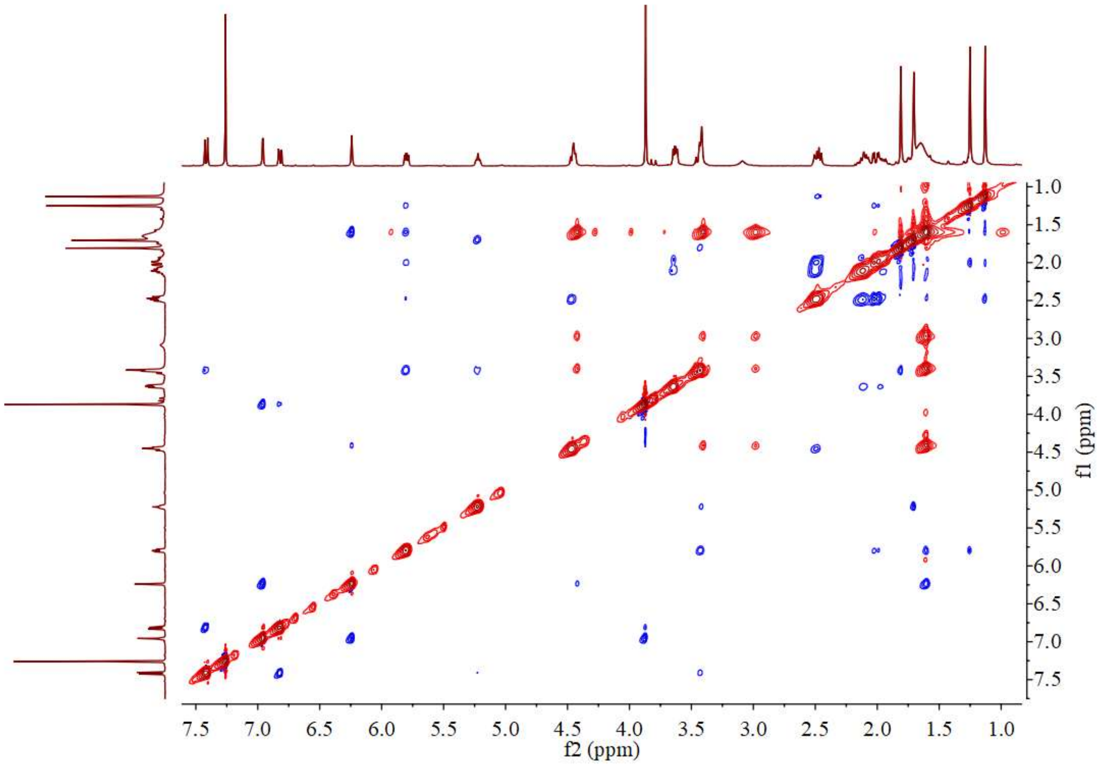


**Fig. S6** NOESY ($CDCl_3$) spectrum of compound **1**.

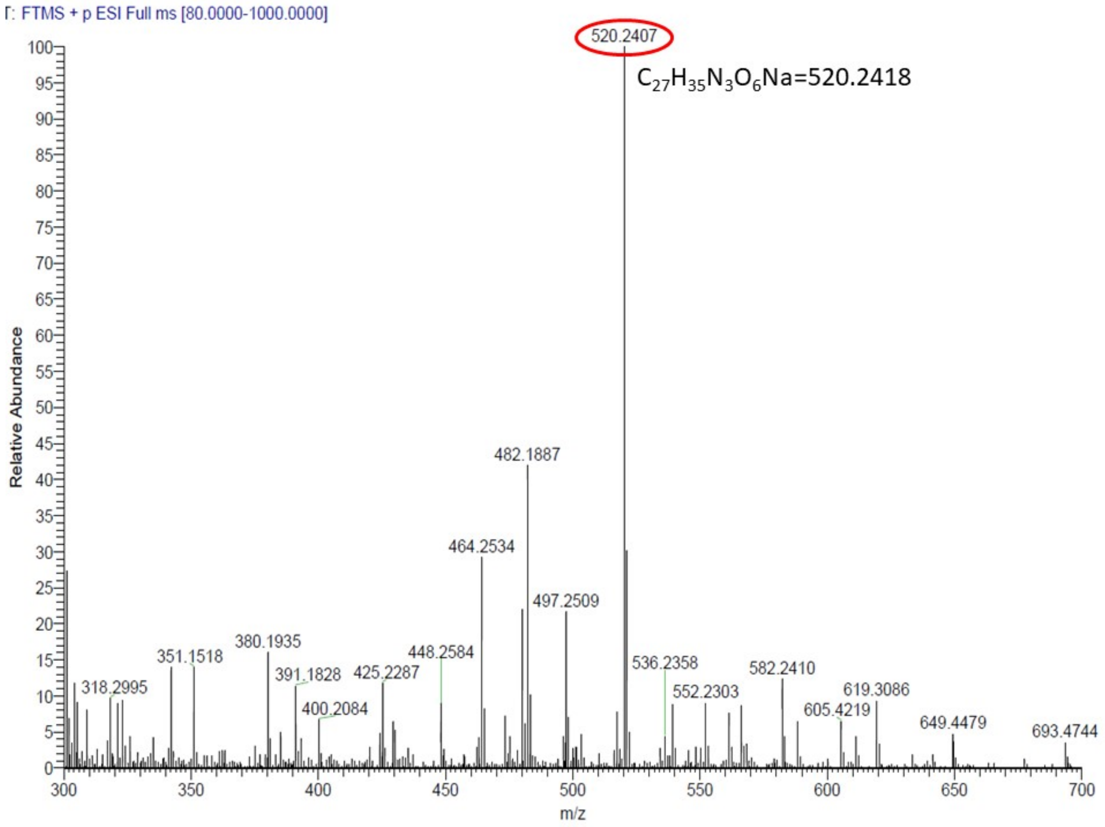


**Fig. S7** HRESIMS spectrum of compound **1**.

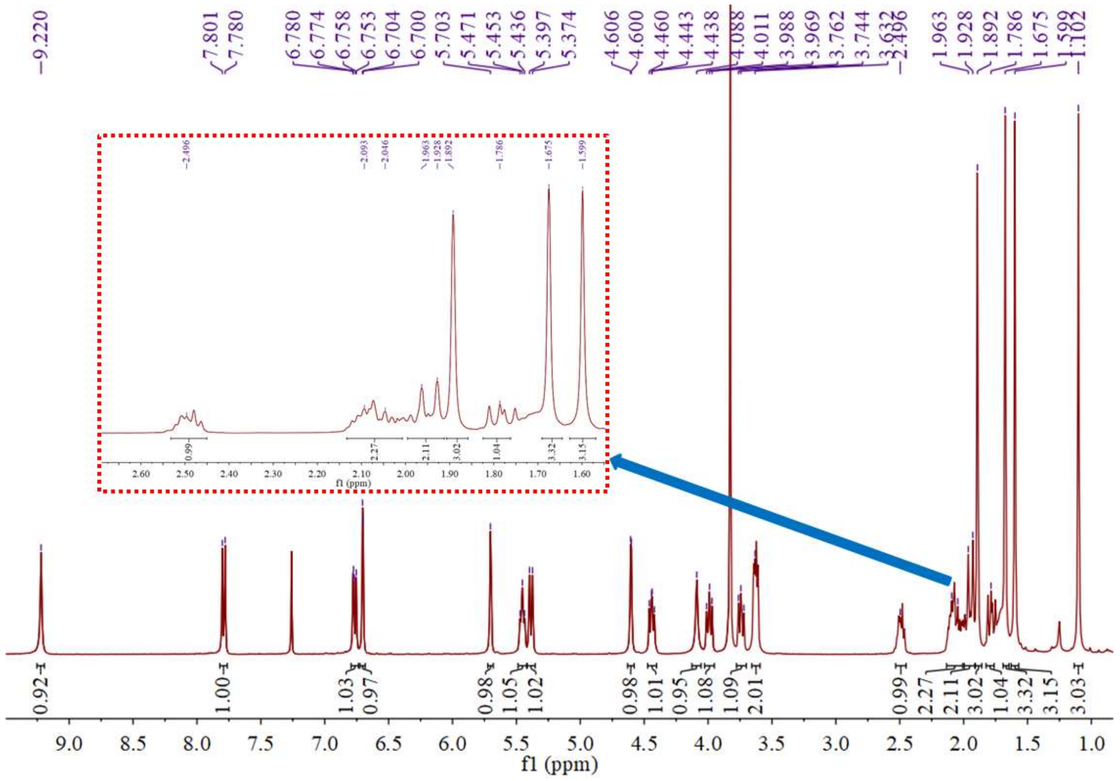


**Fig. S8** $^{1}$H NMR (400 MHz, $CDCl_3$) spectrum of compound **2**.

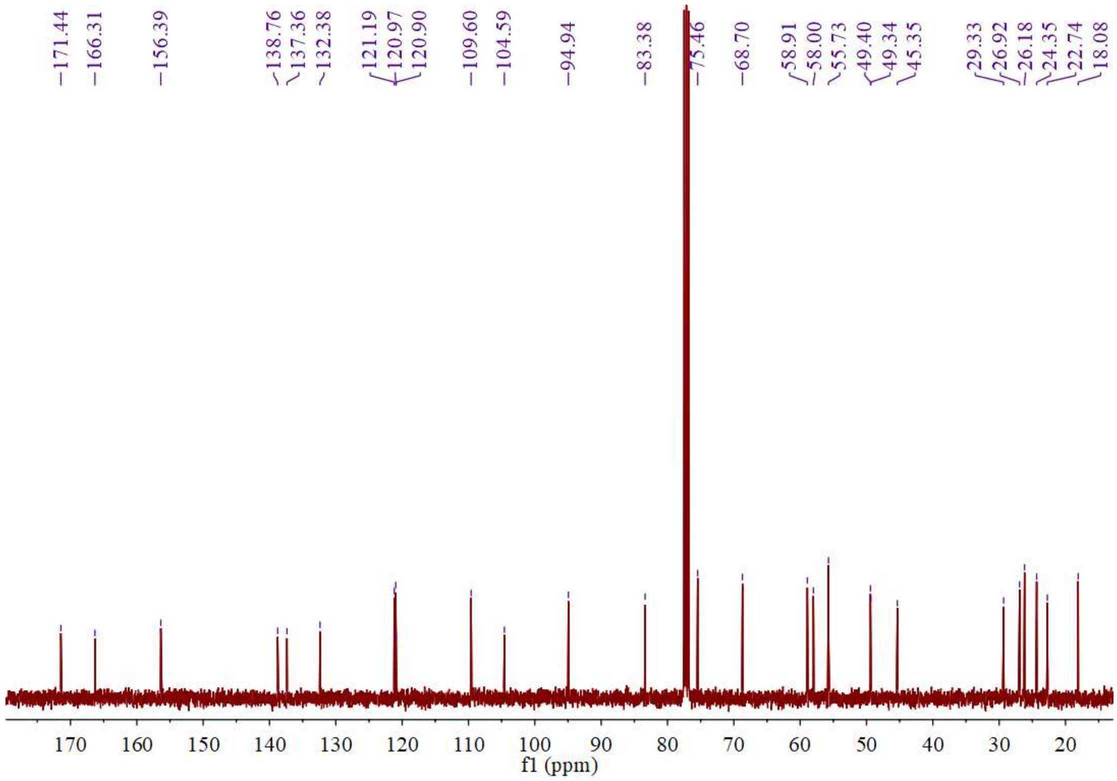


**Fig. S9** $^{13}$C NMR (100 MHz, $CDCl_3$) spectrum of compound **2**.

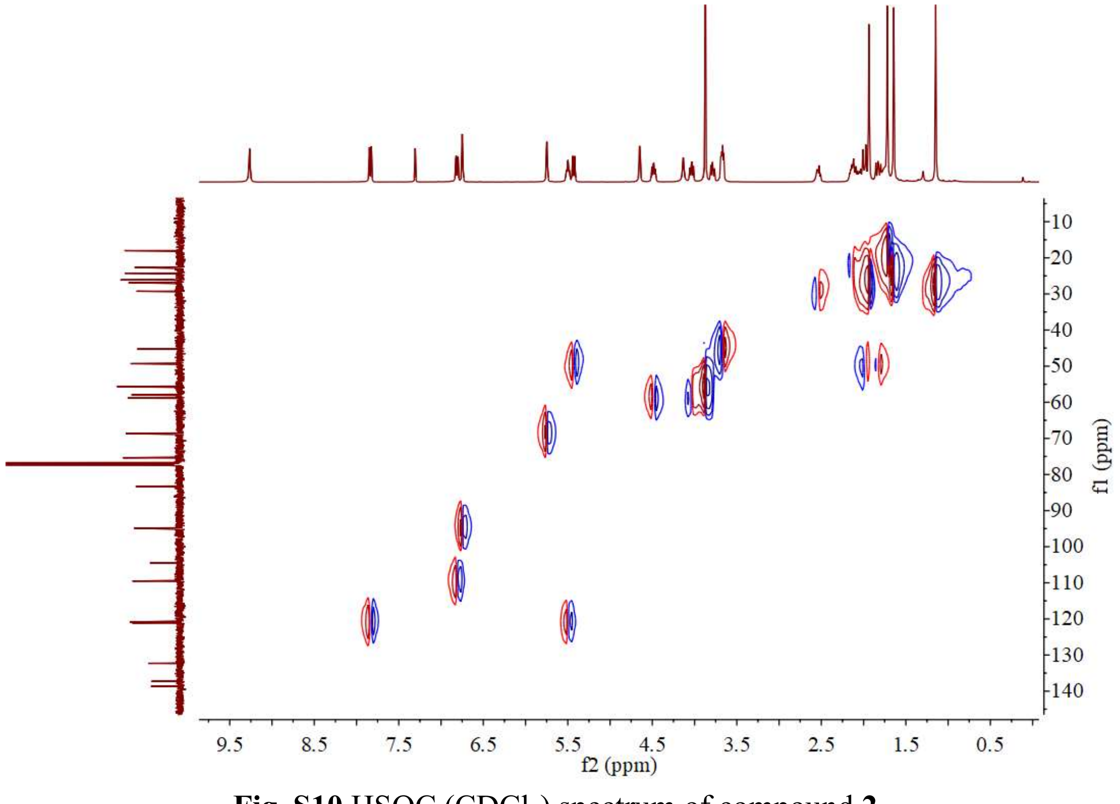


**Fig. S10** HSQC ($CDCl_3$) spectrum of compound **2**.

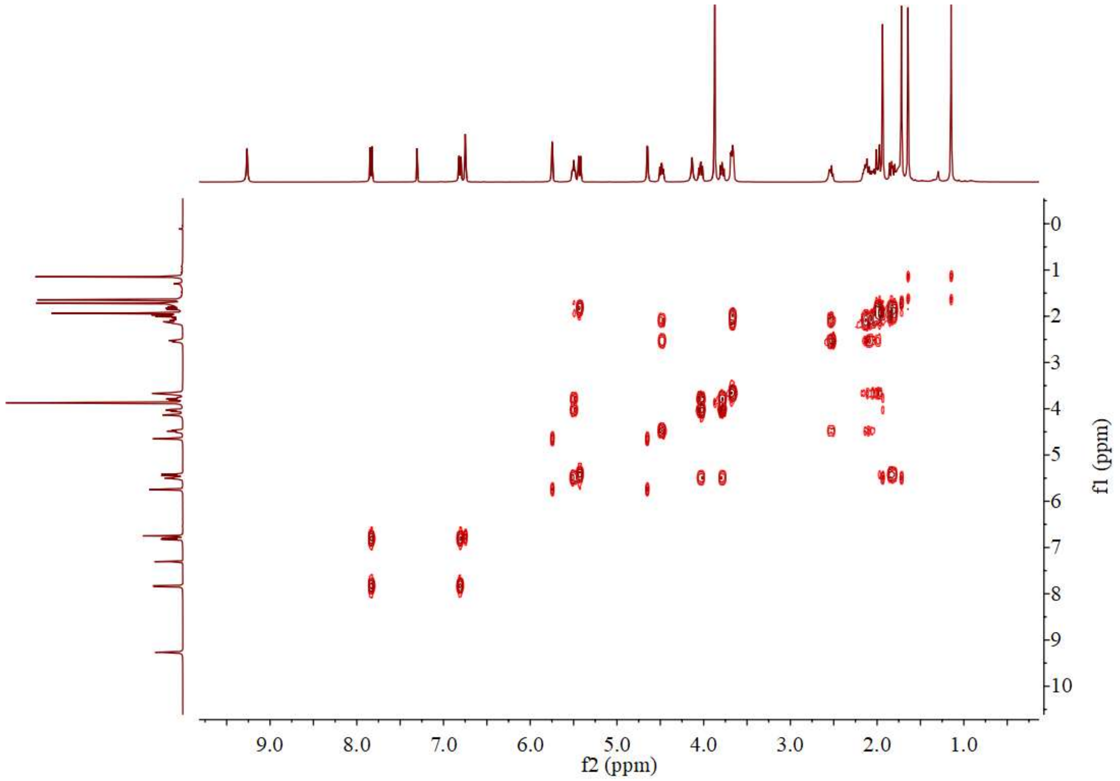


**Fig. S11** $^{1}H$–$^{1}H$ COSY ($CDCl_3$) spectrum of compound **2**.

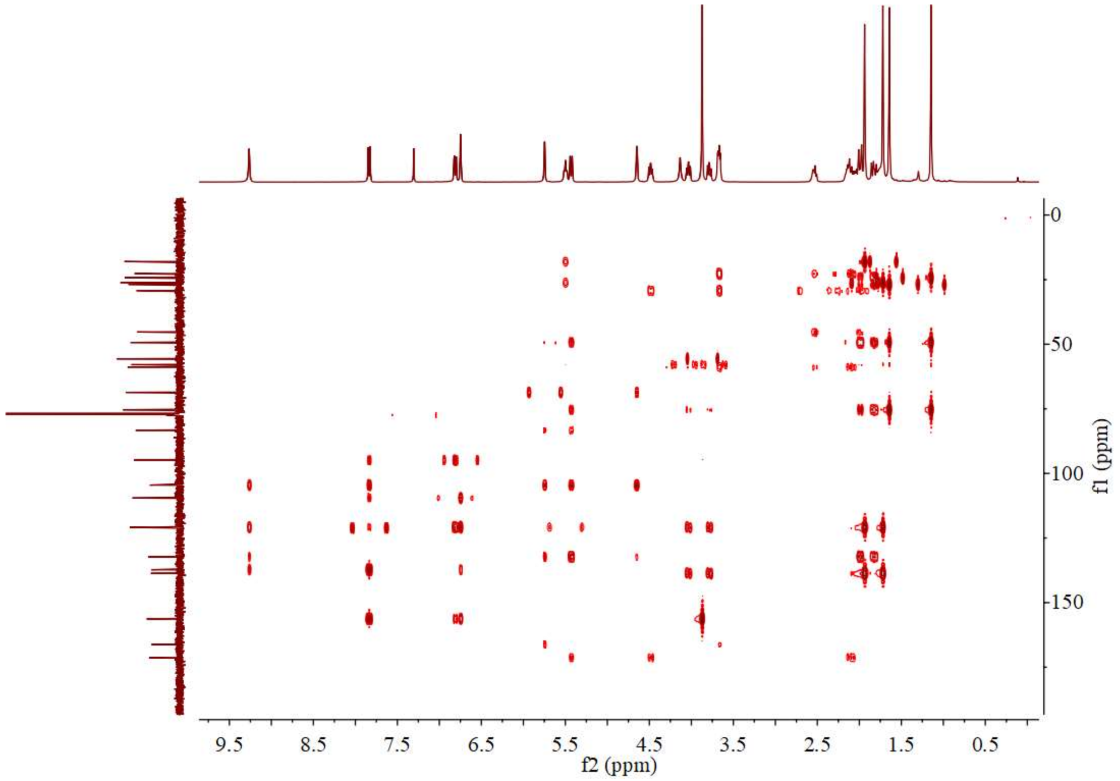


**Fig. S12** HMBC ($CDCl_3$) spectrum of compound **2**.

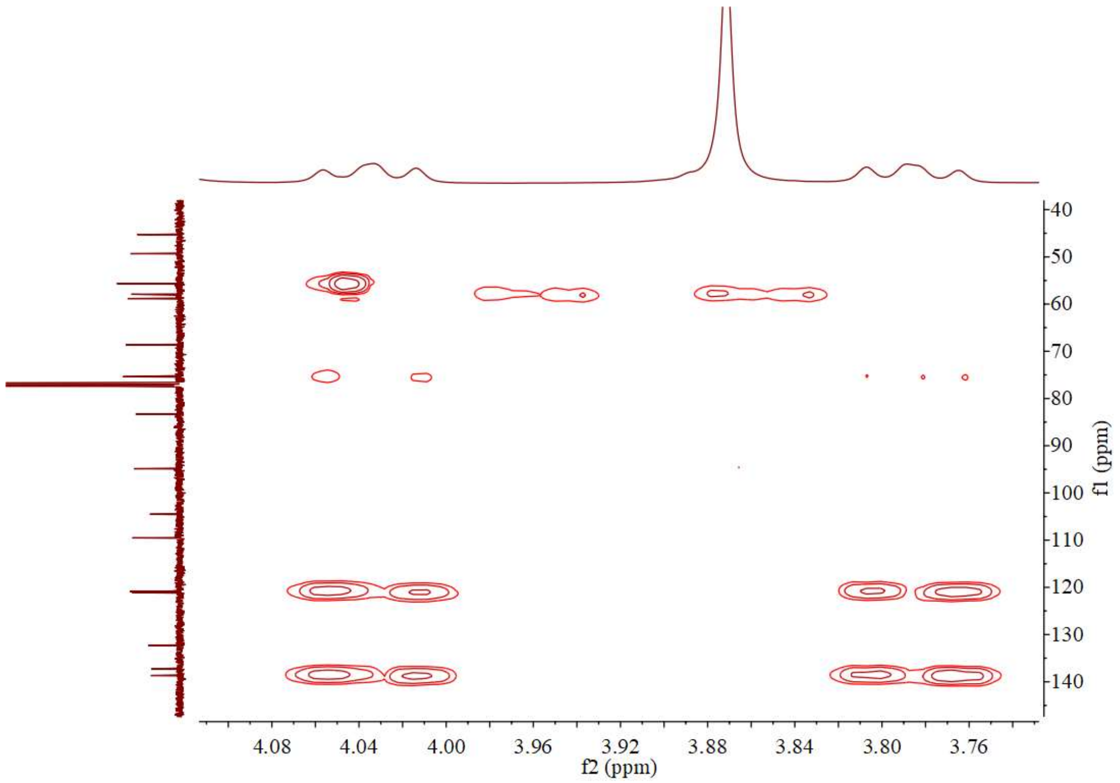


**Fig. S13** Partial HMBC ($CDCl_3$) spectrum of compound **2**.

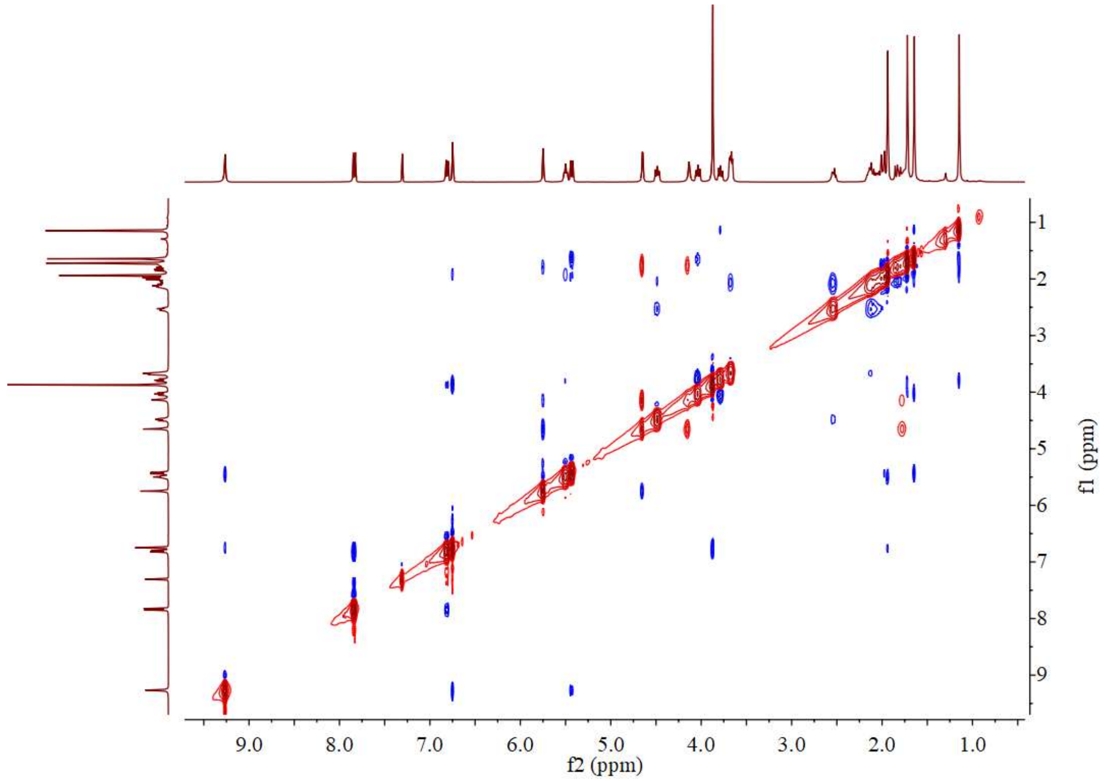


**Fig. S14** NOESY ($CDCl_3$) spectrum of compound **2**.

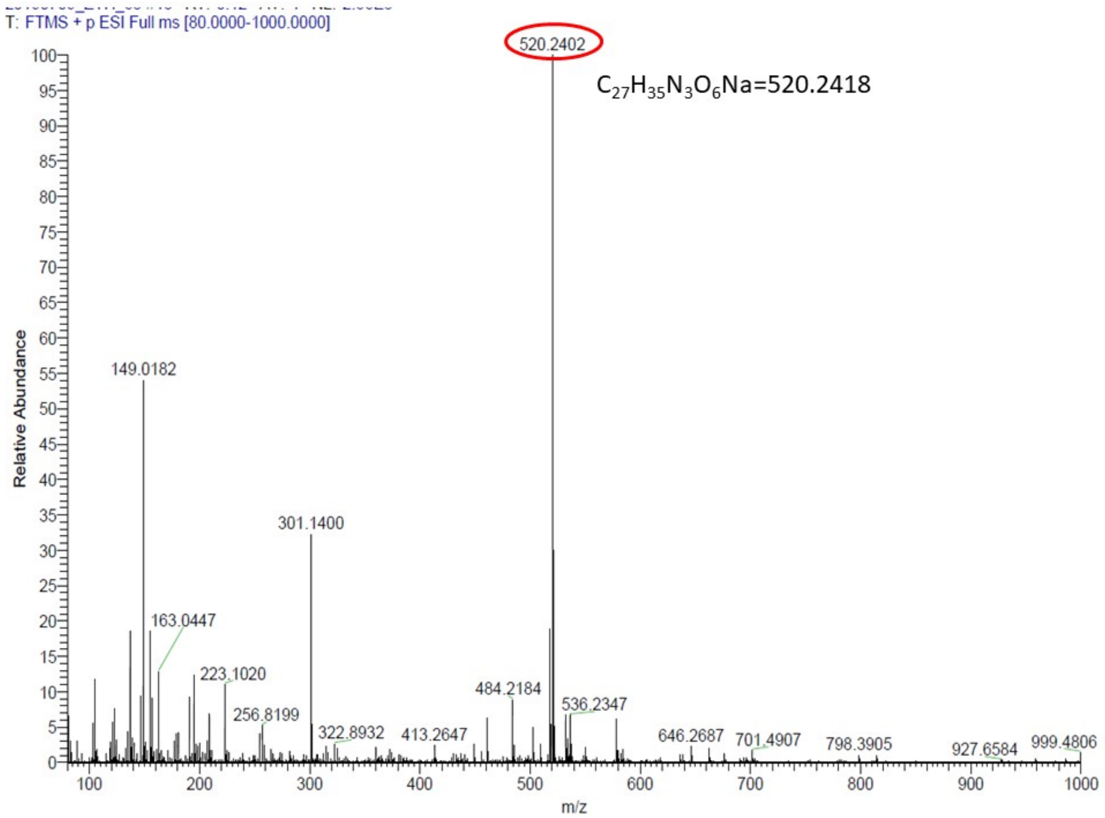


**Fig. S15** HRESIMS spectrum of compound **2**.

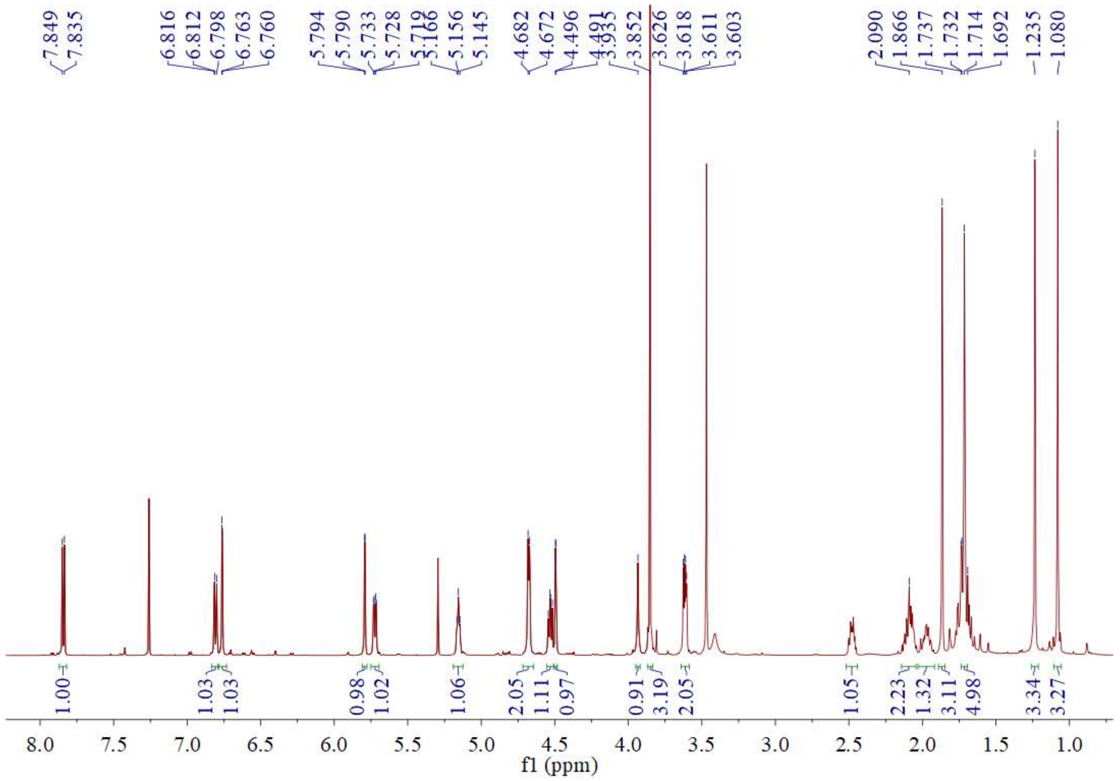


**Fig. S16** $^{1}$H NMR (600 MHz, $CDCl_3$) spectrum of compound **3**.

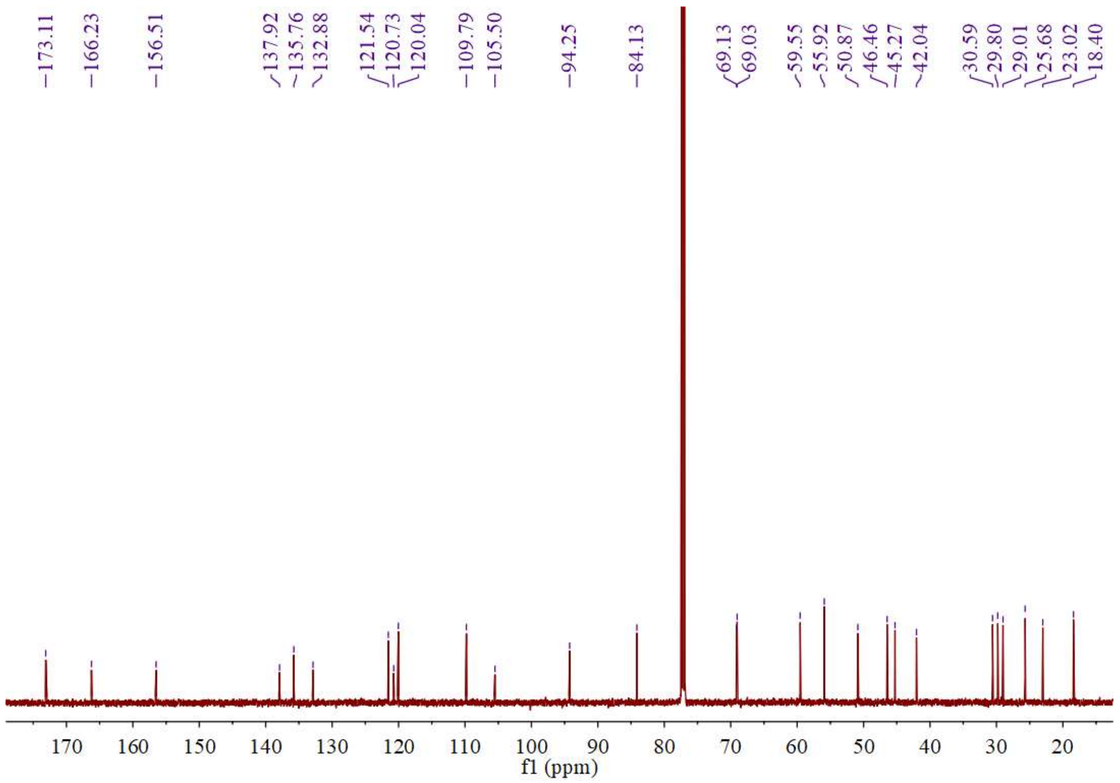


**Fig. S17** $^{13}$C NMR (150 MHz, $CDCl_3$) spectrum of compound **3**.

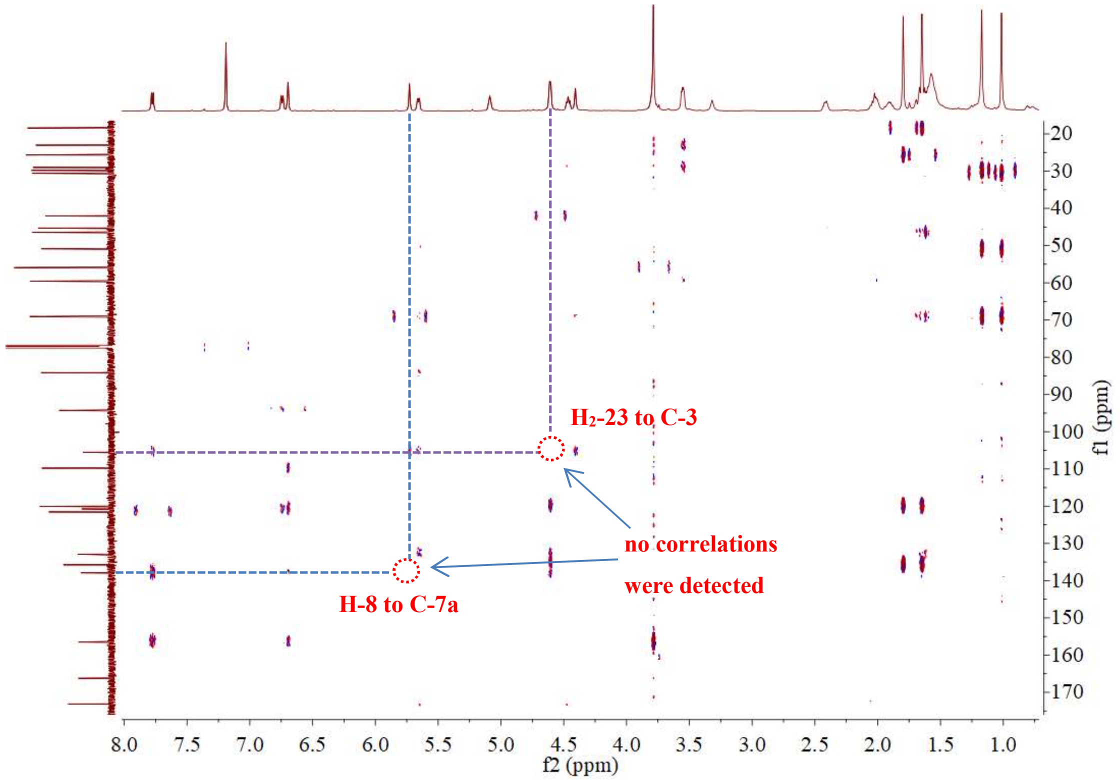


**Fig. S18** HMBC ($CDCl_3$) spectrum of compound **3**.

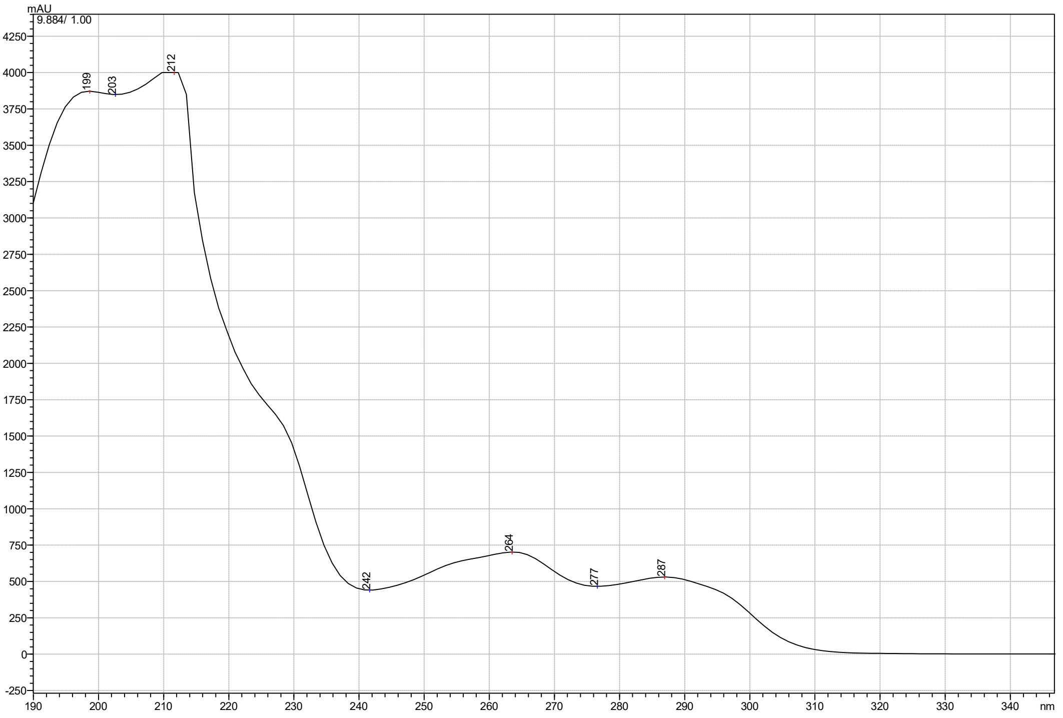


**Fig. S19** Experimental UV spectrum of **1**.

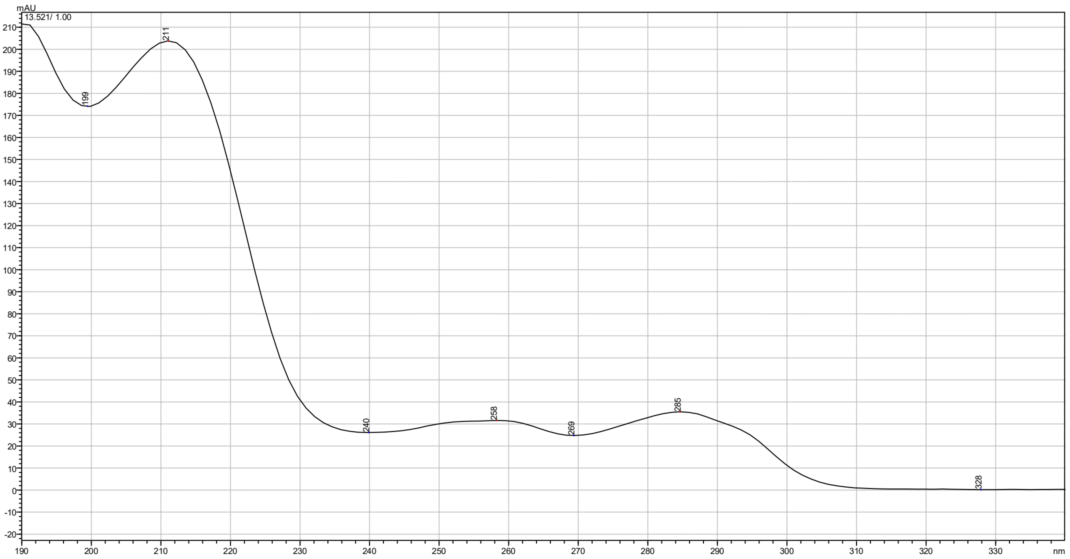


**Fig. S20** Experimental UV spectrum of **2**.

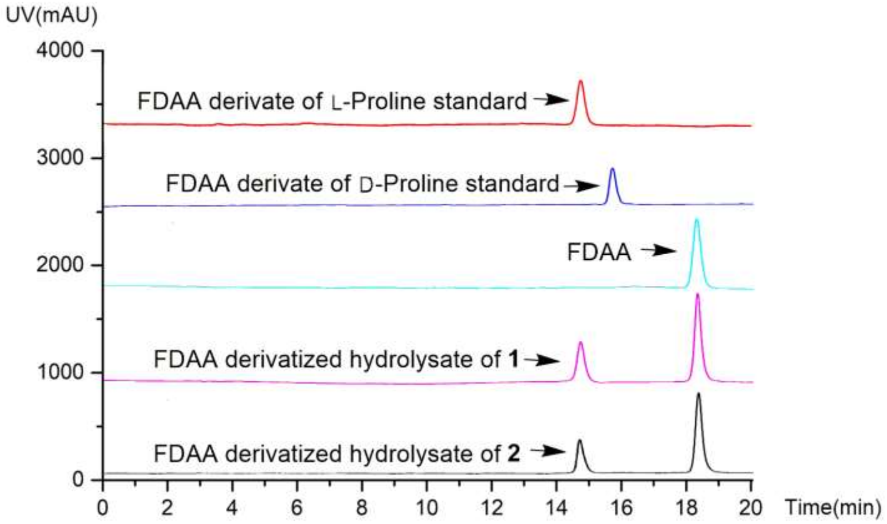


**Fig. S21** HPLC at 254 nm of the Marfey's analysis (FDAA derivate of L-Proline standard $t_R$ 14.5 min; FDAA derivate of D-Proline standard $t_R$ 15.5 min; MeCN-$H_2O$ (35:65, *v*:*v*), *v* = 2.0 mL/min).

**Table S1** The coordinate for the lowest-energy conformer of compound **1** in $^{13}$C NMR, ECD, and ORD calculations

Standard orientation:

| Center Number | Atomic Number | Atomic Type | Coordinates (Angstroms) X | Y | Z |
|---|---|---|---|---|---|
| 1 | 6 | 0 | -4.085536 | 2.717589 | 0.373419 |
| 2 | 6 | 0 | -4.868682 | 1.556209 | 0.188659 |
| 3 | 6 | 0 | -4.285008 | 0.305788 | 0.116656 |
| 4 | 6 | 0 | -2.889102 | 0.185321 | 0.229235 |
| 5 | 6 | 0 | -2.130252 | 1.362427 | 0.416339 |
| 6 | 6 | 0 | -2.700490 | 2.638568 | 0.489242 |
| 7 | 6 | 0 | -1.972016 | -0.933124 | 0.196671 |
| 8 | 6 | 0 | -0.716639 | -0.409388 | 0.362602 |
| 9 | 7 | 0 | -0.799498 | 0.982794 | 0.496991 |
| 10 | 6 | 0 | 0.364072 | 1.816893 | 0.485719 |
| 11 | 6 | 0 | 1.368590 | 1.192157 | -0.518765 |
| 12 | 6 | 0 | 2.690827 | 1.994814 | -0.508653 |
| 13 | 7 | 0 | 1.644355 | -0.218986 | -0.208686 |
| 14 | 7 | 0 | 3.672179 | 1.394514 | -1.190337 |
| 15 | 6 | 0 | 3.532667 | 0.046253 | -1.760820 |
| 16 | 6 | 0 | 2.621624 | -0.851718 | -0.926658 |
| 17 | 8 | 0 | 2.785834 | -2.065990 | -0.929624 |
| 18 | 6 | 0 | 4.986164 | -0.437231 | -1.865766 |
| 19 | 6 | 0 | 5.761866 | 0.862231 | -2.138392 |
| 20 | 6 | 0 | 5.046585 | 1.913981 | -1.276584 |
| 21 | 8 | 0 | 2.796953 | 3.066759 | 0.082057 |
| 22 | 1 | 0 | 3.079888 | 0.113426 | -2.757962 |
| 23 | 6 | 0 | 0.631133 | -1.051871 | 0.517898 |
| 24 | 6 | 0 | -2.343767 | -2.385319 | 0.044204 |
| 25 | 6 | 0 | -2.936083 | -2.708241 | -1.308736 |
| 26 | 6 | 0 | -4.070716 | -3.361074 | -1.589250 |
| 27 | 6 | 0 | -4.486289 | -3.603552 | -3.019785 |
| 28 | 6 | 0 | -5.027198 | -3.915328 | -0.564058 |
| 29 | 8 | 0 | 0.887363 | 1.955528 | 1.788992 |
| 30 | 8 | 0 | 0.852281 | 1.344325 | -1.837836 |
| 31 | 6 | 0 | 0.935694 | -1.299276 | 2.018864 |
| 32 | 6 | 0 | 2.124571 | -2.223705 | 2.403972 |
| 33 | 8 | 0 | 2.169777 | -3.380049 | 1.560327 |
| 34 | 6 | 0 | 3.475561 | -1.489439 | 2.397394 |
| 35 | 6 | 0 | 1.862392 | -2.769851 | 3.810736 |
| 36 | 8 | 0 | -4.792993 | 3.886091 | 0.433868 |
| 37 | 6 | 0 | -4.084225 | 5.099371 | 0.620703 |

| | | | | | |
|---|---|---|---|---|---|
| 38 | 1 | 0 | −5.942768 | 1.678402 | 0.106799 |
| 39 | 1 | 0 | −4.903899 | −0.571997 | −0.033062 |
| 40 | 1 | 0 | −2.088796 | 3.515073 | 0.657501 |
| 41 | 1 | 0 | 0.098414 | 2.793103 | 0.072770 |
| 42 | 1 | 0 | 5.109171 | −1.191483 | −2.642017 |
| 43 | 1 | 0 | 5.297330 | −0.886623 | −0.919154 |
| 44 | 1 | 0 | 6.821424 | 0.780836 | −1.891067 |
| 45 | 1 | 0 | 5.690479 | 1.131731 | −3.196404 |
| 46 | 1 | 0 | 5.046898 | 2.914761 | −1.711382 |
| 47 | 1 | 0 | 5.470687 | 1.988391 | −0.270440 |
| 48 | 1 | 0 | 0.618926 | −2.014332 | 0.007243 |
| 49 | 1 | 0 | −1.449088 | −3.001979 | 0.193141 |
| 50 | 1 | 0 | −3.029191 | −2.668745 | 0.848288 |
| 51 | 1 | 0 | −2.334293 | −2.361981 | −2.148881 |
| 52 | 1 | 0 | −5.463576 | −3.152183 | −3.229415 |
| 53 | 1 | 0 | −3.766414 | −3.193296 | −3.731075 |
| 54 | 1 | 0 | −4.590335 | −4.675698 | −3.225328 |
| 55 | 1 | 0 | −4.743297 | −3.694667 | 0.464798 |
| 56 | 1 | 0 | −6.038344 | −3.522690 | −0.724819 |
| 57 | 1 | 0 | −5.104003 | −5.005090 | −0.657514 |
| 58 | 1 | 0 | 1.599128 | 2.613516 | 1.726715 |
| 59 | 1 | 0 | −0.033491 | 0.952783 | −1.872355 |
| 60 | 1 | 0 | 1.026631 | −0.337986 | 2.527723 |
| 61 | 1 | 0 | 0.026480 | −1.772151 | 2.402663 |
| 62 | 1 | 0 | 2.455247 | −3.117825 | 0.669726 |
| 63 | 1 | 0 | 3.731003 | −1.092766 | 1.414205 |
| 64 | 1 | 0 | 4.269141 | −2.180342 | 2.692761 |
| 65 | 1 | 0 | 3.468934 | −0.652179 | 3.101154 |
| 66 | 1 | 0 | 0.961350 | −3.387584 | 3.813671 |
| 67 | 1 | 0 | 2.699115 | −3.392202 | 4.136447 |
| 68 | 1 | 0 | 1.730034 | −1.959373 | 4.532890 |
| 69 | 1 | 0 | −3.539656 | 5.103536 | 1.571178 |
| 70 | 1 | 0 | −3.383848 | 5.285248 | −0.201098 |
| 71 | 1 | 0 | −4.836712 | 5.885881 | 0.635156 |

**Table S2** The coordinate for the lowest-energy conformer of compound **2** for ECD calculation

Standard orientation:

| Center Number | Atomic Number | Atomic Type | Coordinates (Angstroms) X | Y | Z |
|---|---|---|---|---|---|
| 1 | 6 | 0 | -5.155766 | -0.155648 | -1.965038 |
| 2 | 6 | 0 | -5.855548 | 0.007458 | -0.748145 |
| 3 | 6 | 0 | -5.186694 | -0.003350 | 0.471868 |
| 4 | 6 | 0 | -3.797591 | -0.179202 | 0.434018 |
| 5 | 6 | 0 | -3.074765 | -0.349135 | -0.772925 |
| 6 | 6 | 0 | -3.786051 | -0.335082 | -1.985814 |
| 7 | 7 | 0 | -2.888130 | -0.222643 | 1.476028 |
| 8 | 6 | 0 | -1.621304 | -0.435431 | 0.957725 |
| 9 | 6 | 0 | -1.694350 | -0.511936 | -0.399287 |
| 10 | 6 | 0 | -0.449607 | -0.676276 | -1.195923 |
| 11 | 6 | 0 | 0.562332 | -1.548970 | -0.393104 |
| 12 | 6 | 0 | 1.928889 | -1.510912 | -1.127800 |
| 13 | 7 | 0 | 0.716979 | -1.127209 | 0.997806 |
| 14 | 7 | 0 | 2.959185 | -1.960461 | -0.406826 |
| 15 | 6 | 0 | 2.821563 | -2.409288 | 0.980151 |
| 16 | 6 | 0 | 1.727933 | -1.676959 | 1.745422 |
| 17 | 8 | 0 | 1.771264 | -1.617617 | 2.965234 |
| 18 | 6 | 0 | 4.237314 | -2.221353 | 1.540326 |
| 19 | 6 | 0 | 5.124455 | -2.545808 | 0.326075 |
| 20 | 6 | 0 | 4.352972 | -1.971776 | -0.875749 |
| 21 | 8 | 0 | 2.021845 | -1.130599 | -2.298152 |
| 22 | 1 | 0 | 2.549068 | -3.473708 | 0.996059 |
| 23 | 6 | 0 | -0.380522 | -0.492017 | 1.780778 |
| 24 | 8 | 0 | -7.208909 | 0.170957 | -0.879745 |
| 25 | 6 | 0 | -7.988085 | 0.338436 | 0.290294 |
| 26 | 8 | 0 | -0.733048 | -1.346219 | -2.423317 |
| 27 | 8 | 0 | 0.114677 | -2.906580 | -0.402309 |
| 28 | 6 | 0 | 0.046736 | 0.854305 | 2.435984 |
| 29 | 6 | 0 | 0.260807 | 2.153114 | 1.622853 |
| 30 | 8 | 0 | 1.192590 | 1.810041 | 0.573130 |
| 31 | 6 | 0 | -1.046117 | 2.728909 | 1.051408 |
| 32 | 6 | 0 | 0.885888 | 3.175289 | 2.593104 |
| 33 | 6 | 0 | 1.643638 | 2.869668 | -0.280423 |
| 34 | 6 | 0 | 2.628633 | 2.302630 | -1.256245 |
| 35 | 6 | 0 | 3.854910 | 2.759268 | -1.542423 |
| 36 | 6 | 0 | 4.688897 | 2.087940 | -2.606651 |
| 37 | 6 | 0 | 4.516117 | 3.950197 | -0.895425 |

| | | | | | |
|---|---|---|---|---|---|
| 38 | 1 | 0 | -5.730976 | -0.141218 | -2.883931 |
| 39 | 1 | 0 | -5.705362 | 0.118973 | 1.414671 |
| 40 | 1 | 0 | -3.262264 | -0.473856 | -2.923768 |
| 41 | 1 | 0 | -3.125181 | -0.222589 | 2.454381 |
| 42 | 1 | 0 | 0.023047 | 0.287522 | -1.406345 |
| 43 | 1 | 0 | 4.371766 | -1.183902 | 1.858376 |
| 44 | 1 | 0 | 4.425106 | -2.859680 | 2.402801 |
| 45 | 1 | 0 | 5.235072 | -3.629216 | 0.219523 |
| 46 | 1 | 0 | 6.126763 | -2.122139 | 0.409420 |
| 47 | 1 | 0 | 4.657947 | -0.950646 | -1.120956 |
| 48 | 1 | 0 | 4.448147 | -2.572900 | -1.782428 |
| 49 | 1 | 0 | -0.549485 | -1.159869 | 2.634167 |
| 50 | 1 | 0 | -9.017301 | 0.447897 | -0.047689 |
| 51 | 1 | 0 | -7.916703 | -0.534102 | 0.949682 |
| 52 | 1 | 0 | -7.693732 | 1.237167 | 0.844339 |
| 53 | 1 | 0 | 0.082014 | -1.278450 | -2.948436 |
| 54 | 1 | 0 | -0.415667 | -3.018288 | -1.209651 |
| 55 | 1 | 0 | -0.710999 | 1.075916 | 3.196964 |
| 56 | 1 | 0 | 0.962283 | 0.642306 | 2.989395 |
| 57 | 1 | 0 | -0.890868 | 3.726898 | 0.634397 |
| 58 | 1 | 0 | -1.794579 | 2.828714 | 1.841953 |
| 59 | 1 | 0 | -1.464683 | 2.094755 | 0.270691 |
| 60 | 1 | 0 | 0.261633 | 3.286792 | 3.483828 |
| 61 | 1 | 0 | 1.877101 | 2.845701 | 2.914198 |
| 62 | 1 | 0 | 0.978137 | 4.166527 | 2.143638 |
| 63 | 1 | 0 | 0.788718 | 3.290401 | -0.828705 |
| 64 | 1 | 0 | 2.082327 | 3.678396 | 0.309003 |
| 65 | 1 | 0 | 2.269273 | 1.433660 | -1.802390 |
| 66 | 1 | 0 | 4.181166 | 1.224230 | -3.039177 |
| 67 | 1 | 0 | 4.925365 | 2.785300 | -3.419558 |
| 68 | 1 | 0 | 5.651926 | 1.754301 | -2.200569 |
| 69 | 1 | 0 | 3.954181 | 4.361520 | -0.056937 |
| 70 | 1 | 0 | 5.514952 | 3.684516 | -0.530867 |
| 71 | 1 | 0 | 4.660125 | 4.756952 | -1.624194 |

**Table S3** Cytotoxic activity data of compounds **1** and **2**

| Compd. | $IC_{50}$ (μM) | | | |
|---|---|---|---|---|
| | A549 | HepG2 | AGS | HGC-27 |
| **1** | >30 | >30 | >30 | >30 |
| **2** | >30 | >30 | >30 | >30 |
| **DDP** | 1.26 | 3.25 | 2.43 | 2.50 |
